# Association of primary and community care services with emergency visits and hospital admissions at the end of life in people with cancer: a retrospective cohort study

**DOI:** 10.1136/bmjopen-2021-054281

**Published:** 2022-02-23

**Authors:** Javiera Leniz, Lesley A Henson, Jean Potter, Wei Gao, Tom Newsom-Davis, Zia Ul-Haq, Amanda Lucas, Irene J Higginson, Katherine E Sleeman

**Affiliations:** 1 Cicely Saunders Institute of Palliative Care, Policy and Rehabilitation, King's College London, London, UK; 2 Department of Palliative Care, Hillingdon Hospitals NHS Foundation Trust, Uxbridge, Greater London, UK; 3 Oncology, Chelsea and Westminster Hospital NHS Foundation Trust, London, UK; 4 Discover-Now, Imperial College Health Partners, London, UK

**Keywords:** primary care, palliative care, quality in health care, epidemiology

## Abstract

**Objective:**

To examine the association between primary and community care use and measures of acute hospital use in people with cancer at the end of life.

**Design:**

Retrospective cohort study.

**Setting:**

We used Discover, a linked administrative and clinical data set from general practices, community and hospital records in North West London (UK).

**Participants:**

People registered in general practices, with a diagnosis of cancer who died between 2016 and 2019.

**Primary and secondary outcome measures:**

≥3 hospital admissions during the last 90 days, ≥1 admissions in the last 30 days and ≥1 emergency department (ED) visit in the last 2 weeks of life.

**Results:**

Of 3581 people, 490 (13.7%) had ≥3 admissions in last 90 days, 1640 (45.8%) had ≥1 admission in the last 30 days, 1042 (28.6%) had ≥1 ED visits in the last 2 weeks; 1069 (29.9%) had more than one of these indicators. Contacts with community nurses in the last 3 months (≥13 vs <4) were associated with fewer admissions in the last 30 days (risk ratio (RR) 0.88, 95% CI 0.90 to 0.98) and ED visits in the last 2 weeks of life (RR 0.79, 95% CI 0.68 to 0.92). Contacts with general practitioners in the last 3 months (≥11 vs <4) was associated with higher risk of ≥3 admissions in the last 90 days (RR 1.63, 95% CI 1.33 to 1.99) and ED visits in the last 2 weeks of life (RR 1.27, 95% CI 1.10 to 1.47).

**Conclusions:**

Expanding community nursing could reduce acute hospital use at the end of life and improve quality of care.

**Strengths and limitations of this study**

- Population-based cohort study using a large and comprehensive data set that holds information on healthcare services use from eight different boroughs in London and over 2 million people.
- Our study examined data from local authorities and general practice records, which provides a unique opportunity to describe community and primary care service utilisation.
- People in the cohort might have not died from cancer but from other conditions, as information on cause of death was not available.
- The overall use of the primary care practice and palliative care community teams are likely to be underestimated in this study due to the methods used to estimate contacts.
- Information on the quality of care or the appropriateness of hospital admissions and emergency department visits was not available and are likely to be confounders.

## Background

While mortality rates for most types of cancer have decreased, globally deaths from cancer increased by 25.4% between 2007 and 2017 due to population ageing and growth.^1^ A similar pattern is observed in the UK, and more than 95 000 deaths due to cancer are projected for 2035, 24.5% more than in 2014.^2^ It is therefore critical to understand how to provide high-quality end-of-life care for people with cancer.

Excessive use of hospital care in the last months of life has been proposed as an indicator of the quality of end-of-life cancer care.^3^ This is because emergency hospital care is associated with reduced quality of life and care satisfaction in cancer patients and their families,^4–6^ without contributing to an improvement in survival.^7 8^ Despite these negative outcomes, hospital admissions and emergency department (ED) visits at the end of life have increased over time in cancer patients nearing the end of their life.^9^

Sociodemographic and illness-related factors have been found to be associated with higher hospital admissions and ED attendances in the last months of life, for example being male, black, having lung cancer and low socioeconomic status.^10–12^ On the other hand, access to palliative care services has been associated with lower acute end-of-life care, suggesting that these services could help prevent unnecessary admissions to hospital.^10 13^ However, little is known about how contacts with general practitioners (GPs) in primary care practices and other community services such as community nurses, community palliative care or rehabilitation teams influence acute care use near the end of life among people with cancer. The aim of this study was to describe the association between primary and community care services use with three measures of acute hospital use for people with cancer at the end of life.

## Methods

### Design and data sources

This is a retrospective cohort study using the Discover dataset, one of Europe’s largest linked longitudinal deidentified datasets that includes 95% of all patients registered with a GP in North West London.^14^ The Discover dataset is a platform that enables researcher access to pseudonymised patient-level data drawn from the Whole Systems Integrated Care local data warehouse for research purposes. Discover dataset is maintained and interrogated on a secure server and extracts of data are then aggregated in compliance with the Information Governance suppression rule where numbers below five are annotated as <5. In this process, the deidentified data are rendered anonymised by stripping out any information that would allow reidentification of an individual’s identity. Discover dataset is accessible via Discover-NOW Health Data Research Hub for Real World Evidence through their data scientist specialists and IG committee-approved analysts, hosted by Imperial College Health Partners.

In June 2019, the database held records for a total of 2.37 million patients spread across eight Clinical Commissioning Groups. The estimated total population for the eight boroughs contributing data to Discover was 2.1 million in mid-2019. Differences in the population estimated and the number of patients in the data set could be explained by people being enrolled in a GP practice contributing to the dataset but whose usual place of residence is in another area. Of 370 health and social care provider organisations from the National Health Service (NHS) in North West London, 359 (97%) have a data sharing agreement and submit their records to the dataset. Organisations feeding records to the dataset include primary care practices, mental health, community trusts and hospital care attended by North West London patients, and exclude private and third sector providers, such as hospices services.^14^ We chose the Discover dataset as it is a comprehensive population-based dataset and provides access to community care records in addition to primary and hospital care records, which are not generally available for other primary care datasets in the UK. The age and gender distribution and prevalence of long-term conditions of the Discover population are similar to the rest of London and the UK.^14^

### Population

Adults (aged 18 or over) included in the Discover dataset with at least one record of a cancer diagnosis recorded at any point from 1st January 2015 onwards in primary care practice or hospital inpatient records using Read Codes and International Statistical Classification of Diseases and Related Health Problems (ICD) 10 codes, respectively (codes available in online supplemental box S1). We included in the cohort people who died between 2016 and 2019 based on the date of death recorded in primary care or hospital records. As we did not have information on the cause of death or cancer severity, we restricted our sample to people who had been identified as having palliative care needs in primary care records at any time based on the quality of outcomes framework (QoF) Read Codes for the Palliative Care register,^15^ to include people whose death could be considered expected rather than sudden (codes available in online supplemental box S1).

10.1136/bmjopen-2021-054281.supp1 Supplementary data

### Outcomes

We evaluated three measures of acute hospital use towards the end of life. We chose these three outcome measures as their prevalence at a population-level can be considered an indicator of end-of-life care quality according to Henson *et al* systematic review,^3^ their focus on acute care use at the end of life and the feasibility to be measured in the data. The three measures were as:

1. Three or more emergency hospital admissions in the last 90 days of life.^16^
2. One or more emergency hospital admissions in the last 30 days of life.^3^
3. One or more ED visits in the last 2 weeks of life.^3^

### Explanatory variables

- Primary care practice contacts: we identified contacts with the primary care practice in the last 90 days of life using a similar approach reported by Kontopantelis *et al*.^17^ We considered only direct consultations such as telephone, face-to-face or home visits and excluded administrative consultations or non-attended appointments. It was not possible to identify whether the contact in the practice was with a doctor or another healthcare professional. Only one consultation in the same day was used to reduce the likelihood of including duplicate records, as it was not possible to determine whether records from the same day correspond to more than one contact or not. This approach has been widely used in research using primary care records in the UK^17 18^ (online supplemental box S2)
- Contacts with other community services: we identified contacts with community nurses, community palliative care teams and rehabilitation teams in the last 90 days of life based on the date of the contact and the description of the service. Contacts with rehabilitation teams included physiotherapy, speech and language and occupational therapy services. We removed non-attendant contacts and duplicates based on the date. We identified individuals who were defined by Discover primary care data set as living in a care home based on the latest patient record (online supplemental box S3).

### Co-variables

- Sociodemographic: age at death, gender, ethnicity and Index of Multiple Deprivation (IMD) were extracted from Discover dataset records for each individual. The 2015 IMD was derived at Lower Super Output Areas from patients’ last address registered in the system and reported according to The English Indices of Deprivation 2015 guidance.^19^
- Illness-related: the number of comorbidities was calculated using the count of 15 QoF chronic diseases (excluding cancer) identified from Read codes in the primary care practice records.^15^ The type of cancer was identified from the primary care practice and hospital in-patient records using Read Codes and ICD-10 codes, respectively (online supplemental box S1). Only 6% of the cohort had more than one cancer recorded and were included in the ‘Other’ category.
- Number of days in hospital: we calculated the number of days patients spent in hospital in the last 90 days of life using in-patient hospital codes for spells’ start dates and discharge dates.

### Analysis

Data were described using count and percentage for categorical variables and mean and SD for continuous variables. A Pearson’s χ^2^ test for the trend for categorical variables and t-test and Wilcoxon rank-sum test for age and days in hospital, respectively, was used to evaluate the association between each variable and the outcomes.

We used generalised estimating equations to estimate the unadjusted and multivariate association between sociodemographic, illness-related factors and contacts with primary and community care services in the last 90 days of life and each of the three indicators separately. We used Poisson family with log link function, exchangeable correlation structure and robust error variance with data clustered in primary care practices where patients were registered. For the multivariate model, we adjusted by age, gender, IMD quintile, care home residence, type of cancer and number of QoF comorbidities. We selected specific comorbidities, and primary and community care services use according to significance in unadjusted analysis (p≤0.05). We excluded ethnicity from the final model to avoid biasing the sample as the variable has a large proportion of missing data. To facilitate interpretation, we categorised primary and community care service contacts based on clinical judgement. Categories were approximately one or fewer contacts per month, more than one contact per month but less than one contact per week, and more than one contact per week, depending on the distribution. Because number of contacts with palliative care and rehabilitation teams were small, we adapted these categories. We included the number of days each person spent in hospital in the last 90 days of life as a continuous variable in the models to account for the fact that if someone is in hospital, they cannot receive care in the community.

We performed four sensitivity analysis: (1) to explore the influence of days in hospital by removing the variable from the model, (2) to understand the impact of categorisation of primary and community care services in the model, we used the same model but with the corresponding primary and community care service use variables as continuous, (3) to explore the impact of restricting the sample to people with a record of cancer diagnosis and identification of palliative care needs in the last 12 months of life (instead of at any time) and (4) to understand the influence of ethnicity in the model.

### Patient and public involvement

The protocol was presented and discussed with patients and public representatives at the beginning of the study. A member of the public with experience caring for a relative who died with cancer joined the Project Advisory Group of the project, reviewing a lay version of the protocol and participated in the interpretation of results.

## Results

### Characteristics of the cohort

We identified 4933 people with a diagnosis of cancer and who died between 2016 and 2019; 3848 (78.0%) of them had a palliative care QoF record in primary care records. After removing 267 people with invalid dates of death and hospital admissions, 3581 people were included in the analysis (online supplemental figure S1). The mean age was 76.6 (SD 13.3), 55.4% were male and 21.3% had four or more comorbidities. The most frequent cancer diagnosis was lung cancer (21.5%) followed by bowel (11.6%) and prostate cancer (8.6%) (table 1).

**Table 1:** Sample characteristics by outcome measure

		All sample		3 or more EHA last 3 months					1 or more EHA last month					1 or more ED visit in the last 2 weeks of life				
				No		Yes			No		Yes			No		Yes		
		n=3581		n=3091		n=490			n=1941		n=1640			n=2539		n=1042		
		N	%	N	%	N	%	P value	N	%	N	%	P value	N	%	N	%	P value
Age	(Mean, SD)	76.61	13.32	77.32	13.06	72.18	14.08	<0.001	77.63	12.92	75.41	13.69	<0.001	77.21	13.31	75.16	13.26	<0.001
Gender	Female	1596	44.6	1394	45.1	202	41.2	0.109	905	46.6	691	42.1	0.007	1155	45.5	441	42.3	0.083
	Male	1985	55.4	1697	54.9	288	58.8		1036	53.4	949	57.9		1384	54.5	601	57.7	
Ethnicity	White	1511	42.2	1330	43.0	181	36.9	0.001	866	44.6	645	39.3	0.006	1114	43.9	397	38.1	<0.001
	Black	232	6.5	195	6.3	37	7.6		115	5.9	117	7.1		147	5.8	85	8.2	
	Asian	490	13.7	396	12.8	94	19.2		240	12.4	250	15.2		313	12.3	177	17.0	
	Mixed	523	14.6	447	14.5	76	15.5		270	13.9	253	15.4		380	15.0	143	13.7	
	Other	177	4.9	149	4.8	28	5.7		104	5.4	73	4.5		127	5.0	50	4.8	
	Missing	648	18.1	574	18.6	74	15.1		346	17.8	302	18.4		458	18.0	190	18.2	
IMD quintile	1 (most deprived)	604	16.9	499	16.1	105	21.4	0.047	319	16.4	285	17.4	0.613	414	16.3	190	18.2	0.536
	2	1135	31.7	983	31.8	152	31.0		612	31.5	523	31.9		804	31.7	331	31.8	
	3	894	25.0	776	25.1	118	24.1		474	24.4	420	25.6		642	25.3	252	24.2	
	4	540	15.1	467	15.1	73	14.9		310	16.0	230	14.0		383	15.1	157	15.1	
	5 (least deprived)	299	8.3	268	8.7	31	6.3		167	8.6	132	8.0		222	8.7	77	7.4	
	Missing	109	3.0	98	3.2	11	2.2		59	3.0	50	3.0		74	2.9	35	3.4	
Lived in care home	No	3389	94.6	2910	94.1	479	97.8	0.001	1797	92.6	1592	97.1	<0.001	2387	94.0	1002	96.2	0.010
	Yes	192	5.4	181	5.9	11	2.2		144	7.4	48	2.9		152	6.0	40	3.8	
Number QoF comorbidities	0	579	16.2	489	15.8	90	18.4	0.184	304	15.7	275	16.8	0.041	398	15.7	181	17.4	0.036
	1	856	23.9	750	24.3	106	21.6		495	25.5	361	22.0		641	25.2	215	20.6	
	2	756	21.1	657	21.3	99	20.2		401	20.7	355	21.6		534	21.0	222	21.3	
	3	628	17.5	529	17.1	99	20.2		353	18.2	275	16.8		445	17.5	183	17.6	
	≥4	762	21.3	666	21.5	96	19.6		388	20.0	374	22.8		521	20.5	241	23.1	
QoF comorbidities	COPD (yes)	535	14.9	456	14.8	79	16.1	0.429	266	13.7	269	16.4	0.024	371	14.6	164	15.7	0.390
	Depression (yes)	625	17.5	539	17.4	86	17.6	0.951	330	17.0	295	18.0	0.439	446	17.6	179	17.2	0.781
	Diabetes (yes)	996	27.8	855	27.7	141	28.8	0.609	522	26.9	474	28.9	0.181	682	26.9	314	30.1	0.047
	Hypertension (yes)	2022	56.5	1753	56.7	269	54.9	0.452	1101	56.7	921	56.2	0.734	1441	56.8	581	55.8	0.585
	Dementia (yes)	328	9.2	300	9.7	28	5.7	0.004	202	10.4	126	7.7	0.005	246	9.7	82	7.9	0.086
	CHD (yes)	689	19.2	596	19.3	93	19.0	0.875	354	18.2	335	20.4	0.098	463	18.2	226	21.7	0.017
Type of cancer	Bowel	416	11.6	374	12.1	42	8.6	0.075	234	12.1	182	11.1	0.215	288	11.3	128	12.3	0.032
	Lung	769	21.5	646	20.9	123	25.1		392	20.2	377	23.0		520	20.5	249	23.9	
	Prostate	309	8.6	260	8.4	49	10.0		169	8.7	140	8.5		223	8.8	86	8.3	
	Breast	237	6.6	205	6.6	32	6.5		117	6.0	120	7.3		155	6.1	82	7.9	
	Pancreas	194	5.4	167	5.4	27	5.5		103	5.3	91	5.5		142	5.6	52	5.0	
	Haematolo gical	137	3.8	114	3.7	23	4.7		75	3.9	62	3.8		95	3.7	42	4.0	
	Other	1519	42.4	1325	42.9	194	39.6		851	43.8	668	40.7		1116	44.0	403	38.7	

CHD, chronic heart disease; COPD, chronic obstructive pulmonary disease; ED, emergency department visits; EHA, emergency hospital admissions; IMD, Index of Multiple Deprivation; QoF, quality of outcomes framework.

Of the 3581 people in the sample, 490 (13.7%) had three or more emergency admissions in last 90 days, 1640 (45.8%) had one or more emergency admissions in the last 30 days and 1042 (28.6%) had one or more ED visits in the last 2 weeks of life (table 1). There was overlap between the three indicators with 1069 (29.9%) of the sample having more than one of the indicators and 269 (7.5%) of the cohort having all three. Older age, white ethnicity and living in a care home were associated with lower chances of all three outcomes (table 1).

### Primary care and other community services

On average, people in the cohort had 2.3 (SD 3.3) telephone and 2.4 (SD 3.3) face-to-face consultations with the primary care practice, 8.6 (SD 13.7) contacts with community nurses, 1.2 (SD 3.0) contacts with community palliative care teams and 0.3 (SD 1.2) contacts with rehabilitation services in the last 90 days of life.

People with three or more hospital admissions in the last 90 days and ED visits in the last 2 weeks of life also had more contacts in the primary care practice. Conversely, people with hospital admissions in the last 30 days and ED visits in the last 2 weeks of life had fewer contacts with community nurses and palliative care teams (table 2).

**Table 2:** Healthcare services utilisation in the last 3 months of life by outcome measure

	3 or more EHA in the last 3 months					1 or more EHA in the last month					1 or more ED visits in the last 2 weeks				
	No		Yes			No		Yes			No		Yes		
	N	%	N	%	P value*	N	%	N	%	P value*	N	%	N	%	P value*
**Contacts with GP practice**															
0–3	1812	58.6	243	49.6		1144	58.9	911	55.5		1499	59.0	556	53.4	
4–10	873	28.2	149	30.4		539	27.8	483	29.5		705	27.8	317	30.4	
≥11	406	13.1	98	20.0	<0.001	258	13.3	246	15.0	0.105	355	14.0	169	16.2	0.014
**Contacts with community nurses**															
0–3	1629	52.7	244	49.8		995	51.3	878	53.5		1300	51.2	573	55.0	
4–12	712	23.0	123	25.1		420	21.6	415	25.3		582	22.9	253	24.3	
≥13	725	23.5	117	23.9	0.475	504	26.0	338	20.6	<0.001	632	24.9	210	20.2	0.008
**Contacts with community palliative care teams**															
0–3	2652	85.8	432	88.2		1644	84.7	1440	87.8		2170	85.5	914	87.7	
4–8	313	10.1	40	8.2		206	10.6	147	9.0		254	10.0	99	9.5	
≥9	126	4.1	18	3.7	0.350	91	4.7	53	3.2	0.017	115	4.5	29	2.8	0.044
**Contacts with rehabilitation teams**															
0	2830	91.6	450	91.8		1760	90.7	1520	92.7		2321	91.4	959	92.0	
1–3	186	6.0	29	5.9		127	6.5	88	5.4		153	6.0	62	6.0	
≥4	75	2.4	11	2.2	0.966	54	2.8	32	2.0	0.082	65	2.6	21	2.0	0.622
**Days in hospital**	**P value†**					**P value**†					**P value**†				
Mean (SD)	11.85	(14.15)	24.98	(13.32)		10.21	(14.9)	17.71	(13.48)		13.1	(15.21)	14.98	(13.46)	
Median (IQR)	7	(0.0–19.0)	23	(15.0–32.0)	<0.001	2	(0.0–16.0)	15	(8.0–25.0)	<0.001	8	(0.0–20.0)	11	(5.0–21.0)	<0.001

*χ^2^ for trend p value.

†Wilcoxon rank-sum test p value.

ED, emergency department visits; EHA, emergency hospital admissions; GP, general practitioner.

### Multivariate analysis

In the multivariate analysis, people with three or more hospital admissions in the last 90 days were more likely to be younger, have lung or prostate cancer, have more than 11 contacts with the primary care practice in the last 90 days and were less likely to live in a care home. People with one or more admissions in the last 30 days, were more likely to be younger, male, have breast cancer, they were less likely to live in a care home, and less likely to have more than 13 contacts with community nurses in the last 90 days. Having one or more ED visits in the last 2 weeks of life was associated with being younger, having fewer contacts with community nurses, more contacts with the primary care practice and lower chances of living in a care home (table 3).

**Table 3:** Association between sociodemographic, illness-related and service-related factors with three outcome measures for acute end-of-life care in the last 3 months of life

	3 or more EHA last 3 months				1 or more EHA last month				1 or more ED last 2 weeks			
	n=3472				n=3441				n=3441			
	Univariate		Multivariate		Univariate		Multivariate		Univariate		Multivariate	
	RR	95% CI	RR	95% CI	RR	95% CI	RR	95% CI	RR	95% CI	RR	95% CI
Age	0.98	(0.97 to 0.99)	0.98	(0.97 to 0.99)	0.99	(0.99 to 0.10)	0.99	(0.99 to 1.00)	0.99	(0.99 to 1.00)	0.99	(0.99 to 1.00)
Gender (ref. female)												
Male	1.15	(0.97 to 1.36)	1.10	(0.92 to 1.31)	1.10	(1.02 to 1.20)	1.11	(1.02 to 1.20)	1.09	(0.98 to 1.21)	1.10	(0.98 to 1.23)
IMD quintile (ref. 1, most deprived)												
2	0.79	(0.63 to 0.99)	0.82	(0.65 to 1.03)	1.00	(0.90 to 1.12)	1.01	(0.91 to 1.12)	0.97	(0.84 to 1.11)	1.01	(0.89 to 1.15)
3	0.80	(0.62 to 1.02)	0.88	(0.70 to 1.12)	1.01	(0.90 to 1.13)	1.03	(0.92 to 1.15)	0.90	(0.77 to 1.06)	0.93	(0.80 to 1.15)
4	0.82	(0.62 to 1.08)	0.96	(0.75 to 1.24)	0.92	(0.81 to 1.05)	0.96	(0.85 to 1.09)	0.95	(0.80 to 1.13)	1.00	(0.84 to 1.18)
5	0.68	(0.49 to 0.93)	0.95	(0.68 to 1.31)	0.97	(0.81 to 1.16)	1.06	(0.90 to 1.25)	0.88	(0.71 to 1.10)	0.98	(0.79 to 1.22)
Lived in care home (ref. no)	0.43	(0.24 to 0.78)	0.53	(0.28 to 0.98)	0.54	(0.41 to 0.72)	0.54	(0.41 to 0.72)	0.71	(0.51 to 0.99)	0.70	(0.49 to 0.99)
Type of cancer (ref. bowel)												
Lung	1.53	(1.13 to 2.06)	1.60	(1.16 to 2.20)	1.11	(0.98 to 1.25)	1.08	(0.96 to 2.23)	1.04	(0.88 to 1.22)	1.01	(0.85 to 1.19)
Prostate	1.57	(1.09 to 2.28)	1.57	(1.07 to 2.30)	1.03	(0.89 to 1.21)	0.98	(0.84 to 2.15)	0.91	(0.74 to 1.11)	0.89	(0.72 to 1.10)
Breast	1.32	(0.88 to 1.97)	1.24	(0.82 to 1.87)	1.15	(0.98 to 1.34)	1.19	(1.02 to 1.39)	1.11	(0.93 to 1.34)	1.16	(0.94 to 1.42)
Pancreas	1.34	(0.88 to 2.04)	1.23	(0.93 to 2.08)	1.05	(0.89 to 1.25)	1.02	(0.87 to 2.20)	0.85	(0.67 to 1.09)	0.82	(0.63 to 1.06)
Haematological	1.63	(1.01 to 2.61)	1.23	(0.73 to 2.07)	1.03	(0.83 to 1.29)	0.91	(0.72 to 2.15)	0.98	(0.74 to 1.31)	0.93	(0.68 to 1.26)
Other	1.24	(0.91 to 1.69)	1.15	(0.83 to 1.58)	1.00	(0.88 to 1.12)	0.96	(0.86 to 1.08)	0.85	(0.73 to 0.99)	0.82	(0.70 to 0.96)
Number QoF comorbidities (ref. 0)												
1	0.81	(0.64 to 1.03)	0.88	(0.69 to 1.11)	0.90	(0.81 to 1.00)	0.92	(0.83 to 1.02)	0.82	(0.70 to 0.96)	0.88	(0.75 to 1.04)
2	0.83	(0.65 to 1.07)	0.99	(0.76 to 1.30)	0.99	(0.89 to 1.10)	1.06	(0.95 to 1.18)	0.95	(0.81 to 1.11)	1.05	(0.89 to 1.25)
3	1.01	(0.79 to 1.29)	1.23	(0.94 to 1.62)	0.92	(0.82 to 1.04)	0.99	(0.87 to 1.12)	0.93	(0.79 to 1.10)	1.01	(0.84 to 1.21)
≥4	0.80	(0.62 to 1.04)	0.98	(0.74 to 1.30)	1.04	(0.92 to 1.17)	1.11	(0.97 to 1.27)	1.01	(0.86 to 1.18)	1.09	(0.89 to 1.32)
QoF comorbidities (ref. no)												
COPD	1.09	(0.88 to 1.34)	*	*	1.11	(1.01 to 1.23)	1.07	(0.97 to 1.19)	1.05	(0.91 to 1.21)	*	*
Depression	1.01	(0.84 to 1.22)	*	*	1.04	(0.94 to 1.14)	*	*	0.98	(0.86 to 1.11)	*	*
Diabetes	1.02	(0.86 to 1.22)	*	*	1.04	(0.97 to 1.12)	*	*	1.09	(0.97 to 1.22)	*	*
Hypertension	0.92	(0.79 to 1.07)	*	*	0.98	(0.92 to 1.05)	*	*	0.96	(0.87 to 1.07)	*	*
Dementia	0.62	(0.44 to 0.88)	0.78	(0.54 to 1.14)	0.84	(0.73 to 0.97)	0.94	(0.82 to 1.07)	0.86	(0.70 to 1.06)	*	*
CHD	0.99	(0.82 to 1.20)	*	*	1.08	(0.99 to 1.18)	*	*	1.16	(1.03 to 1.30)	1.14	(0.99 to 1.31)
Contacts with GP practice (ref. 0–3)												
4–10	1.21	(1.01 to 1.46)	1.18	(0.98 to 1.41)	1.05	(0.97 to 1.13)	*	*	1.12	(1.00 to 1.24)	1.10	(0.98 to 1.22)
≥11	1.59	(1.29 to 1.95)	1.63	(1.33 to 1.99)	1.08	(0.98 to 1.19)	*	*	1.19	(1.03 to 1.38)	1.27	(1.10 to 1.47)
Contacts with community nurses (ref. 0–3)												
4–12	1.08	(0.90 to 1.29)	*	*	1.04	(0.96 to 1.13)	1.06	(0.98 to 1.15)	0.97	(0.86 to 1.10)	0.96	(0.85 to 1.08)
≥13	1.01	(0.83 to 1.24)	*	*	0.84	(0.76 to 0.93)	0.88	(0.90 to 0.98)	0.80	(0.69 to 0.93)	0.79	(0.68 to 0.92)
Contacts with community palliative care teams (ref. 0–3)												
4–8	0.88	(0.68 to 1.15)	*	*	0.91	(0.80 to 1.04)	0.95	(0.82 to 1.08)	0.97	(0.82 to 1.14)	1.01	(0.85 to 1.21)
≥9	0.93	(0.62 to 1.37)	*	*	0.78	(0.63 to 0.96)	0.85	(0.69 to 1.04)	0.70	(0.50 to 0.97)	0.78	(0.56 to 1.08)
Contacts with rehabilitation teams (ref. 0)												
1–3	1.01	(0.73 to 1.38)	*	*	0.89	(0.76 to 1.05)	*	*	1.01	(0.82 to 1.25)	*	*
≥4	0.93	(0.55 to 1.58)	*	*	0.80	(0.61 to 1.04)	*	*	0.81	(0.57 to 1.15)	*	*
Days in hospital	0.94	(0.94 to 0.95)	1.04	(1.03 to 1.04)	1.02	(1.01 to 1.02)	1.02	(1.01 to 1.02)	1.00	(0.99 to 1.00)	1.00	(1.00 to 1.01)

*Variable not included in the model.

CHD, chronic heart disease; COPD, chronic obstructive pulmonary disease; ED, emergency department visit; EHA, emergency hospital admissions; IMD, Index of Multiple Deprivation; QoF, quality of outcomes framework; RR, risk ratio.

The sensitivity analyses for the outcomes three or more hospital admissions in the last 90 days and ED visits demonstrated similar results (online supplemental tables S1 and S3). In the sensitivity analyses for one or more admissions in the last 30 days, the number of contacts with community nurses analysed as a continuous variable was no longer associated with the outcome measure, suggesting a threshold response (online supplemental table S2).

## Discussion

The three outcome measures for end-of-life cancer care evaluated (three or more admissions to hospital in the last 90 days, one or more hospital admissions in the last 30 days and one or more ED visits in the last 2 weeks of life) were frequent (13.7%, 45.8% and 28.6%, respectively). We found that contacts with community nurses were associated with fewer ED visits in the last 2 weeks of life, and contacts with the primary care practice were associated with higher risk of multiple admissions to hospital in the last 90 days and ED visits in the last 2 weeks of life.

We found contacts with community nurses were associated with a lower risk of hospital admissions and ED visits at the end of life. These findings are consistent with previous studies where more community nursing hours per week were associated with lower odds of hospital admissions and ED visits at the end of life among patients with cancer in Canada.^20 21^ Community nurses have an important role providing physical care, managing symptoms and medications, educating and giving information to patients and families, and coordinating care^22–25^ and therefore, they could play an important role in avoiding unnecessary hospital admissions at the end of life in people with cancer.

People with cancer who were living in care homes before death had a lower risk of hospital admissions and ED visits at the end of life in this cohort. The number of people living in care homes in our sample was small, and we did not have information on the level of healthcare needs of this group of people. Nevertheless, these findings are consistent with other international studies showing that people living in long-term facilities are less likely to have transitions to hospital regardless of the cause of death.^26 27^ Long-term facilities are one of the very few care settings in the community providing continuous care including out-of-hours.^28 29^ More research is needed to understand the mechanisms that explain this association, as well as to explore differences in healthcare provision and healthcare needs between people living in care homes and in the community.

In contrast with previous studies,^20 30^ we found that contacts with the primary care practice were associated with higher risk of multiple admissions to hospital in the last 90 days and ED visits in the last 2 weeks of life. This is likely to be explained by complexity of healthcare needs: more severe and complex patients are likely to have a higher use of healthcare services.^31^ A rise in the number of contacts with the practice could be an opportunity to identify patients who are deteriorating or whose healthcare needs are increasing. High healthcare use can also be an indicator of unmet needs, ineffective and uncoordinated care and lead to poor patient satisfaction.^32–34^ Having many different healthcare professionals could cause confusion among patients and their caregivers and lead to more consultations. It is possible that more contacts with the primary care practice in this sample reflects poor coordination or a lack of continuity of care, leading to more admissions to hospital.

### Implications for research and/or practice

Primary care physicians play a key role in providing care for people approaching the end of life. Their involvement is valued by patients and families,^35^ and has shown to improve end-of-life care outcomes.^36 37^ However, several barriers to palliative care in general practice have been identified, such as the increasing workload and time, lack of funding, poor communication with specialists and lack of experience and training.^38 39^ More research is needed to explore effective models of end-of-life care in primary care and palliative care integration in order to address the increasing demand for care and complexity of healthcare needs that patients experience when approaching the end of life.

The three measures used in this study have been proposed as quality indicators for cancer end-of-life care.^3 40^ Measuring the quality of care provided by healthcare services is key to monitor and promote the delivery of high-quality cancer care. We found an overlap between these indicators, with 29.9% patients having more than one and different predictors associated with each of them, which suggests a balanced combination of quality indicators might be needed to measure the quality of care provided by healthcare services. Although the measures chosen are recognised quality indicators and important when evaluating quality, they only represent one component of quality at a population level and should be considered alongside other measures of quality such as patient experience and patient-reported outcome measures.

### Strengths and limitations

The Discover dataset holds comprehensive information on healthcare services use from eight different boroughs in London and over 2 million people, including information on primary, community and hospital care. However, our cohort is limited to a London population, which could limit the generalisability of the results.

A limitation of this study is the lack of information on cause of death, time for diagnosis and stage of cancer. Some of the people included might have not died from cancer but from other conditions, and this could vary between different cancer groups. We tried to address this limitation by restricting the sample to people who had been identified as having palliative care needs. However, that approach could have biased the sample towards people with higher or more complex healthcare needs. We derived the date of death from primary care and hospital records, therefore some level of inaccuracy might be expected.^41^ Primary care practice contacts were derived from Read codes and therefore it is possible that the number of consultations with the practice was underestimated.^17 18^ We excluded administrative contacts and same day records with the primary care practice, as it has been done in other studies, for technical reasons. This approach might underestimate the overall contribution of primary care practices in this study.

We did not have information on the quality of care, continuity, coordination of care or the appropriateness of hospital admissions. Likewise, it was not possible to determine the length of stay or how close to death a person was admitted to the care home facility. These factors could also have an impact on the outcomes of this study. It is likely that some palliative care services were not fully identified, as community palliative care is often provided by the third sector in England, and therefore not consistently included in administrative records.

## Conclusions

In this population-based cohort study of people with cancer, multiple emergency admissions to hospital in the last 90 days, admissions in the last 30 days and ED visits in the last 2 weeks of life were frequent. Contacts with community nurses were associated with fewer hospital admissions in the last 30 days of life and fewer ED visits in the last 2 weeks of life. More research is needed to explore effective models of end-of-life care in primary care and palliative care integration to address the complexity of the patient population with cancer cared for in primary and community care.

## Supplementary Material

Reviewer comments

Author's manuscript

## Data Availability

Data may be obtained from a third party and are not publicly available. The data that support the findings of this study are available from the Discover data set but restrictions apply to the availability of these data, which were used under license for the current study, and so are not publicly available.

## References

[1] GBD 2017 Causes of Death Collaborators. Global, regional, and national age-sex-specific mortality for 282 causes of death in 195 countries and territories, 1980-2017: a systematic analysis for the global burden of disease study 2017. Lancet 2018;392:1736–88. 10.1016/S0140-6736(18)32203-7 30496103 PMC6227606

[2] Smittenaar CR, Petersen KA, Stewart K, et al. Cancer incidence and mortality projections in the UK until 2035. Br J Cancer 2016;115:1147–55. 10.1038/bjc.2016.304 27727232 PMC5117795

[3] Henson LA, Edmonds P, Johnston A, et al. Population-Based quality indicators for end-of-life cancer care: a systematic review. JAMA Oncol 2020;6:142-150. 10.1001/jamaoncol.2019.3388 31647512

[4] Ersek M, Miller SC, Wagner TH, et al. Association between aggressive care and bereaved families’ evaluation of end-of-life care for veterans with non-small cell lung cancer who died in Veterans Affairs facilities. Cancer 2017;123:3186–94. 10.1002/cncr.30700 28419414

[5] Wright AA, Keating NL, Ayanian JZ, et al. Family perspectives on aggressive cancer care near the end of life. JAMA 2016;315:284–92. 10.1001/jama.2015.18604 26784776 PMC4919118

[6] Wright AA, Zhang B, Keating NL, et al. Associations between palliative chemotherapy and adult cancer patients’ end of life care and place of death: prospective cohort study. BMJ 2014;348:g1219. 10.1136/bmj.g1219 24594868 PMC3942564

[7] Golan R, Bernstein AN, Gu X, et al. Increased resource use in men with metastatic prostate cancer does not result in improved survival or quality of care at the end of life. Cancer 2018;124:2212–9. 10.1002/cncr.31297 29579318

[8] Temel JS, Greer JA, Muzikansky A, et al. Early palliative care for patients with metastatic Non–Small-Cell lung cancer. N Engl J Med 2010;363:733–42. 10.1056/NEJMoa1000678 20818875

[9] Earle CC, Landrum MB, Souza JM, et al. Aggressiveness of cancer care near the end of life: is it a quality-of-care issue? Journal of Clinical Oncology 2008;26:3860–6. 10.1200/JCO.2007.15.8253 18688053 PMC2654813

[10] Henson LA, Gao W, Higginson IJ, et al. Emergency department attendance by patients with cancer in their last month of life: a systematic review and meta-analysis. JCO 2015;33:370–6. 10.1200/JCO.2014.57.3568 25534384

[11] Koroukian SM, Schiltz NK, Warner DF, et al. Social determinants, multimorbidity, and patterns of end-of-life care in older adults dying from cancer. J Geriatr Oncol 2017;8:117–24. 10.1016/j.jgo.2016.10.001 28029586 PMC5373955

[12] Mercadante S, Masedu F, Valenti M, et al. The characteristics of advanced cancer patients followed at home, but admitted to the hospital for the last days of life. Intern Emerg Med 2016;11:713–8. 10.1007/s11739-016-1402-1 26895033

[13] Abedini NC, Hechtman RK, Singh AD, et al. Interventions to reduce aggressive care at end of life among patients with cancer: a systematic review. Lancet Oncol 2019;20:e627–36. 10.1016/S1470-2045(19)30496-6 31674321

[14] Bottle A, Cohen C, Lucas A, et al. How an electronic health record became a real-world research resource: comparison between London’s Whole Systems Integrated Care database and the Clinical Practice Research Datalink. BMC Med Inform Decis Mak 2020;20:71. 10.1186/s12911-020-1082-7 32312259 PMC7171852

[15] NHS Digital. Business rules for quality and outcomes framework (QOF) 2017/2018, 2017. Available: https://digital.nhs.uk/data-and-information/data-collections-and-data-sets/data-collections/quality-and-outcomes-framework-qof

[16] NHS England. Ccg improvement and assessment framework 2017/18, 2017. Available: https://www.england.nhs.uk/wp-content/uploads/2017/11/ccg-improvement-and-assessment-framework-2017-18.pdf

[17] Kontopantelis E, Olier I, Planner C, et al. Primary care consultation rates among people with and without severe mental illness: a UK cohort study using the clinical practice research Datalink. BMJ Open 2015;5:e008650. 10.1136/bmjopen-2015-008650 PMC4691766 26674496

[18] Browne J, Edwards DA, Rhodes KM, et al. Association of comorbidity and health service usage among patients with dementia in the UK: a population-based study. BMJ Open 2017;7:e012546. 10.1136/bmjopen-2016-012546 PMC5353300 28279992

[19] Government DfCal, Ministry of Housing, Communities & Local Government. The English Index of Multiple Deprivation (IMD) 2015 - Guidance, 2015. Available: https://assets.publishing.service.gov.uk/government/uploads/system/uploads/attachment_data/file/464430/English_Index_of_Multiple_Deprivation_2015_-_Guidance.pdf

[20] Almaawiy U, Pond GR, Sussman J, et al. Are family physician visits and continuity of care associated with acute care use at end-of-life? a population-based cohort study of homecare cancer patients. Palliat Med 2014;28:176–83. 10.1177/0269216313493125 23779252

[21] Seow H, Barbera L, Howell D, et al. Using more end-of-life homecare services is associated with using fewer acute care services: a population-based cohort study. Med Care 2010;48:118–24. 10.1097/MLR.0b013e3181c162ef 20057327

[22] Boot M. Exploring the district nurse role in facilitating individualised advance care planning. Br J Community Nurs 2016;21:144–7. 10.12968/bjcn.2016.21.3.144 26940617

[23] Latter S, Hopkinson JB, Lowson E, et al. Supporting carers to manage pain medication in cancer patients at the end of life: a feasibility trial. Palliat Med 2018;32:246–56. 10.1177/0269216317715197 28679073

[24] Offen J. The role of UK district nurses in providing care for adult patients with a terminal diagnosis: a meta-ethnography. Int J Palliat Nurs 2015;21:134–41. 10.12968/ijpn.2015.21.3.134 25815762

[25] Walshe C, Luker KA. District nurses’ role in palliative care provision: A realist review. Int J Nurs Stud 2010;47:1167–83. 10.1016/j.ijnurstu.2010.04.006 20494357

[26] Pivodic L, Pardon K, Miccinesi G, et al. Hospitalisations at the end of life in four European countries: a population-based study via epidemiological surveillance networks. J Epidemiol Community Health 2016;70:430–6. 10.1136/jech-2015-206073 26584859

[27] Van den Block L, Pivodic L, Pardon K, et al. Transitions between health care settings in the final three months of life in four EU countries. Eur J Public Health 2015;25:569–75. 10.1093/eurpub/ckv039 25829502

[28] Hirakawa Y, Kuzuya M, Uemura K. Opinion survey of nursing or caring staff at long-term care facilities about end-of-life care provision and staff education. Arch Gerontol Geriatr 2009;49:43–8. 10.1016/j.archger.2008.04.010 18538424

[29] Zimmerman S, Sloane PD, Hanson L, et al. Staff perceptions of end-of-life care in long-term care. J Am Med Dir Assoc 2003;4:23–6. 10.1097/01.JAM.0000046935.64053.54 12807593

[30] Kronman AC, Ash AS, Freund KM, et al. Can primary care visits reduce hospital utilization among Medicare beneficiaries at the end of life? J Gen Intern Med 2008;23:1330–5. 10.1007/s11606-008-0638-5 18506545 PMC2518010

[31] Bone AE, Evans CJ, Henson LA, et al. Patterns of emergency department attendance among older people in the last three months of life and factors associated with frequent attendance: a mortality follow-back survey. Age Ageing 2019;48:680–7. 10.1093/ageing/afz043 31127300

[32] Blumenthal D, Abrams MK. Tailoring complex care management for High-Need, high-cost patients. JAMA 2016;316:1657–8. 10.1001/jama.2016.12388 27669168

[33] Salzberg CA, Hayes SL, McCarthy D, et al. Health system performance for the High-Need patient: a look at access to care and patient care experiences. Issue Brief 2016;27:1–12. 27571600

[34] Wennberg JE, Bronner K, Skinner JS, et al. Inpatient Care Intensity And Patients’ Ratings Of Their Hospital Experiences. Health Aff 2009;28:103–12. 10.1377/hlthaff.28.1.103 PMC2729566 19124860

[35] Green E, Knight S, Gott M, et al. Patients’ and carers’ perspectives of palliative care in general practice: A systematic review with narrative synthesis. Palliat Med 2018;32:838–50. 10.1177/0269216317748862 29343169

[36] Kim SL, Tarn DM. Effect of primary care involvement on end-of-life care outcomes: a systematic review. J Am Geriatr Soc 2016;64:1968–74. 10.1111/jgs.14315 27550751

[37] Mitchell G, Aubin M, Senior H, et al. General practice nurses and physicians and end of life: a systematic review of models of care. BMJ Support Palliat Care 2020. 10.1136/bmjspcare-2019-002114. [Epub ahead of print: 27 Jul 2020]. 32718955

[38] Carmont S-A, Mitchell G, Senior H, et al. Systematic review of the effectiveness, barriers and facilitators to general practitioner engagement with specialist secondary services in integrated palliative care. BMJ Support Palliat Care 2018;8:385–99. 10.1136/bmjspcare-2016-001125 28196828

[39] Mitchell S, Loew J, Millington-Sanders C, et al. Providing end-of-life care in general practice: findings of a national GP questionnaire survey. Br J Gen Pract 2016;66:e647–53. 10.3399/bjgp16X686113 27381483 PMC5198709

[40] De Schreye R, Houttekier D, Deliens L, et al. Developing indicators of appropriate and inappropriate end-of-life care in people with Alzheimer’s disease, cancer or chronic obstructive pulmonary disease for population-level administrative databases: A RAND/UCLA appropriateness study. Palliat Med 2017;31:932–45. 10.1177/0269216317705099 28429629

[41] Gallagher AM, Dedman D, Padmanabhan S, et al. The accuracy of date of death recording in the clinical practice research Datalink gold database in England compared with the office for national statistics death registrations. Pharmacoepidemiol Drug Saf 2019;28:563–9. 10.1002/pds.4747 30908785 PMC6593793

