# Supplementary material for: Association of primary and community care services with emergency visits and hospital admissions at the end of life in people with cancer: a retrospective cohort study
Source: BMJ Open. 2022 Feb 23;12(2):e054281. doi: 10.1136/bmjopen-2021-054281 (PMC8867349; doi:10.1136/bmjopen-2021-054281)
Supplement: Supplementary data [file bmjopen-2021-054281supp001.pdf]

BMJ Publishing Group Limited (BMJ) disclaims all liability and responsibility arising from any reliance placed on this supplemental material which has been supplied by the author(s)

Supplementary material

Figure S1. Flowchart of the data management

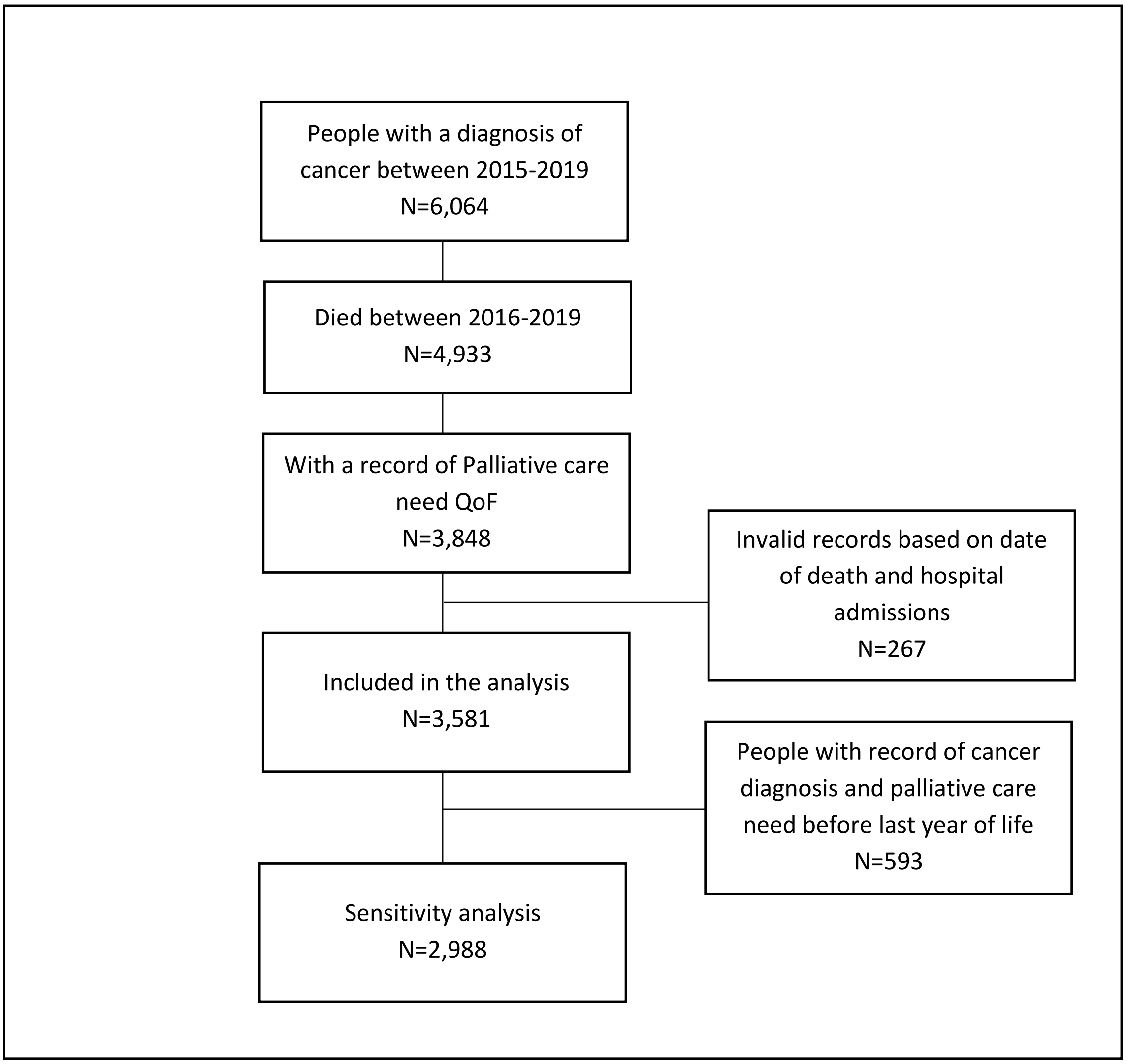

Box S1. List of Read codes and ICD-10 codes used to identify the cohort and variables.

	Read Codes v2	ICD10 codes
Diagnosis of cancer	B0%, B1%, B2%, B3%, B4%, B5%, B6%, Byu%, K1323, K01w1, 68W24, C184., NOT(B677.)	C00 to C97
Type of cancer		
Lung cancer	B22%	C34.0 to C34.9
Bowel cancer	B12% to B14%	C17.0 to C21.8
Prostate cancer	B45%	C61
Breast cancer	B34% to B35%	C50.0 to C50.9
Pancreas cancer	B17%	C25.0 to C25.9
Haematological cancer	B61% to B62% and B64% to B69%	C81.0 to C86.6 and C91.0 to C95.9
Date of death	22J.., 9491., 9495., 94G.., 8HG.., 9493., 94E.., 946.., 94Z.., ZV680, 94…, 949A., 949.., 9431., 9442., 9451., 9452., 9453., 946.., 9492., 9494., 9496., 9497., 9498., 9499., 949B., 949C., 949D., 949E., 949F., 949G., 949H., 949J., 949Z., 94D.., 94G..	
Palliative care QoF	1Z01. , 2JE.. , 2Jf.. , 38VY. , 38Vb. , 38Vd. , 38Ve. , 38Vf. , 38Vg. , 38Vh. , 38Vi. , 8BA2. , 8BAP. , 8BAS. , 8BAT. , 8BAe. , 8BJ1. , 8CM1.% (NOT 8CM15) , 8CM4. , 8CME. , 8CMj. , 8CMk. , 8H6A. , 8H7L. , 8H7g. , 8HH7. , 8IEE. , 9EB5. , 9Ng7. , ZV57C , 8CMQ. , 9NgD. , 9G8.. , 9c0P. , 9c0N. , 8CMW3 , 9K9.. , 9367. , 9c0L0 , 9c0M. , 9NNd. , 8CMb. , 8B2a. , 9NNf0 , 38QH. , 38QK. , 8CMg. , 2Jg.., 9NNq. , 9NNr. , 9NNs.	
Quality of Outcomes Framework (QoF) Rules		
Asthma	H33%, H3120, H3B..,173A., NOT (H333., 21262, 212G.)	
Atrial Fibrillation	G573% NOT (212R.)	
Hypertension	G2..%, G20%, Gyu2., Gyu20, G24..-G2z.., NOT (G24z1, G2400, G2410, G27..)	
Diabetes	C10.., C109J, C109K, C10C., C10D., PKyP., C10Q., C10E%, C10F%, C10H%, C10M%, C10N%, C10P% NOT(C10F8)	
Congestive heart disease	G3…-G309., G30B.-G330z, G33z.-G3401, G342.-G35X., G38..-G3z.., Gyu3%, NOT(Gyu31, G310.)	
Chronic Kidney disease	1Z12., 1Z13. , 1Z14. , 1Z15. , 1Z16. , 1Z1B.-1Z1L. , K053. , K054. , K055. , 1Z1T. , 1Z1V. , 1Z1W. , 1Z1X. , 1Z1Y. , 1Z1Z. , 1Z1a. , 1Z1b. , 1Z1c. , 1Z1d. , 1Z1e. , 1Z1f., 1Z10. , 1Z11. , 1Z17.-1Z1A., K051., K052., 1Z1M., 1Z1Q., 1Z1N., 1Z1P., 1Z1R., 1Z1S. NOT(2126E)	
COPD	H5832, H4640, H4641, Hyu30, Hyu31, H3..%, H31%, H32%, H36..-H3z.., NOT(H3101, H31y0, H3122, H3y0., H3y1.	
Depression	E0013, E0021, E118., E11y2, E11z2, E130, E135., E2003, E291., E2B.., E2B1., Eu204, Eu251, Eu341, Eu412, E112%, E113%, Eu32%, Eu33%, NOT(Eu32a, Eu32B, Eu329, 212S.)	
Epilepsy	F1321, SC200, F25%, NOT(F2501, F2504, F2511, F2516, F25y4, F25G., F25H., 21260, 212J, F256%, F258.-F25A.)	
Heart Failure	G58%, G1yz1, 662f.-662i.	
Peripheral Arterial Disease	G73.., Gyu74, G734., G73y., G73z%, NOT(G73z1)	

Rheumatoid Arthritis	N041., N047., N04X., N04y0, N04y2, Nyu11, Nyu12, Nyu1G, Nyu10, G5yA., G5y8., N040%, N042%, NOT(N0420)	
Stroke	G65..-G654., G656.-G65zz, G63y0.-G63y1,Gyu62-Gyu66, ZV12D, Fyu55, G6760, G6W.., G6X.., Gyu6F, Gyu6G, G61%, G64%, G66%, NOT(G617., G669.)	
Mental Health	E1124, E1134, E11z., E11z0, E11zz, E2122, Eu323, Eu328, Eu333, Eu32A, Eu329, E114.-E117z, E10%, E110%, E111%, E11y%, E12%, E13%, Eu2%, Eu30%, Eu31%, NOT(E11y2, E135.)	
Dementia	F110 to F112, E02y1, E041., Eu041, F116.,F118., F21y2, A410., Eu107, F11x7, Eu02%, E00%, Eu01%, E012%, Eu00%, A411%	

Box S2. List of codes used to derive primary care contacts

	**Read code v2**
Primary care practice face to face contacts	9N1C., 9N1w., 9NF7., 9NF8., 9k27., 9N1G., 9NFB., 9NFW., 9N1t., 9N1x., 9NF5., 9NF4., 9NF6., 982B., 982C., 9N11., 9N12., 9N1c., 9N1y0, 9N1z., 9c0H., 9N01., 9N0G., 9N7B., 9NV.., 9NY.., 9NY0.,
Primary care practice telephone contacts	9N31., 9b0m., 9b0n., 9b0o, 9N310, 9N310, 9N311, 9N3A, 8CAN., 8CAR0
Primary care practice failed contact	9N4.., 9N41%, 9Ni..

Box S3. List of codes used to derive contacts with other community care professionals

	**Service description**	**Service reporting line**
Community nurses contacts	District Nurse<br>District Nurse, Adult, Face to face<br>District Nurse, Adult, Non face to face<br>District Nurses<br>District Nursing Services: Adult<br>Cardiac Nursing / Liaison: Adult<br>Nurse<br>Nursing Services for Children<br>Other Specialist Nursing<br>Other Specialist Nursing, Adult, Face to face<br>Specialist Nursing - Asthma and Respiratory Nursing/Liaison<br>Specialist Nursing - Cardiac Nursing / Liaison<br>Specialist Nursing - Continence Services<br>Specialist Nursing - Diabetic Nursing / Liaison<br>Specialist Nursing - Parkinson''s and Alzheimers Nursing/Liaison<br>Specialist Nursing - Tissue Viability Nursing/Liaison<br>Specialist Nursing - Tuberculosis Specialist Nursing<br>Specialist Nursing, Active Case Management (Community Matrons)<br>Specialist Nursing, Asthma and Respiratory Nursing/Liaison, Adult, Face to face<br>Specialist Nursing, Asthma and Respiratory Nursing/Liaison, Adult, Non face to face<br>Specialist Nursing, Cardiac Nursing/Liaison, Adult, Face to face<br>Specialist Nursing, Cardiac Nursing/Liaison, Adult, Non face to face<br>Specialist Nursing, Continence Services<br>"Specialist Nursing, Continence Services, Adult, Face to face<br>Specialist Nursing, Continence Services, Adult, Non face to face<br>Specialist Nursing, Diabetic Nursing/Liaison<br>Specialist Nursing, Diabetic Nursing/Liaison, Adult, Face to face<br>Specialist Nursing, Diabetic Nursing/Liaison, Adult, Non face to face<br>Specialist Nursing, Parkinson''s and Alzheimers Nursing/Liaison<br>Specialist Nursing, Stoma Care Services, Adult, Face to face<br>Specialist Nursing, Tissue Viability Nursing/Liaison<br>Specialist Nursing, Tissue Viability Nursing/Liaison, Adult, Face to face<br>"Specialist Nursing, Tissue Viability Nursing/Liaison, Adult, Non face to face<br>Tissue Viability Nursing / Liaison: Adult<br>Tuberculosis Special Nursing: Adult	District Nursing<br>District Nursing (H&F, K&C, W)<br>District Nursing (H&F, K&C, W)<br>District Nursing – AWC<br>24 Hour Nursing<br>24 Hour Nursing (Phlebotomy)<br>Adult Nursing<br>CSCNS - Community Nursing<br>Children''s Community Nursing<br>Community Matron<br>Community Matron (H&F, K&C, W)<br>Community Matrons<br>Community Matrons (H&F K&C W)<br>Community Nursing<br>Heart Failure Nursing<br>Heart Nurses (K&C)<br>Night Nursing<br>Night Nursing (K&C)<br>TB Nursing<br>Tissue Viability Nursing<br>Twilight/Night Nursing Service

Palliative care community team contacts	Palliative / Respite Care: Adult<br>Specialist Nursing - Palliative / Respite Care<br>Specialist Nursing, Palliative/Respite Care, Adult, Face to face<br>Specialist Nursing, Palliative/Respite Care, Adult, Non face to face	Palliative Care<br>Palliative Care Service<br>Palliative Medicine (Consultant)<br>Pembridge Bereavement Counselling<br>Pembridge Community<br>Pembridge Day Care
Rehabilitation teams contacts	Community Rehabilitation Teams<br>Other Therapist, Adult, One to One<br>Physiotherapist<br>Physiotherapist, Adult, One to One<br>Physiotherapy<br>Physiotherapy Services: Adult<br>Rehabilitation for Other Disorders<br>Rehabilitation for Other Musculoskeletal Disorders<br>Rehabilitation for Other Neurological Disorders<br>Rehabilitation for Respiratory Disorders<br>SLT - Adult<br>Speech and Language Therapist, Adult, One to One<br>Speech and Language Therapy<br>Occupational Therapist, Adult, One to One<br>Occupational Therapy	Bedded Rehab – Therapists<br>Bedded Rehab - Therapists (H&F, K&C, W)<br>Brent Rehabilitation Service<br>Cardiac Rehabilitation<br>Claypond RehabTherapy<br>Community IFC MSK Physiotherapy Service<br>Community MSK Physiotherapy Service<br>Community Neuro Rehab<br>Community Neuro-Rehabilitation (H&F, K&C, W)<br>Community Recovery Service - Neuro Rehab<br>Community Rehab ICE<br>Community Rehabilitation<br>Community Rehabilitation (H&F, K&C, W)<br>EDTC - Community Physio<br>EHT Therapies<br>Ealing Hospital Therapies<br>Integrated Rehab<br>MSK Physiotherapy<br>Musculoskeletal Service<br>Musculoskeletal Service (W)<br>Physio (MSK)<br>Physiotherapy<br>Pulmonary Rehab<br>Short Term Rehabilitation<br>Therapies MS Physio<br>Adult SLT<br>SLT (Adults)<br>Occupational Therapy

Table S1. Sensitivity analysis for three or more hospital admissions in the last 90 days

	Model 1		Model 2		Model 3		Model 4		Model 5	
	Original Model n=3472		Model 1 without days in hospital n=3472		Model 1 including contacts with PC practice as continu-ous n=3472		Model 1 only for people with a record of cancer diagnosis and identifica-tion of palliative care needs in the last 12 months of life n=2703		Model 1 with ethnicity n-=2841	
	RR	95% CI	RR	95% CI	RR	95% CI	RR	95% CI	RR	95% CI
Age	0.98	(0.97 to 0.99)	0.98	(0.97 to 0.98)	0.98	(0.97 to 0.98)	0.98	(0.97 to 0.98)	0.98	(0.97 to 0.99)
Gender (Male vs female)	1.10	(0.92 to 1.31)	1.11	(0.92 to 1.34)	1.10	(0.92 to 1.02)	1.05	(0.87 to 1.27)	1.06	(0.88 to 1.28)
IMD quintile (Ref=1)										
2	0.82	(0.65 to 1.03)	0.87	(0.70 to 1.09)	0.81	(0.65 to 1.02)	0.87	(0.67 to 1.12)	0.83	(0.65 to 1.05)
3	0.89	(0.70 to 1.12)	0.87	(0.67 to 1.12)	0.88	(0.70 to 1.12)	0.87	(0.66 to 1.13)	0.83	(0.64 to 1.09)
4	0.96	(0.75 to 1.24)	0.90	(0.68 to 1.17)	0.97	(0.75 to 1.24)	1.01	(0.77 to 1.33)	0.97	(0.73 to 1.30)
5	0.95	(0.68 to 1.31)	0.80	(0.58 to 1.11)	0.93	(0.67 to 1.29)	0.90	(0.62 to 1.30)	0.85	(0.56 to 1.28)
Living in care home (Yes vs No)	0.53	(0.28 to 0.98)	0.54	(0.29 to 0.98)	0.52	(0.28 to 0.98)	0.54	(0.28 to 1.05)	0.64	(0.34 to 1.20)
Type of cancer (Ref=Bowel)										
Lung	1.60	(1.16 to 2.20)	1.51	(1.10 to 2.09)	1.61	(1.17 to 2.21)	1.68	(1.17 to 2.41)	1.63	(1.16 to 2.28)
Prostate	1.57	(1.07 to 2.30)	1.81	(1.22 to 2.67)	1.57	(1.07 to 2.30)	1.55	(1.01 to 2.38)	1.64	(1.08 to 2.51)
Breast	1.24	(0.82 to 1.87)	1.33	(0.86 to 2.03)	1.24	(0.82 to 1.87)	1.27	(0.77 to 2.08)	1.15	(0.71 to 1.84)
Pancreas	1.39	(0.93 to 2.08)	1.33	(0.87 to 2.05)	1.42	(0.95 to 2.13)	1.51	(0.96 to 2.37)	1.47	(0.95 to 2.28)
Haematological	1.23	(0.73 to 2.07)	1.82	(1.11 to 2.98)	1.23	(0.73 to 2.08)	1.19	(0.65 to 2.16)	1.13	(0.62 to 2.05)
Other	1.15	(0.83 to 1.58)	1.22	(0.87 to 1.69)	1.15	(0.84 to 1.58)	1.23	(0.86 to 1.78)	1.19	(0.84 to 1.69)
Number of QoF comorbidities (Ref=0)										
1	0.88	(0.69 to 1.11)	0.97	(0.75 to 1.25)	0.87	(0.68 to 1.10)	0.87	(0.67 to 1.13)	0.88	(0.66 to 1.17)
2	0.99	(0.76 to 1.30)	1.08	(0.81 to 1.44)	0.98	(0.75 to 1.29)	0.96	(0.71 to 1.29)	0.89	(0.65 to 1.20)
3	1.23	(0.94 to 1.62)	1.38	(1.04 to 1.83)	1.24	(0.94 to 1.63)	1.18	(0.87 to 1.60)	1.14	(0.84 to 1.55)
>=4	0.98	(0.74 to 1.30)	1.17	(0.88 to 1.57)	0.97	(0.74 to 1.28)	0.91	(0.66 to 1.25)	0.91	(0.66 to 1.26)
Dementia (Yes vs No)	0.78	(0.54 to 1.14)	0.77	(0.53 to 1.13)	0.79	(0.54 to 1.14)	0.80	(0.51 to 1.27)	0.76	(0.49 to 1.16)
Contacts with the primary care practice (Ref= 0 to 3)										
4 to 10	1.18	(0.98 to 1.41)	1.16	(0.97 to 1.39)			1.15	(0.94 to 1.39)	1.20	(0.98 to 1.47)
>=11	1.63	(1.33 to 1.99)	1.52	(1.23 to 1.88)			1.64	(1.32 to 2.04)	1.77	(1.41 to 2.22)
Number of contacts with primary care practice (continuous)					1.02	(1.01 to 1.03)				
Number of days in hospital in the last 90 days	1.04	(1.03 to 1.04)			1.04	(1.03 to 1.04)	1.04	(1.03 to 1.04)	1.04	(1.03 to 1.04)
Ethnicity (Ref= white)										
Black									1.11	(0.82 to 1.50)
Asian									1.52	(1.21 to 1.90)
Mixed									1.19	(0.93 to 1.51)
Other									1.08	(0.73 to 1.60)

Table S2. Sensitivity analysis for one or more hospital admissions in the last 30 days

	**Model 1**		**Model 2**		**Model 3**		**Model 4**		**Model 5**	
	Original Model		Model 1 without days in hospital		Model 1 including contacts with primary care practice as continuous		Model 1 for people with a record of cancer diagnosis and identification of palliative care needs in the last 12 months of life		Model 1 with ethnicity	
	n=3441		n=3441		n=3441		n=2679		n-=2815	
	RR	95% CI	RR	95% CI	RR	95% CI	RR	95% CI	RR	95% CI
Age	1.00	(0.99 to 0.99)	0.99	(0.99 to 1.00)	1.00	(0.99 to 1.00)	1.00	(0.99 to 1.00)	1.00	(0.99 to 1.00)
Gender (Male vs female)	1.11	(1.02 to 1.20)	1.11	(1.02 to 1.21)	1.11	(1.03 to 1.21)	1.10	(1.00 to 1.20)	1.08	(0.99 to 1.19)
IMD quintile (Ref=1)										
2	1.01	(0.91 to 1.12)	1.03	(0.92 to 1.14)	1.01	(0.91 to 1.12)	1.03	(0.92 to 1.16)	1.05	(0.93 to 1.18)
3	1.03	(0.92 to 1.15)	1.02	(0.91 to 1.15)	1.02	(0.92 to 1.14)	1.06	(0.93 to 1.19)	1.05	(0.92 to 1.17)
4	0.96	(0.85 to 1.09)	0.94	(0.82 to 1.07)	0.96	(0.85 to 1.08)	0.99	(0.86 to 1.13)	0.95	(0.82 to 1.09)
5	1.06	(0.90 to 1.25)	1.02	(0.86 to 1.20)	1.06	(0.90 to 1.26)	1.08	(0.91 to 1.28)	1.06	(0.86 to 1.29)
Living in care home (Yes vs no)	0.54	(0.41 to 0.72)	0.54	(0.40 to 0.71)	0.54	(0.41 to 0.73)	0.58	(0.43 to 0.79)	0.58	(0.44 to 0.80)
Type of cancer (Ref=Bowel)										
Lung	1.09	(0.96 to 2.23)	1.06	(0.93 to 1.21)	1.08	(0.95 to 1.23)	1.06	(0.91 to 1.23)	1.06	(0.92 to 1.22)
Prostate	0.98	(0.84 to 2.15)	1.04	(0.89 to 1.22)	0.98	(0.83 to 1.14)	0.94	(0.78 to 1.14)	1.05	(0.87 to 1.24)
Breast	1.19	(1.02 to 1.39)	1.21	(1.03 to 1.43)	1.18	(1.01 to 1.38)	1.22	(1.02 to 1.47)	1.17	(0.96 to 1.40)
Pancreas	1.02	(0.87 to 2.20)	1.01	(0.85 to 1.20)	1.03	(0.87 to 1.21)	1.05	(0.88 to 1.25)	1.07	(0.90 to 1.29)
Haematological	0.91	(0.72 to 2.15)	1.04	(0.83 to 1.31)	0.91	(0.71 to 1.15)	0.85	(0.65 to 1.11)	0.94	(0.73 to 1.21)
Other	0.96	(0.86 to 1.08)	0.98	(0.87 to 1.11)	0.96	(0.85 to 1.08)	0.94	(0.82 to 1.07)	1.00	(0.87 to 1.14)
Number of QoF comorbidities (Ref=0)										
1	0.92	(0.83 to 1.02)	0.96	(0.86 to 1.07)	0.92	(0.83 to 1.02)	0.93	(0.83 to 1.05)	0.93	(0.81 to 1.05)
2	1.06	(0.95 to 1.18)	1.10	(0.98 to 1.23)	1.06	(0.95 to 1.18)	1.12	(1.00 to 1.26)	1.02	(0.89 to 1.16)
3	0.99	(0.87 to 1.12)	1.03	(0.91 to 1.18)	0.98	(0.87 to 1.11)	0.98	(0.85 to 1.13)	0.99	(0.84 to 1.14)
>=4	1.11	(0.97 to 1.27)	1.19	(1.04 to 1.36)	1.10	(0.96 to 1.26)	1.11	(0.94 to 1.30)	1.07	(0.92 to 1.24)
Dementia (Yes vs No)	0.94	(0.82 to 1.07)	0.91	(0.80 to 1.05)	0.93	(0.82 to 1.07)	0.91	(0.78 to 1.06)	0.95	(0.81 to 1.09)
COPD (Yes vs No)	1.07	(0.97 to 1.19)	1.05	(0.95 to 1.17)	1.07	(0.97 to 1.18)	1.08	(0.96 to 1.20)	1.11	(0.98 to 1.25)
Contacts with community nurses (Ref= 0 to 3)										
4 to 12	1.06	(0.98 to 1.15)	1.05	(0.97 to 1.14)			1.07	(0.98 to 1.18)	1.03	(0.94 to 1.14)
>=13	0.88	(0.90 to 0.98)	0.85	(0.76 to 0.95)			0.88	(0.78 to 0.98)	0.84	(0.76 to 0.92)
Contacts with community palliative care teams (Ref= 0 to 3)										
4 to 8	0.95	(0.82 to 1.08)	0.91	(0.80 to 1.04)			0.94	(0.81 to 1.09)	0.90	(0.77 to 1.06)
>=9	0.85	(0.69 to 1.04)	0.79	(0.63 to 0.99)			0.92	(0.74 to 1.14)	0.89	(0.70 to 1.12)
Days in hospital in the last 90 days	1.02	(1.01 to 1.02)			1.02	(1.01 to 1.02)	1.01	(1.01 to 1.02)	1.02	(1.01 to 1.02)
Contacts with community nurses (Continuous)					1.00	(0.99 to 1.00)				
Contacts with community palliative care teams (Continuous)					0.99	(0.97 to 1.00)				
Ethnicity (Ref= white)										
Black									1.12	(0.97 to 1.31)
Asian									1.17	(1.06 to 1.30)
Mixed									1.14	(1.03 to 1.28)
Other									0.91	(0.76 to 1.09)

Table S3. Sensitivity analysis for one or more ED visits in the last 2 weeks of life

	Model 1		Model 2		Model 3		Model 5		Model 5	
	Original Model		Model 1 without days in hospital		Model 1 including contacts with PC practice as continuous		Model 1 for people with a record of cancer diagnosis and identification of palliative care needs in the last 12 months of life		Model 1 with ethnicity	
	n=3441		n=3441		n=3441		n=2679		n-=2815	
	RR	95% CI	RR	95% CI	RR	95% CI	RR	95% CI	RR	95% CI
Age	0.99	(0.99 to 0.99)	0.99	(0.99 to 1.00)	0.99	(0.99 to 1.00)	0.99	(0.99 to 1.00)	0.99	(0.99 to 1.00)
Gender (Male vs female)	1.10	(0.98 to 1.23)	1.10	(0.98 to 1.23)	1.10	(0.98 to 1.23)	1.02	(0.89 to 1.17)	1.09	(0.96 to 1.24)
IMD quintile (Ref=1)										
2	1.01	(0.89 to 1.15)	1.01	(0.89 to 1.15)	1.00	(0.88 to 1.15)	1.03	(0.88 to 1.20)	1.03	(0.88 to 1.21)
3	0.93	(0.80 to 1.15)	0.93	(0.79 to 1.09)	0.93	(0.79 to 1.08)	0.91	(0.75 to 1.10)	0.94	(0.79 to 1.13)
4	1.00	(0.84 to 1.18)	0.99	(0.83 to 1.17)	1.00	(0.84 to 1.18)	0.99	(0.81 to 1.22)	0.97	(0.79 to 1.18)
5	0.99	(0.79 to 1.22)	0.97	(0.78 to 1.21)	0.98	(0.78 to 1.19)	1.03	(0.81 to 1.30)	0.93	(0.70 to 1.23)
Living in care home (Yes vs no)	0.70	(0.49 to 0.99)	0.69	(0.49 to 0.98)	0.70	(0.49 to 1.19)	0.71	(0.49 to 1.02)	0.76	(0.51 to 1.13)
Type of cancer (Ref=Bowel)										
Lung	1.01	(0.85 to 1.19)	1.00	(0.84 to 1.19)	1.01	(0.85 to 1.20)	1.03	(0.85 to 1.26)	0.99	(0.82 to 1.21)
Prostate	0.89	(0.72 to 1.10)	0.90	(0.73 to 1.11)	0.89	(0.72 to 1.09)	0.92	(0.71 to 1.19)	0.91	(0.71 to 1.16)
Breast	1.16	(0.94 to 1.42)	1.17	(0.95 to 1.43)	1.16	(0.94 to 1.42)	1.19	(0.92 to 1.52)	1.14	(0.90 to 1.44)
Pancreas	0.82	(0.63 to 1.06)	0.82	(0.63 to 1.06)	0.82	(0.63 to 1.06)	0.80	(0.60 to 1.07)	0.83	(0.63 to 1.11)
Haematological	0.93	(0.68 to 1.26)	0.97	(0.71 to 1.31)	0.94	(0.69 to 1.27)	0.84	(0.57 to 1.24)	0.90	(0.63 to 1.27)
Other	0.82	(0.70 to 0.96)	0.83	(0.71 to 0.96)	0.83	(0.71 to 0.96)	0.84	(0.69 to 1.01)	0.84	(0.70 to 1.00)
Number of QoF comorbidities (Ref=0)										
1	0.88	(0.75 to 1.04)	0.89	(0.75 to 1.05)	0.88	(0.74 to 1.03)	0.94	(0.78 to 1.13)	0.88	(0.73 to 1.06)
2	1.05	(0.89 to 1.25)	1.06	(0.89 to 1.26)	1.05	(0.89 to 1.25)	1.08	(0.89 to 1.31)	0.95	(0.78 to 1.16)
3	1.01	(0.84 to 1.21)	1.02	(0.85 to 1.23)	1.01	(0.84 to 1.22)	1.03	(0.83 to 1.28)	0.97	(0.79 to 1.20)
>=4	1.09	(0.89 to 1.32)	1.10	(0.90 to 1.34)	1.09	(0.89 to 1.32)	1.11	(0.88 to 1.39)	1.04	(0.83 to 1.30)
Chronic Heart Disease (yes vs no)	1.14	(0.99 to 1.31)	1.14	(1.00 to 1.31)	1.13	(0.99 to 1.30)	1.13	(0.97 to 1.31)	1.16	(1.00 to 1.34)
Contacts with the primary care practice (Ref= 0 to 3)										
4 to 10	1.10	(0.98 to 1.22)	1.10	(0.98 to 1.22)			1.13	(1.00 to 1.27)	1.17	(1.04 to 1.31)
>=11	1.27	(1.10 to 1.47)	1.27	(1.10 to 1.47)			1.35	(1.15 to 1.57)	1.35	(1.14 to 1.60)
Contacts with community nurses (Ref= 0 to 3)										
4 to 12	0.96	(0.85 to 1.08)	0.96	(0.85 to 1.08)			0.92	(0.80 to 1.06)	0.99	(0.87 to 1.14)
>=13	0.79	(0.68 to 0.92)	0.78	(0.67 to 0.91)			0.80	(0.68 to 0.93)	0.74	(0.62 to 0.87)
Contacts with community palliative care teams (Ref= 0 to 3)										
4 to 8	1.01	(0.85 to 1.21)	1.00	(0.84 to 1.20)			1.01	(0.81 to 1.26)	0.91	(0.72 to 1.15)
>=9	0.78	(0.56 to 1.08)	0.76	(0.55 to 1.06)			0.90	(0.66 to 1.24)	0.75	(0.49 to 1.12)
Days in hospital in the last 90 days	1.00	(1.00 to 1.01)			1.00	(1.00 to 1.00)	1.00	(1.00 to 1.01)	1.00	(1.00 to 1.01)
Contacts with primary care practice (continuous)					1.01	(1.01 to 1.01)				
Contacts with community nurses (Continuous)					0.99	(0.99 to 0.99)				
Contacts with community palliative care teams (Continuous)					0.99	(0.97 to 1.01)				
Ethnicity (Ref= white)										
Black									1.28	(1.05 to 1.55)

Asian					1.29	(1.11 to 1.50)
Mixed					0.99	(0.84 to 1.17)
Other					1.00	(0.76 to 1.29)
