# Supplementary material for: Association of primary and community care services with emergency visits and hospital admissions at the end of life in people with cancer: a retrospective cohort study
Source: BMJ Open. 2022 Feb 23;12(2):e054281. doi: 10.1136/bmjopen-2021-054281 (PMC8867349; doi:10.1136/bmjopen-2021-054281)
Supplement: Author's manuscript [file bmjopen-2021-054281.draft_revisions.pdf]

BMJ Open

BMJ Open is committed to open peer review. As part of this commitment we make the peer review history of every article we publish publicly available.

When an article is published we post the peer reviewers' comments and the authors' responses online. We also post the versions of the paper that were used during peer review. These are the versions that the peer review comments apply to.

The versions of the paper that follow are the versions that were submitted during the peer review process. They are not the versions of record or the final published versions. They should not be cited or distributed as the published version of this manuscript.

BMJ Open is an open access journal and the full, final, typeset and author-corrected version of record of the manuscript is available on our site with no access controls, subscription charges or pay-per-view fees (http://bmjopen.bmj.com).

If you have any questions on BMJ Open's open peer review process please email info.bmjopen@bmj.com

BMJ Open

The association of primary and community care services with emergency visits and hospital admissions at the end of life in people with cancer: a retrospective cohort study.

Journal:	*BMJ Open*
Manuscript ID	bmjopen-2021-054281
Article Type:	Original research
Date Submitted by the Author:	08-Jun-2021
Complete List of Authors:	Leniz, Javiera; King's College London, Cicely Saunders Institute of Palliative Care, Policy & Rehabilitation<br>Henson, Lesley; King's College London, Palliative Care, Policy & Rehabilitation<br>Potter, Jean; Hillingdon Hospitals NHS Foundation Trust, Department of Palliative Care<br>Gao, Wei; King's College London, Palliative Care, Policy & Rehabilitation<br>Newsom-Davis, Tom; Chelsea and Westminster Hospital NHS Foundation Trust, Oncology<br>Ul-Haq, Zia; Imperial College Health Partners, Discover-Now<br>Lucas, Amanda; Imperial College Health Partners, Discover-Now<br>Higginson, Irene; King's College London, Palliative Care and Policy<br>Sleeman, Katherine; King's College London, Dept of Palliative Care, Policy & Rehabilitation
Keywords:	PRIMARY CARE, PALLIATIVE CARE, Quality in health care < HEALTH SERVICES ADMINISTRATION & MANAGEMENT, Epidemiology < ONCOLOGY

SCHOLARONE™
Manuscripts

I, the Submitting Author has the right to grant and does grant on behalf of all authors of the Work (as defined in the below author licence), an exclusive licence and/or a non-exclusive licence for contributions from authors who are: i) UK Crown employees; ii) where BMJ has agreed a CC-BY licence shall apply, and/or iii) in accordance with the terms applicable for US Federal Government officers or employees acting as part of their official duties; on a worldwide, perpetual, irrevocable, royalty-free basis to BMJ Publishing Group Ltd ("BMJ") its licensees and where the relevant Journal is co-owned by BMJ to the co-owners of the Journal, to publish the Work in this journal and any other BMJ products and to exploit all rights, as set out in our licence.

The Submitting Author accepts and understands that any supply made under these terms is made by BMJ to the Submitting Author unless you are acting as an employee on behalf of your employer or a postgraduate student of an affiliated institution which is paying any applicable article publishing charge ("APC") for Open Access articles. Where the Submitting Author wishes to make the Work available on an Open Access basis (and intends to pay the relevant APC), the terms of reuse of such Open Access shall be governed by a Creative Commons licence – details of these licences and which Creative Commons licence will apply to this Work are set out in our licence referred to above.

Other than as permitted in any relevant BMJ Author's Self Archiving Policies, I confirm this Work has not been accepted for publication elsewhere, is not being considered for publication elsewhere and does not duplicate material already published. I confirm all authors consent to publication of this Work and authorise the granting of this licence.

For peer review only

Title

The association of primary and community care services with emergency visits and hospital admissions at the end of life in people with cancer: a retrospective cohort study

Authors

1. **Javiera Leniz***

   King's College London, Cicely Saunders Institute of Palliative Care, Policy and Rehabilitation. Bessemer Road, London SE5 0PJ, United Kingdom. javiera.martelli@kcl.ac.uk

2. Lesley A Henson

   King's College London, Cicely Saunders Institute of Palliative Care, Policy and Rehabilitation.

3. Jean Potter

   Department of Palliative Care, The Furze, The Hillingdon Hospital NHS Foundation Trust

4. Wei Gao

   King's College London, Cicely Saunders Institute of Palliative Care, Policy and Rehabilitation.

5. Thomas Newsom-Davis

   Chelsea & Westminster Hospital NHS Foundation Trust

6. Zia Ul-Haq

   Discover-Now, Imperial College Health Partners

7. Amanda Lucas

   Discover-Now, Imperial College Health Partners

8. Irene J Higginson

   King's College London, Cicely Saunders Institute of Palliative Care, Policy and Rehabilitation.

9. Katherine E Sleeman

   King's College London, Cicely Saunders Institute of Palliative Care, Policy and Rehabilitation.

***Corresponding author**

For peer review only - http://bmjopen.bmj.com/site/about/guidelines.xhtml

Abstract

Objective: to examine the association between sociodemographic, illness-related factors and use of primary and community care services with three measures of acute hospital use for people with cancer approaching the end of life.

Design: retrospective cohort study.

Setting: we used Discover, a linked administrative and clinical dataset from general practices, community and hospital records in North West London (UK).

Participants: people registered in general practices, with a diagnosis of cancer who died between 2016-2019.

Primary and secondary outcome measures: >=3 hospital admissions during the last 90 days, >=1 admission in the last 30 days and >=1 emergency department (ED) visit in the last 2 weeks of life.

Results: Of 3581 people, 13.7% had >=3 admissions in last 90 days, 45.8% had >=1 admission in the last 30 days, 28.6% had >=1 ED visit in the last 2 weeks; 29.9% had more than one of these measures. Older age was associated with lower likelihood of these three measures. People with lung (RR 1.60 95% CI 1.16-2.20) and prostate cancer (RR 1.57 95% CI 1.07-2.30) were more likely to have >=3 admissions in last 90 days than people with bowel cancer. Contact with community nurses (>=13 vs <4) was associated with fewer admissions in the last 30 days (RR 0.88, 95% CI 0.79-0.97) and ED visit in the last 2 weeks of life (RR 0.79, 95% CI 0.68-0.92). People living in care homes were less likely to have >=3 admissions in the last 90 days (RR 0.43 95% CI 0.24-0.78), >=1 admission in the last 30 days (RR 0.54 95% CI 0.41-0.72) and ED visits in the last 2 weeks (RR 0.71 95% CI 0.51-0.99).

Conclusions: to reduce acute hospital use at the end of life and improve quality of care, policymakers should consider expanding community nursing and care home services.

Keywords

Primary health care, end-of-life care, inappropriate acute care, hospital admissions, community care, cancer.

Strengths and limitations of this study

- Population-based cohort study using a large and comprehensive dataset that holds information on healthcare service use from eight boroughs in London and over 2 million people.
- Our study examined data from local authorities, hospitals, and general practices, providing an opportunity to describe primary and secondary care service utilization.

For peer review only - http://bmjopen.bmj.com/site/about/guidelines.xhtml

- Use of primary care services and community palliative care may be underestimated due to coding practices and lack of data from voluntary sector providers such as hospices.
- Information on the appropriateness of hospital admissions and ED visits was not collected.

Background

While mortality rates for most types of cancer have decreased, global deaths from cancer increased by 25.4% between 2007 and 2017 due to population ageing and growth.[1] A similar pattern is observed in the United Kingdom and more than 95,000 deaths due to cancer are projected for 2035.[2] It is therefore critical to understand how high-quality end-of-life care for people with cancer is best provided.

Excessive use of hospital care in the last months of life has been proposed as an indicator of the quality of end-of-life cancer care.[3] This is because emergency hospital care is associated with reduced quality of life and lower care satisfaction in cancer patients and their families,[4-6] without contributing to an improvement in survival.[7, 8] Despite these negative outcomes, hospital admissions and emergency department (ED) visits at the end of life have increased over time in this population.[9]

Sociodemographic and illness-related factors have been found to be associated with higher hospital admissions and ED attendances in the last months of life, for example being male, black, having lung cancer and lower socioeconomic status.[10-12] On the other hand, access to palliative care services have been found to be associated with lower acute hospital care, suggesting these services could help prevent unnecessary admissions to hospital.[10, 13] However, little is known about how contacts with general practitioners in primary care practices and other community services such as community nurses, community palliative care or rehabilitation teams influence acute care use near the end of life among people with cancer. The aim of this study was to examine the association between sociodemographic, illness-related factors and use of primary and community care services with three measures of acute hospital use for people with cancer approaching the end of life.

Methods

Design and Data sources

Retrospective cohort study using the Discover dataset, one of Europe's largest linked longitudinal de-identified dataset that includes over 2.6 million patients registered with a general practitioner in North West London. The database is spread across eight Clinical Commissioning Groups (CCGs) accounting for 95% of the total North West London population. This dataset is fed by data from over 400 provider

For peer review only - http://bmjopen.bmj.com/site/about/guidelines.xhtml

organisations including 365 primary care practices, two mental health trusts, two community trusts and all in-hospital records from North West London patients.[14] We chose the Discover dataset as it is a comprehensive population-based dataset and provides access to community care records in addition to primary and hospital care records, which is not generally available for other primary care datasets in the UK. The age, gender distribution and prevalence of long-term conditions of the Discover population are similar to the rest of London and the UK.[14]

The use of Discover dataset is approved for secondary analysis by the West Midland-Solihull Research Ethic Committee (reference 18/WM/0323).

Population

Adults (aged 18 or over) included in the Discover dataset with any cancer diagnosis recorded from 1st January 2015 onwards in primary care practice records or hospital in-patient records, identified using Read Codes and International Statistical Classification of Diseases and Related Health Problems (ICD) 10 codes respectively. We included in the cohort people who died between 2016 and 2019 based on the date of death recorded in primary care or hospital records. We restricted our sample to people who had been identified as having palliative care needs in primary care records at any time based on the Quality of Outcomes Framework (QoF) Read Codes for the Palliative Care register,[15] to include people whose death could be considered expected rather than sudden (Codes available in Supplemental Box S1).

Outcomes

We evaluated three measures of acute hospital use towards the end of life. We chose these three measures as their prevalence at a population-level is an indicator of end of life care quality,[3] their focus on acute care use at the end of life, and the feasibility to be measured in the data. The three measures are:

1. Three or more emergency hospital admissions in the last 90 days of life.[16]

2. One or more emergency hospital admissions in the last 30 days of life. [3]

3. One or more emergency department (ED) visits in the last two weeks of life.[3]

Explanatory variables

Primary care practice contacts: we identified contacts with the primary care practice in the last 90 days of life.[17] We considered only direct consultations such as telephone, face-to-face or home visits, and excluded administrative consultations or non-attended appointments. It was not possible to identify

For peer review only - http://bmjopen.bmj.com/site/about/guidelines.xhtml

whether the contact in the practice was with a doctor or another healthcare professional. As it was not possible to determine whether records from the same day correspond to more than one contact or not, only one consultation in the same day was used to reduce the likelihood of including duplicate records, as used in other research using primary care records in the UK.[17, 18] (Supplemental Box S2)

Contacts with other community services: we identified contacts with community nurses, community palliative care teams and rehabilitation teams in the last 90 days of life based on the date of the contact and the description of the service. Contacts with rehabilitation teams included physiotherapy, speech and language and occupational therapy services. We removed non-attendant contacts and duplicates based on the date. We identified individuals who were defined by Discover primary care dataset as living in a care home based on the latest patient record (Supplemental Box S3).

Co-variables

Sociodemographic: Age at death, gender (female/male), ethnicity (white/black/asian/mixed/other) and Index of Multiple Deprivation (IMD) were extracted from Discover dataset records for each individual. The 2015 Index of Multiple was derived at Lower Super Output Areas (LSOAs) from the patients' last address registered in the system and categorised by quintiles, being 1 most deprived areas and 5 most affluent areas.

Illness-related factors: the number of comorbidities was calculated using the count of 15 QoF chronic diseases (excluding cancer) identified from Read codes in the primary care practice records.[15] The type of cancer was identified from the primary care practice and hospital in-patient records using Read Codes and ICD-10 codes respectively (Supplemental Box S1).

Number of days in hospital: we calculated the number of days patients spent in hospital in the last 90 days of life using in-patient hospital codes for spells' start dates and discharge dates.

Analysis

Data was described using count and percentage for categorical variables and mean and standard deviation for continuous variables.

We used generalized estimating equations (GEE) to estimate the unadjusted and multivariate association between sociodemographic, illness-related factors and contacts with primary and community care services in the last 90 days of life and each of the three measures separately. We used Poisson family with log link function, exchangeable correlation structure and robust error variance with data clustered in primary care practices where patients were registered. For the multivariate model, we adjusted by sociodemographic and clinical characteristics selected according to a priori hypotheses, forcing

For peer review only - http://bmjopen.bmj.com/site/about/guidelines.xhtml

sociodemographic variables into the model, and according to significance in unadjusted analysis ($p<=0.05$). We excluded ethnicity from the final model to avoid biasing the sample as the variable has a large proportion of missing data. We categorized primary and community care services contacts based on clinical judgment. We adjusted all the models for the number of days spent in hospital in the last 90 days of life to account for the fact that if someone is in hospital, they cannot receive care in the community.

We performed four sensitivity analyses: (1) to explore the influence of days in hospital by removing the variable from the model, (2) to understand the impact of categorization of primary and community care services in the model, (3) to explore the impact of restricting the sample to people with a record of cancer diagnosis and identification of palliative care needs in the last 12 months of life (instead of at any time), (4) to understand the influence of ethnicity on the model.

Patient and public involvement

The protocol was presented and discussed with patients and public representatives at the beginning of the study. A member of the public with experience caring for a relative who died with cancer joined the Project Advisory Group of the project, reviewing a lay version of the protocol, making suggestion for the analysis and participated in the interpretation of results.

Results

Characteristics of the cohort

We identified 3848 people with cancer who died between 2016 and 2019. After removing 267 people with invalid dates of death and/or hospital admissions, 3581 people were included in the analysis (Supplemental Figure S1). The mean age at death was 76.6 (SD 13.3), 55.4% were male and 21.3% had four or more comorbidities. The most frequent cancer diagnosis was lung cancer (21.5%) followed by bowel (11.6%) and prostate cancer (8.6%) (Table 1).

Of the 3581 people in the sample, 490 (13.7%) had three or more emergency admissions in last 90 days, 1640 (45.8%) had one or more emergency admissions in the last 30 days, and 1042 (28.6%) had one or more ED visits in the last two weeks of life (Table 1). There was overlap between the three measures with 1069 (29.9%) of the sample having more than one of the measures and 269 (7.5%) of the cohort having all three.

For peer review only - http://bmjopen.bmj.com/site/about/guidelines.xhtml

Table 1. Sample characteristics by measure.

	All sample		Three or more hospital admissions in last 90 days				One or more hospital admission in last 30 days				One or more ED visit in the last two weeks			
			No		Yes		No		Yes		No		Yes	
	n=3,581		n=3091		n=490		n=1941		n=1640		n=2539		n=1042	
	mean	SD	mean	SD	mean	SD	mean	SD	mean	SD	mean	SD	mean	SD
Age	76.61	13.32	77.32	13.06	72.18	14.08	77.63	12.92	75.41	13.69	77.21	13.31	75.16	13.26
	n	%	n	%	n	%	n	%	n	%	n	%	n	%
Gender														
Female	1,596	44.57	1394	45.10	202	41.22	905	46.63	691	42.13	1155	45.49	441	42.32
Male	1,985	55.43	1697	54.90	288	58.78	1036	53.37	949	57.87	1384	54.51	601	57.68
Ethnicity														
White	1,511	42.19	1330	43.03	181	36.94	866	44.62	645	39.33	1114	43.88	397	38.10
Black	232	6.48	195	6.31	37	7.55	115	5.92	117	7.13	147	5.79	85	8.16
Asian	490	13.68	396	12.81	94	19.18	240	12.36	250	15.24	313	12.33	177	16.99
Mixed	523	14.60	447	14.46	76	15.51	270	13.91	253	15.43	380	14.97	143	13.72
Other	177	4.94	149	4.82	28	5.71	104	5.36	73	4.45	127	5.00	50	4.80
Missing	648	18.10	574	18.57	74	15.10	346	17.83	302	18.41	458	18.04	190	18.23
IMD quintile														
1 (Most deprived)	604	16.87	499	16.14	105	21.43	319	16.43	285	17.38	414	16.31	190	18.23
2	1,135	31.70	983	31.80	152	31.02	612	31.53	523	31.89	804	31.67	331	31.77
3	894	24.97	776	25.11	118	24.08	474	24.42	420	25.61	642	25.29	252	24.18
4	540	15.08	467	15.11	73	14.90	310	15.97	230	14.02	383	15.08	157	15.07
5 (Most affluent)	299	8.35	268	8.67	31	6.33	167	8.60	132	8.05	222	8.74	77	7.39
Missing	109	3.04	98	3.17	11	2.24	59	3.04	50	3.05	74	2.91	35	3.36
Number QoF comorbidities														
0	579	16.17	489	15.82	90	18.37	304	15.66	275	16.77	398	15.68	181	17.37
1	856	23.90	750	24.26	106	21.63	495	25.50	361	22.01	641	25.25	215	20.63
2	756	21.11	657	21.26	99	20.20	401	20.66	355	21.65	534	21.03	222	21.31
3	628	17.54	529	17.11	99	20.20	353	18.19	275	16.77	445	17.53	183	17.56
>=4	762	21.28	666	21.55	96	19.59	388	19.99	374	22.80	521	20.52	241	23.13
QoF Comorbidities														
COPD (yes)	535	14.94	456	14.75	79	16.12	266	13.70	269	16.40	371	14.61	164	15.74
Depression (yes)	625	17.45	539	17.44	86	17.55	330	17.00	295	17.99	446	17.57	179	17.18
Diabetes (yes)	996	27.81	855	27.66	141	28.78	522	26.89	474	28.90	682	26.86	314	30.13
Hypertension (yes)	2022	56.46	1753	56.71	269	54.90	1101	56.72	921	56.16	1441	56.75	581	55.76
Dementia (yes)	328	9.16	300	9.71	28	5.71	202	10.41	126	7.68	246	9.69	82	7.87
Chronic Heart disease (yes)	689	19.24	596	19.28	93	18.98	354	18.24	335	20.43	463	18.24	226	21.69
Type of cancer														
Bowel	416	11.62	374	12.10	42	8.57	234	12.06	182	11.10	288	11.34	128	12.28
Lung	769	21.47	646	20.90	123	25.10	392	20.20	377	22.99	520	20.48	249	23.90
Prostate	309	8.63	260	8.41	49	10.00	169	8.71	140	8.54	223	8.78	86	8.25
Breast	237	6.62	205	6.63	32	6.53	117	6.03	120	7.32	155	6.10	82	7.87
Pancreas	194	5.42	167	5.40	27	5.51	103	5.31	91	5.55	142	5.59	52	4.99
Haematological	137	3.83	114	3.69	23	4.69	75	3.86	62	3.78	95	3.74	42	4.03
Other	1519	42.42	1325	42.87	194	39.59	851	43.84	668	40.73	1116	43.95	403	38.68

ED: emergency department; QoF: quality of outcome framework; COPD: chronic obstructive pulmonary disease; SD: standard deviation.

For peer review only - http://bmjopen.bmj.com/site/about/guidelines.xhtml

Sociodemographic and illness-related factors

Compared to people who did not experience the three measures, people with three or more hospital admissions in the last 90 days, admissions in the last 30 days and ED visits in the last two weeks were younger, more frequently male and lived in more deprived areas (Table 1).

More people with lung, prostate and hematological cancer experienced these measures. While it was not possible to observe a clear trend in terms of the number of comorbidities, people with a diagnosis of dementia, chronic obstructive pulmonary disease (COPD) and chronic heart disease had a higher proportion of three or more hospital admissions in the last 90 days, one or more admissions in the last 30 days and ED visits in the last two weeks respectively (Table 1).

Primary care and other community services

On average, people in the cohort had 2.3 (SD 3.3) telephone and 2.4 (SD 3.3) face-to-face consultations with the primary care practice, 8.6 (SD 13.7) contacts with community nurses, 1.2 (SD 3.0) contacts with community palliative care teams and 0.3 (SD 1.2) contacts with rehabilitation services in the last 90 days of life.

People with more contacts in the primary care practice were more likely to have three or more hospital admissions in the last 90 days and ED visits in the last two weeks of life. Conversely, people with more contacts with community nurses and palliative care teams were less likely to have hospital admissions in the last 30 days and ED visits in the last two weeks of life had. People living in care homes were less likely to experience admissions to hospital and ED visits (Table 2).

For peer review only

For peer review only - http://bmjopen.bmj.com/site/about/guidelines.xhtml

Table 2. Health care services utilization in the last three months of life by measure.

	Three or more hospital admissions in last 90 days						One or more hospital admission in last 30 days						One or more ED visit in last two weeks					
	No		Yes				No		Yes				No		Yes			
	n=3091		n=490				n=1941		n=1640				n=2539		n=1042			
	No.	%	No.	%	RR	95% CI	No.	%	No.	%	RR	95% CI	No.	%	No.	%	RR	95% CI
Contacts with primary care practice																		
0 to 3	1812	58.62	243	49.59	Ref		1144	58.94	911	55.55	Ref		1499	59.04	556	53.36	Ref	
4 to 10	873	28.24	149	30.41	1.21	(1.01 to 1.46)	539	27.77	483	29.45	1.05	(0.97 to 1.13)	705	27.77	317	30.42	1.12	(1.00 to 1.24)
>=11	406	13.13	98	20.00	1.59	(1.29 to 1.95)	258	13.29	246	15.00	1.08	(0.98 to 1.19)	355	13.98	169	16.22	1.19	(1.03 to 1.38)
Contacts with community nurses																		
0 to 3	1629	52.70	244	49.80	Ref		995	51.26	878	53.54	Ref		1300	51.20	573	54.99	Ref	
4 to 12	712	23.03	123	25.10	1.08	(0.90 to 1.29)	420	21.64	415	25.30	1.04	(0.96 to 1.13)	582	22.92	253	24.28	0.97	(0.86 to 1.10)
>=13	725	23.46	117	23.88	1.01	(0.83 to 1.24)	504	25.97	338	20.61	0.84	(0.76 to 0.93)	632	24.89	210	20.15	0.8	(0.69 to 0.93)
Contacts with community palliative care teams																		
0 to 3	2652	85.80	432	88.16	Ref		1644	84.70	1440	87.80	Ref		2170	85.47	914	87.72	Ref	
4 to 8	313	10.13	40	8.16	0.88	(0.68 to 1.15)	206	10.61	147	8.96	0.91	(0.80 to 1.04)	254	10.00	99	9.50	0.97	(0.82 to 1.14)
>=9	126	4.08	18	3.67	0.93	(0.62 to 1.37)	91	4.69	53	3.23	0.78	(0.63 to 0.96)	115	4.53	29	2.78	0.7	(0.50 to 0.97)
Contacts with rehabilitation teams																		
0	2830	91.56	450	91.84	Ref		1760	90.67	1520	92.68	Ref		2321	91.41	959	92.03	Ref	
1 to 3	186	6.02	29	5.92	1.01	(0.73 to 1.38)	127	6.54	88	5.37	0.89	(0.76 to 1.05)	153	6.03	62	5.95	1.01	(0.82 to 1.25)
>=4	75	2.43	11	2.24	0.93	(0.55 to 1.58)	54	2.78	32	1.95	0.8	(0.61 to 1.04)	65	2.56	21	2.02	0.81	(0.57 to 1.15)
Lived in a care home																		
No	2910	94.14	479	97.76	Ref		1797	92.58	1592	97.07	Ref		2387	94.01	1002	96.16	Ref	
Yes	181	5.86	11	2.24	0.43	(0.24 to 0.78)	144	7.42	48	2.93	0.54	(0.41 to 0.72)	152	5.99	40	3.84	0.71	(0.51 to 0.99)
						p-value						p-value						p-value
Days in hospital																		
Mean (SD)	11.85	(14.15)	24.98	(13.32)			10.21	(14.90)	17.71	(13.48)			13.10	(15.21)	14.98	(13.46)		
Median (IQR)	7.00	(0.0-19.0)	23.00	(15.0-32.0)		<0.001	2.00	(0.0-16.0)	15.00	(8.0-25.0)		<0.001	8.00	(0.0-20.0)	11.00	(5.0-21.0)		<0.001

For peer review only

For peer review only - http://bmjopen.bmj.com/site/about/guidelines.xhtml

Multivariate analysis

In the multivariate analysis, people with three or more hospital admissions in the last 90 days were more likely to be younger, have lung or prostate cancer, have more than 11 contacts with the primary care practice in the last 90 days and were less likely to live in a care home. People with one or more admissions in the last 30 days, were more likely to be younger, male, have breast cancer, they were less likely to live in a care home, and less likely to have more than 13 contacts with community nurses in the last 90 days. Having one or more ED visit in the last two weeks of life was associated with being younger, having fewer contacts with community nurses and lower chances of living in a care home (Table 3).

The sensitivity analysis demonstrated similar results, except for one or more admissions in the last 30 days, where the number of contacts with community nurses analysed as a continuous variable is no longer associated with the outcome measure, suggesting a threshold response (Supplemental tables S1, S2 and S3).

For peer review only

For peer review only - http://bmjopen.bmj.com/site/about/guidelines.xhtml

Table 3. Association between sociodemographic, illness-related and service-related factors with three measures for acute end-of-life care in the last three months of life.

		Three or more hospital admissions in last 3 months n=3,472		One or more hospital admissions in last month n=3,441		One or more ED last two weeks n=3,441	
		RR	95% CI	RR	95% CI	RR	95% CI
Age		**0.98**	**(0.97 to 0.99)**	**0.99**	**(0.99 to 0.99)**	**0.99**	**(0.99 to 0.99)**
Gender							
	Female	Ref		Ref		Ref	
	Male	1.10	(0.92 to 1.31)	**1.11**	**(1.02 to 1.20)**	1.10	(0.98 to 1.23)
IMD quintile							
	1	Ref		Ref		Ref	
	2	0.82	(0.65 to 1.03)	1.01	(0.91 to 1.12)	1.01	(0.89 to 1.15)
	3	0.88	(0.70 to 1.12)	1.03	(0.92 to 1.15)	0.93	(0.80 to 1.15)
	4	0.96	(0.75 to 1.24)	0.96	(0.85 to 1.09)	1.00	(0.84 to 1.18)
	5	0.95	(0.68 to 1.31)	1.06	(0.90 to 1.25)	0.98	(0.79 to 1.22)
Lived in care home (yes vs no)		**0.53**	**(0.28 to 0.98)**	**0.54**	**(0.41 to 0.72)**	**0.70**	**(0.49 to 0.99)**
Type of cancer							
	Bowel	Ref		Ref		Ref	
	Lung	**1.60**	**(1.16 to 2.20)**	1.08	(0.96 to 2.23)	1.01	(0.85 to 1.19)
	Prostate	**1.57**	**(1.07 to 2.30)**	0.98	(0.84 to 2.15)	0.89	(0.72 to 1.10)
	Breast	1.24	(0.82 to 1.87)	**1.19**	**(1.02 to 1.39)**	1.16	(0.94 to 1.42)
	Pancreas	1.23	(0.93 to 2.08)	1.02	(0.87 to 2.20)	0.82	(0.63 to 1.06)
	Haematological	1.23	(0.73 to 2.07)	0.91	(0.72 to 2.15)	0.93	(0.68 to 1.26)
	Other	1.15	(0.83 to 1.58)	0.96	(0.86 to 1.08)	0.82	(0.70 to 0.96)
Number QoF comorbidities							
	0	Ref		Ref		Ref	
	1	0.88	(0.69 to 1.11)	0.92	(0.83 to 1.02)	0.88	(0.75 to 1.04)
	2	0.99	(0.76 to 1.30)	1.06	(0.95 to 1.18)	1.05	(0.89 to 1.25)
	3	1.23	(0.94 to 1.62)	0.99	(0.87 to 1.12)	1.01	(0.84 to 1.21)
	>=4	0.98	(0.74 to 1.30)	1.11	(0.97 to 1.27)	1.09	(0.89 to 1.32)
	Dementia (yes vs no)	0.78	(0.54 to 1.14)	0.94	(0.82 to 1.07)		
	COPD (yes vs no)			1.07	(0.97 to 1.19)		
	Chronic heart disease (yes vs no)					1.14	(0.99 to 1.31)
Contacts with GP practice							
	0 to 3	Ref				Ref	
	4 to 10	1.18	(0.98 to 1.41)			1.10	(0.98 to 1.22)
	>=11	**1.63**	**(1.33 to 1.99)**			**1.27**	**(1.10 to 1.47)**
Contacts with community nurses							
	0 to 3			Ref		Ref	
	4 to 12			1.06	(0.98 to 1.15)	0.96	(0.85 to 1.08)
	>=13			**0.88**	**(0.90 to 0.98)**	**0.79**	**(0.68 to 0.92)**
Contacts with community palliative care teams							
	0 to 3			Ref		Ref	
	4 to 8			0.95	(0.82 to 1.08)	1.01	(0.85 to 1.21)
	>=9			0.85	(0.69 to 1.04)	0.78	(0.56 to 1.08)
Days in hospital		1.04	(1.03 to 1.04)	1.02	(1.01 to 1.02)	1.00	(1.00 to 1.01)

For peer review only - http://bmjopen.bmj.com/site/about/guidelines.xhtml

Discussion

The three measures for end-of-life cancer care evaluated (three or more admissions to hospital in the last 90 days, one or more hospital admission in the last 30 days and one or more ED visit in the last two weeks of life) were frequent (13.7%, 45.8% and 28.6% respectively). We found that being older and living in care homes were consistently associated with lower risk of having all three measures. Contacts with community nurses were associated with fewer ED visits in the last two weeks of life, and contacts with the primary care practice were associated with higher risk of multiple admissions to hospital in the last 90 days and ED visits in the last two weeks of life.

We found contacts with community nurses and living in care homes were associated with a lower risk of hospital admissions and ED visits at the end of life. These findings are consistent with previous studies where more community nursing hours per week were associated with lower odds of hospital admissions and ED visits at the end of life among patients with cancer in Canada.[19, 20] International studies show that people living in long-term facilities experience higher quality of care and comfort in the last weeks of life,[21] and are less likely to have transitions to hospital regardless of the cause of death.[22, 23] Long-term facilities are one of the very few care settings in the community providing continuous care including nights and weekends, which could potentially prevent the use of out-of-hours and acute care services.[24, 25] Community nurses have an important role providing physical care, managing symptoms and medications, educating and giving information to patients and families, and coordinating care,[26-29] and therefore they could play an important role in avoiding unnecessary hospital admissions towards the end of life in this population.

In contrast with previous studies,[19, 30] we found that contacts with the primary care practice were associated with higher risk of multiple admissions to hospital in the last 90 days and ED visits in the last two weeks of life. This is likely to be explained by complexity of health care needs: more severe and complex patients are likely to have a higher use of healthcare services.[31] A high number of contacts with the practice could be an opportunity to identify patients who are deteriorating or whose health care needs are increasing. High healthcare use can also be an indicator of unmet needs, ineffective or uncoordinated care, and lead to poor patient satisfaction.[32-34] It is possible that more contacts with the primary care practice in this sample reflects poor coordination or a lack of continuity of care, leading to more admissions to hospital.

Like other studies,[10, 35] we found that people with lung and prostate cancer were more likely to have multiple admissions in the last 90 days of life. These associations could be explained by differences in the prevalence of symptoms among different cancer types,[36] or higher rates of anti-cancer treatment at the end of life.[37] These consistent findings across studies suggest healthcare services should target these

For peer review only - http://bmjopen.bmj.com/site/about/guidelines.xhtml

group of patients for additional support when approaching the end of life, as improving the healthcare provision and support in the community might help prevent some hospital admissions in this population.

Implications for research and/or practice

Primary care physicians play a key role in providing care for people approaching the end of life. Their involvement is valued by patients and families,[38] and has shown to improve end-of-life care outcomes.[39, 40] However, several barriers to palliative care in general practice have been identified, such as the increasing workload and time constrains, lack of funding, poor communication with specialists and lack of experience and training.[41, 42] More research is needed to explore effective models of end-of-life care in primary care and palliative care integration in order to address the increasing demand for care and complexity of health care needs that patients experience when approaching the end of life.

The three measures used in this study have been proposed as quality indicators for cancer end-of-life care.[3, 43] Measuring the quality of care provided by health care services is key to monitor and promote the delivery of high-quality cancer care. We found an overlap between these measures, with 29.9% patients having more than one and different predictors associated with each of them, which suggests a combination of quality indicators might be needed to measure the quality of care provided by health care services. Although the measures chosen are recognised quality indicators, they only represent one component of quality at a population level and should be considered alongside other measures of quality such as patient experience and patient reported outcome measures (PROMs).

Strengths and limitations

The Discover dataset holds comprehensive information on healthcare services use from eight boroughs in London and over 2 million people, including information on primary, community and hospital care. However, our cohort is limited to a London population, which could limit the generalizability of the results.

Information on cause of death was not available, meaning some of the sample might have not died directly from cancer but from other conditions. We derived the date of death from primary care and hospital records, therefore some level of inaccuracy might be expected.[44] Primary care practice contacts were derived from Read codes and therefore it is possible the number of consultations with the practice was underestimated.[17, 18] We excluded administrative contacts and same day records with the primary care practice, as it has been done in other studies. This approach might underestimate the overall contribution of primary care practices in this study.

For peer review only - http://bmjopen.bmj.com/site/about/guidelines.xhtml

Restricting the sample to people who had been identified as having palliative care needs could have biased the sample toward people with higher or more complex health care needs and excluded people with palliative care needs that were not identified or recorded. It is likely that some palliative care services were not fully identified, as community palliative care is often provided by the voluntary sector in England, and therefore not consistently included in administrative records. This might explain the lack of association between community palliative care and measures of acute care use at the end of life found in our study.

We did not have information on the quality of care, continuity, coordination of care or the appropriateness of hospital admissions. Likewise, it was not possible to determine the length of stay or how close to death a person was admitted to the care home facility. These factors could also have an impact on the outcomes of this study.

Conclusions

In this population-based cohort study of people with cancer, multiple hospital admissions in the last 90 days and 30 days of life as well as multiple ED visits in the last two weeks of life were frequent. Living in a care home, and contacts with community nursing, were associated with fewer hospital admissions in the last 30 days of life and fewer ED visits in the last two weeks of life. To reduce acute hospital use at the end of life and improve quality of care, policymakers should consider expanding community nursing and care home services. Further research exploring the most effective models of palliative care are needed.

References

1. Global, regional, and national age-sex-specific mortality for 282 causes of death in 195 countries and territories, 1980-2017: a systematic analysis for the Global Burden of Disease Study 2017. *Lancet* 2018; 392: 1736-1788. 2018/11/30. DOI: 10.1016/s0140-6736(18)32203-7.
2. Smittenaar CR, Petersen KA, Stewart K, et al. Cancer incidence and mortality projections in the UK until 2035. *Br J Cancer* 2016; 115: 1147-1155. 2016/10/26. DOI: 10.1038/bjc.2016.304.
3. Henson LA, Edmonds P, Johnston A, et al. Population-Based Quality Indicators for End-of-Life Cancer Care: A Systematic Review. *JAMA Oncol* 2019 2019/10/28. DOI: 10.1001/jamaoncol.2019.3388.
4. Ersek M, Miller SC, Wagner TH, et al. Association between aggressive care and bereaved families' evaluation of end-of-life care for veterans with non-small cell lung cancer who died in Veterans Affairs facilities. *Cancer* 2017; 123: 3186-3194. 2017/04/19. DOI: 10.1002/cncr.30700.

For peer review only - http://bmjopen.bmj.com/site/about/guidelines.xhtml

5. Wright AA, Keating NL, Ayanian JZ, et al. Family Perspectives on Aggressive Cancer Care Near the End of Life. *Jama* 2016; 315: 284-292. 2016/01/20. DOI: 10.1001/jama.2015.18604.

6. Wright AA, Zhang B, Keating NL, et al. Associations between palliative chemotherapy and adult cancer patients' end of life care and place of death: prospective cohort study. *Bmj* 2014; 348: g1219. 2014/03/07. DOI: 10.1136/bmj.g1219.

7. Golan R, Bernstein AN, Gu X, et al. Increased resource use in men with metastatic prostate cancer does not result in improved survival or quality of care at the end of life. *Cancer* 2018; 124: 2212-2219. 2018/03/27. DOI: 10.1002/cncr.31297.

8. Temel JS, Greer JA, Muzikansky A, et al. Early palliative care for patients with metastatic non-small-cell lung cancer. *N Engl J Med* 2010; 363: 733-742. 2010/09/08. DOI: 10.1056/NEJMoa1000678.

9. Earle CC, Landrum MB, Souza JM, et al. Aggressiveness of cancer care near the end of life: is it a quality-of-care issue? *Journal of clinical oncology : official journal of the American Society of Clinical Oncology* 2008; 26: 3860-3866. DOI: 10.1200/JCO.2007.15.8253.

10. Henson LA, Gao W, Higginson IJ, et al. Emergency department attendance by patients with cancer in their last month of life: a systematic review and meta-analysis. *J Clin Oncol* 2015; 33: 370-376. 2014/12/24. DOI: 10.1200/jco.2014.57.3568.

11. Koroukian SM, Schiltz NK, Warner DF, et al. Social determinants, multimorbidity, and patterns of end-of-life care in older adults dying from cancer. *J Geriatr Oncol* 2017; 8: 117-124. 2016/12/29. DOI: 10.1016/j.jgo.2016.10.001.

12. Mercadante S, Masedu F, Valenti M, et al. The characteristics of advanced cancer patients followed at home, but admitted to the hospital for the last days of life. *Intern Emerg Med* 2016; 11: 713-718. 2016/02/20. DOI: 10.1007/s11739-016-1402-1.

13. Abedini NC, Hechtman RK, Singh AD, et al. Interventions to reduce aggressive care at end of life among patients with cancer: a systematic review. *Lancet Oncol* 2019; 20: e627-e636. 2019/11/02. DOI: 10.1016/s1470-2045(19)30496-6.

14. Bottle A, Cohen C, Lucas A, et al. How an electronic health record became a real-world research resource: comparison between London's Whole Systems Integrated Care database and the Clinical Practice Research Datalink. *BMC Medical Informatics and Decision Making* 2020; 20: 71. DOI: 10.1186/s12911-020-1082-7.

15. NHS Digital. Business Rules for Quality and Outcomes Framework (QOF) 2017/2018, https://digital.nhs.uk/data-and-information/data-collections-and-data-sets/data-collections/quality-and-outcomes-framework-qof (2017).

For peer review only

For peer review only - http://bmjopen.bmj.com/site/about/guidelines.xhtml

16. NHS England. CCG improvement and assessment framework 2017/18, https://www.england.nhs.uk/wp-content/uploads/2017/11/ccg-improvement-and-assessment-framework-2017-18.pdf (2017, accessed September 2020).

17. Kontopantelis E, Olier I, Planner C, et al. Primary care consultation rates among people with and without severe mental illness: a UK cohort study using the Clinical Practice Research Datalink. *BMJ Open* 2015; 5: e008650. 2015/12/18. DOI: 10.1136/bmjopen-2015-008650.

18. Browne J, Edwards DA, Rhodes KM, et al. Association of comorbidity and health service usage among patients with dementia in the UK: a population-based study. *BMJ Open* 2017; 7: e012546. 2017/03/11. DOI: 10.1136/bmjopen-2016-012546.

19. Almaawiy U, Pond GR, Sussman J, et al. Are family physician visits and continuity of care associated with acute care use at end-of-life? A population-based cohort study of homecare cancer patients. *Palliat Med* 2014; 28: 176-183. 2013/06/20. DOI: 10.1177/0269216313493125.

20. Seow H, Barbera L, Howell D, et al. Using more end-of-life homecare services is associated with using fewer acute care services: a population-based cohort study. *Med Care* 2010; 48: 118-124. 2010/01/09. DOI: 10.1097/MLR.0b013e3181c162ef.

21. Collingridge Moore D, Payne S, Keegan T, et al. Associations between Length of Stay in Long Term Care Facilities and End of Life Care. Analysis of the PACE Cross-Sectional Study. *Int J Environ Res Public Health* 2020; 17 2020/04/23. DOI: 10.3390/ijerph17082742.

22. Pivodic L, Pardon K, Miccinesi G, et al. Hospitalisations at the end of life in four European countries: a population-based study via epidemiological surveillance networks. *J Epidemiol Community Health* 2016; 70: 430-436. 2015/11/21. DOI: 10.1136/jech-2015-206073.

23. Van den Block L, Pivodic L, Pardon K, et al. Transitions between health care settings in the final three months of life in four EU countries. *Eur J Public Health* 2015; 25: 569-575. 2015/04/02. DOI: 10.1093/eurpub/ckv039.

24. Hirakawa Y, Kuzuya M and Uemura K. Opinion survey of nursing or caring staff at long-term care facilities about end-of-life care provision and staff education. *Arch Gerontol Geriatr* 2009; 49: 43-48. 2008/06/10. DOI: 10.1016/j.archger.2008.04.010.

25. Zimmerman S, Sloane PD, Hanson L, et al. Staff perceptions of end-of-life care in long-term care. *J Am Med Dir Assoc* 2003; 4: 23-26. 2003/06/17. DOI: 10.1097/01.Jam.0000046935.64053.54.

26. Boot M. Exploring the district nurse role in facilitating individualised advance care planning. *Br J Community Nurs* 2016; 21: 144-147. 2016/03/05. DOI: 10.12968/bjcn.2016.21.3.144.

27. Latter S, Hopkinson JB, Lowson E, et al. Supporting carers to manage pain medication in cancer patients at the end of life: A feasibility trial. *Palliat Med* 2018; 32: 246-256. 2017/07/06. DOI: 10.1177/0269216317715197.

For peer review only

28. Offen J. The role of UK district nurses in providing care for adult patients with a terminal diagnosis: a meta-ethnography. *Int J Palliat Nurs* 2015; 21: 134-141. 2015/03/31. DOI: 10.12968/ijpn.2015.21.3.134.

29. Walshe C and Luker KA. District nurses' role in palliative care provision: a realist review. *Int J Nurs Stud* 2010; 47: 1167-1183. 2010/05/25. DOI: 10.1016/j.ijnurstu.2010.04.006.

30. Kronman AC, Ash AS, Freund KM, et al. Can primary care visits reduce hospital utilization among Medicare beneficiaries at the end of life? *J Gen Intern Med* 2008; 23: 1330-1335. 2008/05/29. DOI: 10.1007/s11606-008-0638-5.

31. Bone AE, Evans CJ, Henson LA, et al. Patterns of emergency department attendance among older people in the last three months of life and factors associated with frequent attendance: a mortality follow-back survey. *Age Ageing* 2019; 48: 680-687. 2019/05/28. DOI: 10.1093/ageing/afz043.

32. Blumenthal D and Abrams MK. Tailoring Complex Care Management for High-Need, High-Cost Patients. *Jama* 2016; 316: 1657-1658. 2016/10/27. DOI: 10.1001/jama.2016.12388.

33. Salzberg CA, Hayes SL, McCarthy D, et al. Health System Performance for the High-Need Patient: A Look at Access to Care and Patient Care Experiences. *Issue Brief (Commonw Fund)* 2016; 27: 1-12. 2016/08/31.

34. Wennberg JE, Bronner K, Skinner JS, et al. Inpatient care intensity and patients' ratings of their hospital experiences. *Health Aff (Millwood)* 2009; 28: 103-112. 2009/01/07. DOI: 10.1377/hlthaff.28.1.103.

35. Henson LA, Gomes B, Koffman J, et al. Factors associated with aggressive end of life cancer care. *Support Care Cancer* 2016; 24: 1079-1089. 2015/08/09. DOI: 10.1007/s00520-015-2885-4.

36. van den Beuken-van Everdingen MH, Hochstenbach LM, Joosten EA, et al. Update on Prevalence of Pain in Patients With Cancer: Systematic Review and Meta-Analysis. *J Pain Symptom Manage* 2016; 51: 1070-1090.e1079. 2016/04/27. DOI: 10.1016/j.jpainsymman.2015.12.340.

37. Bylicki O, Rivière F, Tournier C, et al. Factors Associated With Aggressiveness of End-of-Life Care for Lung Cancer Patients and Associated Costs of Care. *Clin Lung Cancer* 2020 2020/07/11. DOI: 10.1016/j.cllc.2020.05.017.

38. Green E, Knight S, Gott M, et al. Patients' and carers' perspectives of palliative care in general practice: A systematic review with narrative synthesis. *Palliat Med* 2018; 32: 838-850. 2018/01/19. DOI: 10.1177/0269216317748862.

39. Kim SL and Tarn DM. Effect of Primary Care Involvement on End-of-Life Care Outcomes: A Systematic Review. *J Am Geriatr Soc* 2016; 64: 1968-1974. 2016/10/21. DOI: 10.1111/jgs.14315.

For peer review only - http://bmjopen.bmj.com/site/about/guidelines.xhtml

40. Mitchell G, Aubin M, Senior H, et al. General practice nurses and physicians and end of life: a systematic review of models of care. *BMJ Support Palliat Care* 2020 2020/07/29. DOI: 10.1136/bmjspcare-2019-002114.
41. Carmont SA, Mitchell G, Senior H, et al. Systematic review of the effectiveness, barriers and facilitators to general practitioner engagement with specialist secondary services in integrated palliative care. *BMJ Support Palliat Care* 2018; 8: 385-399. 2017/02/16. DOI: 10.1136/bmjspcare-2016-001125.
42. Mitchell S, Loew J, Millington-Sanders C, et al. Providing end-of-life care in general practice: findings of a national GP questionnaire survey. *Br J Gen Pract* 2016; 66: e647-653. 2016/07/07. DOI: 10.3399/bjgp16X686113.
43. De Schreye R, Houttekier D, Deliens L, et al. Developing indicators of appropriate and inappropriate end-of-life care in people with Alzheimer's disease, cancer or chronic obstructive pulmonary disease for population-level administrative databases: A RAND/UCLA appropriateness study. *Palliat Med* 2017; 31: 932-945. 2017/04/22. DOI: 10.1177/0269216317705099.
44. Gallagher AM, Dedman D, Padmanabhan S, et al. The accuracy of date of death recording in the Clinical Practice Research Datalink GOLD database in England compared with the Office for National Statistics death registrations. *Pharmacoepidemiology and Drug Safety* 2019; 28: 563-569. DOI: 10.1002/pds.4747.

Additional information

Funding statement

J.L is funded by a Royal Marsden Partners Pan London Research Fellowship Award (Award number not applicable) and the Programa Formacion de Capital Humano Avanzado, Doctorado Becas Chile, 2018 (folio 72190265). KES is funded by a National Institute of Health Research (NIHR) Clinician Scientist Fellowship (CS-2015-15-005) and is the Laing Galazka Chair in Palliative Care at King's College London, funded by an endowment from Cicely Saunders International and the Kirby Laing Foundation. IJH is an NIHR Senior Investigator Emeritus. IJH is supported by the NIHR Applied Research Collaboration South London (NIHR ARC South London) at King's College Hospital NHS Foundation Trust. IJH leads the Palliative and End of Life Care theme of the NIHR ARC South London, and co-leads the national theme in this. The views expressed are those of the authors and not necessarily those of the NHS, the NIHR, the Department of Health and Social Care or the funding charities.

Ethical approval

For peer review only

The source database is approved for secondary analysis by the West Midland-Solihull Research Ethic Committee (reference 18/WM/0323). All data was anonymised and therefore, no patient consent was required.

Competing interests

The Authors declare that there is no conflict of interest.

Authors' contribution

JL and KES had the idea for the study. JL designed the study with input from KES, ZU and IJH. Data analysis was carried out by JL with input from KES, GW, LH, ZU and IJH. All authors helped interpret the data. JL wrote the first draft of the paper. All authors contributed to subsequent drafts and approved the final paper.

Data sharing statement

The data that support the findings of this study are available from the Discover dataset but restrictions apply to the availability of these data, which were used under license for the current study, and so are not publicly available.

For peer review only

For peer review only - http://bmjopen.bmj.com/site/about/guidelines.xhtml

Supplementary material

Figure S1. Flowchart of the data management

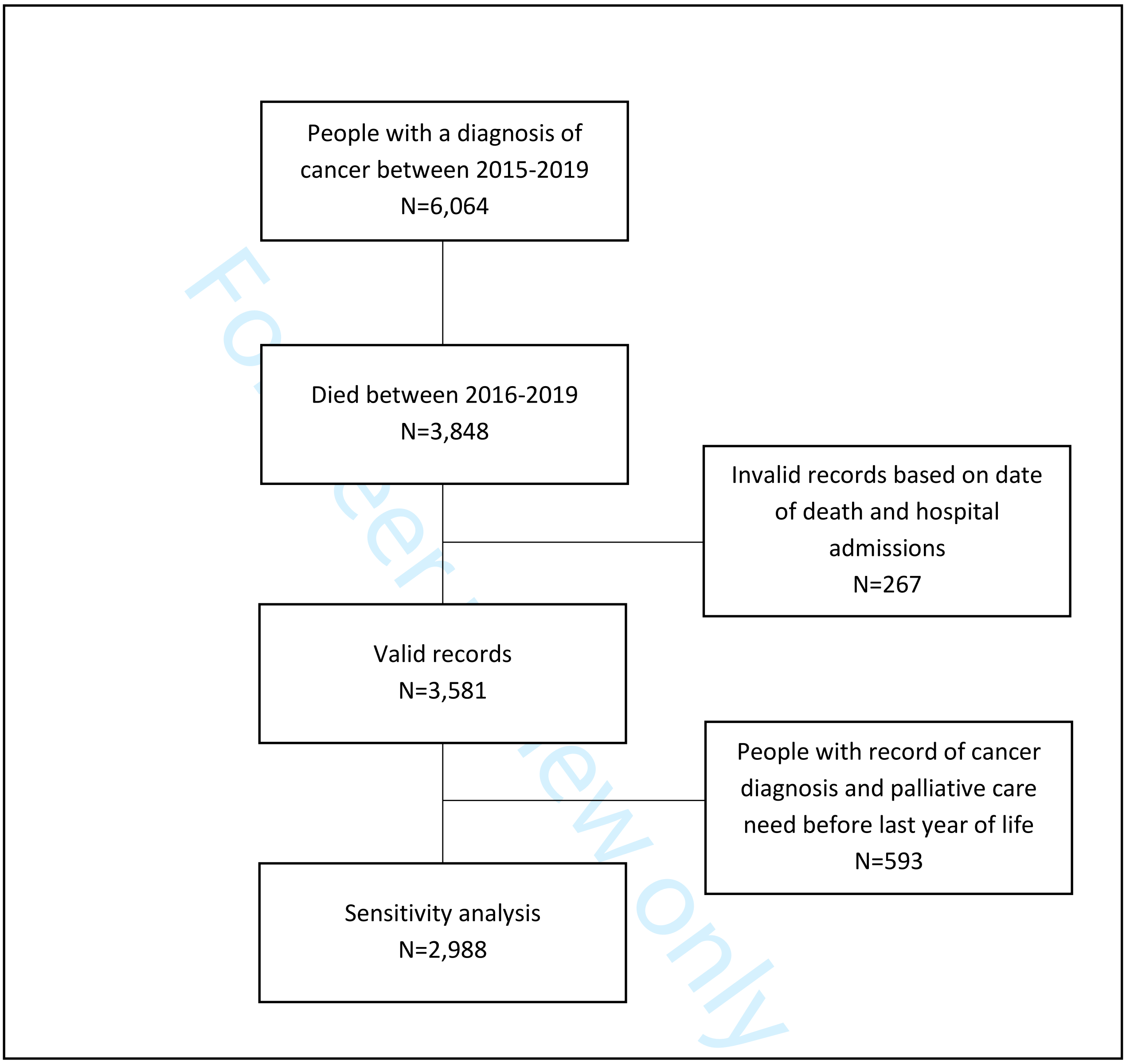

Box S1. List of Read codes and ICD-10 codes used to identify the cohort and variables.

	Read Codes v2	ICD10 codes
Diagnosis of cancer	B0%, B1%, B2%, B3%, B4%, B5%, B6%, Byu%, K1323, K01w1, 68W24, C184., NOT(B677.)	C00 to C97
Type of cancer		
Lung cancer	B22%	C34.0 to C34.9
Bowel cancer	B12% to B14%	C17.0 to C21.8
Prostate cancer	B45%	C61
Breast cancer	B34% to B35%	C50.0 to C50.9
Pancreas cancer	B17%	C25.0 to C25.9
Haematological cancer	B61% to B62% and B64% to B69%	C81.0 to C86.6 and C91.0 to C95.9
Date of death	22J.., 9491., 9495., 94G.., 8HG.., 9493., 94E.., 946.., 94Z.., ZV680, 94..., 949A., 949.., 9431., 9442., 9451., 9452., 9453., 946.., 9492., 9494., 9496., 9497., 9498., 9499., 949B., 949C., 949D., 949E., 949F., 949G., 949H., 949J., 949Z., 94D.., 94G..	
Palliative care QoF	1Z01. , 2JE.. , 2Jf.. , 38VY. , 38Vb. , 38Vd. , 38Ve. , 38Vf. , 38Vg. , 38Vh. , 38Vi. , 8BA2. , 8BAP. , 8BAS. , 8BAT. , 8BAe. , 8BJ1. , 8CM1.% (NOT 8CM15) , 8CM4. , 8CME. , 8CMj. , 8CMk. , 8H6A. , 8H7L. , 8H7g. , 8HH7. , 8IEE. , 9EB5. , 9Ng7. , ZV57C , 8CMQ. , 9NgD. , 9G8.. , 9c0P. , 9c0N. , 8CMW3 , 9K9.. , 9367. , 9c0L0 , 9c0M. , 9NNd. , 8CMb. , 8B2a. , 9NNf0 , 38QH. , 38QK. , 8CMg. , 2Jg.., 9NNq. , 9NNr. , 9NNs.	
Quality of Outcomes Framework (QoF) Rules		
Asthma	H33%, H3120, H3B..,173A., NOT (H333., 21262, 212G.)	
Atrial Fibrillation	G573% NOT (212R.)	
Hypertension	G2..%, G20%, Gyu2., Gyu20, G24..-G2z.., NOT (G24z1, G2400, G2410, G27..)	
Diabetes	C10.., C109J, C109K, C10C., C10D., PKyP., C10Q., C10E%, C10F%, C10H%, C10M%, C10N%, C10P% NOT(C10F8)	
Congestive heart disease	G3...-G309., G30B.-G330z, G33z.-G3401, G342.-G35X., G38..-G3z.., Gyu3%, NOT(Gyu31, G310.)	
Chronic Kidney disease	1Z12., 1Z13. , 1Z14. , 1Z15. , 1Z16. , 1Z1B.-1Z1L. , K053. , K054. , K055. , 1Z1T. , 1Z1V. , 1Z1W. , 1Z1X. , 1Z1Y. , 1Z1Z. , 1Z1a. , 1Z1b. , 1Z1c. , 1Z1d. , 1Z1e. , 1Z1f., 1Z10. , 1Z11. , 1Z17.-1Z1A., K051., K052., 1Z1M., 1Z1Q., 1Z1N., 1Z1P., 1Z1R., 1Z1S. NOT(2126E)	
COPD	H5832, H4640, H4641, Hyu30, Hyu31, H3..%, H31%, H32%, H36..-H3z.., NOT(H3101, H31y0, H3122, H3y0., H3y1.	
Depression	E0013, E0021, E118., E11y2, E11z2, E130, E135., E2003, E291., E2B.., E2B1., Eu204, Eu251, Eu341, Eu412, E112%, E113%, Eu32%, Eu33%, NOT(Eu32a, Eu32B, Eu329, 212S.)	
Epilepsy	F1321, SC200, F25%, NOT(F2501, F2504, F2511, F2516, F25y4, F25G., F25H., 21260, 212J, F256%, F258.-F25A.)	
Heart Failure	G58%, G1yz1, 662f.-662i.	
Peripheral Arterial Disease	G73.., Gyu74, G734., G73y., G73z%, NOT(G73z1)	

For peer review only

Rheumatoid Arthritis	N041., N047., N04X., N04y0, N04y2, Nyu11, Nyu12, Nyu1G, Nyu10, G5yA., G5y8., N040%, N042%, NOT(N0420)	
Stroke	G65..-G654., G656.-G65zz, G63y0.-G63y1,Gyu62-Gyu66, ZV12D, Fyu55, G6760, G6W.., G6X.., Gyu6F, Gyu6G, G61%, G64%, G66%, NOT(G617., G669.)	
Mental Health	E1124, E1134, E11z., E11z0, E11zz, E2122, Eu323, Eu328, Eu333, Eu32A, Eu329, E114.-E117z, E10%, E110%, E111%, E11y%, E12%, E13%, Eu2%, Eu30%, Eu31%, NOT(E11y2, E135.)	
Dementia	F110 to F112, E02y1, E041., Eu041, F116.,F118., F21y2, A410., Eu107, F11x7, Eu02%, E00%, Eu01%, E012%, Eu00%, A411%	

For peer review only

Box S2. List of codes used to derive primary care contacts

	**Read code v2**
Primary care practice face to face contacts	9N1C., 9N1w., 9NF7., 9NF8., 9k27., 9N1G., 9NFB., 9NFW., 9N1t., 9N1x., 9NF5., 9NF4., 9NF6., 982B., 982C., 9N11., 9N12., 9N1c., 9N1y0, 9N1z., 9c0H., 9N01., 9N0G., 9N7B., 9NV.., 9NY.., 9NY0.,
Primary care practice telephone contacts	9N31., 9b0m., 9b0n., 9b0o, 9N310, 9N310, 9N311, 9N3A, 8CAN., 8CAR0
Primary care practice failed contact	9N4.., 9N41%, 9Ni..

Box S3. List of codes used to derive contacts with other community care professionals

	**Service description**	**Service reporting line**
Community nurses contacts	District Nurse<br>District Nurse, Adult, Face to face<br>District Nurse, Adult, Non face to face<br>District Nurses<br>District Nursing Services: Adult<br>Cardiac Nursing / Liaison: Adult<br>Nurse<br>Nursing Services for Children<br>Other Specialist Nursing<br>Other Specialist Nursing, Adult, Face to face<br>Specialist Nursing - Asthma and Respiratory Nursing/Liaison<br>Specialist Nursing - Cardiac Nursing / Liaison<br>Specialist Nursing - Continence Services<br>Specialist Nursing - Diabetic Nursing / Liaison<br>Specialist Nursing - Parkinson''s and Alzheimers Nursing/Liaison<br>Specialist Nursing - Tissue Viability Nursing/Liaison<br>Specialist Nursing - Tuberculosis Specialist Nursing<br>Specialist Nursing, Active Case Management (Community Matrons)<br>Specialist Nursing, Asthma and Respiratory Nursing/Liaison, Adult, Face to face<br>Specialist Nursing, Asthma and Respiratory Nursing/Liaison, Adult, Non face to face<br>Specialist Nursing, Cardiac Nursing/Liaison, Adult, Face to face<br>Specialist Nursing, Cardiac Nursing/Liaison, Adult, Non face to face<br>Specialist Nursing, Continence Services<br>"Specialist Nursing, Continence Services, Adult, Face to face<br>Specialist Nursing, Continence Services, Adult, Non face to face<br>Specialist Nursing, Diabetic Nursing/Liaison<br>Specialist Nursing, Diabetic Nursing/Liaison, Adult, Face to face<br>Specialist Nursing, Diabetic Nursing/Liaison, Adult, Non face to face<br>Specialist Nursing, Parkinson''s and Alzheimers Nursing/Liaison<br>Specialist Nursing, Stoma Care Services, Adult, Face to face<br>Specialist Nursing, Tissue Viability Nursing/Liaison<br>Specialist Nursing, Tissue Viability Nursing/Liaison, Adult, Face to face<br>"Specialist Nursing, Tissue Viability Nursing/Liaison, Adult, Non face to face<br>Tissue Viability Nursing / Liaison: Adult<br>Tuberculosis Special Nursing: Adult	District Nursing<br>District Nursing (H&F, K&C, W)<br>District Nursing (H&F, K&C, W)<br>District Nursing – AWC<br>24 Hour Nursing<br>24 Hour Nursing (Phlebotomy)<br>Adult Nursing<br>CSCNS - Community Nursing<br>Children''s Community Nursing<br>Community Matron<br>Community Matron (H&F, K&C, W)<br>Community Matrons<br>Community Matrons (H&F K&C W)<br>Community Nursing<br>Heart Failure Nursing<br>Heart Nurses (K&C)<br>Night Nursing<br>Night Nursing (K&C)<br>TB Nursing<br>Tissue Viability Nursing<br>Twilight/Night Nursing Service

For peer review only

Palliative care community team contacts	Palliative / Respite Care: Adult<br>Specialist Nursing - Palliative / Respite Care<br>Specialist Nursing, Palliative/Respite Care, Adult, Face to face<br>Specialist Nursing, Palliative/Respite Care, Adult, Non face to face	Palliative Care<br>Palliative Care Service<br>Palliative Medicine (Consultant)<br>Pembridge Bereavement Counselling<br>Pembridge Community<br>Pembridge Day Care
Rehabilitation teams contacts	Community Rehabilitation Teams<br>Other Therapist, Adult, One to One<br>Physiotherapist<br>Physiotherapist, Adult, One to One<br>Physiotherapy<br>Physiotherapy Services: Adult<br>Rehabilitation for Other Disorders<br>Rehabilitation for Other Musculoskeletal Disorders<br>Rehabilitation for Other Neurological Disorders<br>Rehabilitation for Respiratory Disorders<br>SLT - Adult<br>Speech and Language Therapist, Adult, One to One<br>Speech and Language Therapy<br>Occupational Therapist, Adult, One to One<br>Occupational Therapy	Bedded Rehab – Therapists<br>Bedded Rehab - Therapists (H&F, K&C, W)<br>Brent Rehabilitation Service<br>Cardiac Rehabilitation<br>Claypond RehabTherapy<br>Community IFC MSK Physiotherapy Service<br>Community MSK Physiotherapy Service<br>Community Neuro Rehab<br>Community Neuro-Rehabilitation (H&F, K&C, W)<br>Community Recovery Service - Neuro Rehab<br>Community Rehab ICE<br>Community Rehabilitation<br>Community Rehabilitation (H&F, K&C, W)<br>EDTC - Community Physio<br>EHT Therapies<br>Ealing Hospital Therapies<br>Integrated Rehab<br>MSK Physiotherapy<br>Musculoskeletal Service<br>Musculoskeletal Service (W)<br>Physio (MSK)<br>Physiotherapy<br>Pulmonary Rehab<br>Short Term Rehabilitation<br>Therapies MS Physio<br>Adult SLT<br>SLT (Adults)<br>Occupational Therapy

For peer review only

Table S1. Sensitivity analysis for three or more hospital admissions in the last 90 days

	Model 1		Model 2		Model 3		Model 4		Model 5	
	Original Model n=3472		Model 1 without days in hospital n=3472		Model 1 including contacts with PC practice as continuous n=3472		Model 1 only for people with a record of cancer diagnosis and identification of palliative care needs in the last 12 months of life n=2703		Model 1 with ethnicity n-=2841	
	RR	95% CI	RR	95% CI	RR	95% CI	RR	95% CI	RR	95% CI
Age	0.98	(0.97 to 0.99)	0.98	(0.97 to 0.98)	0.98	(0.97 to 0.98)	0.98	(0.97 to 0.98)	0.98	(0.97 to 0.99)
Gender (Male vs female)	1.10	(0.92 to 1.31)	1.11	(0.92 to 1.34)	1.10	(0.92 to 1.02)	1.05	(0.87 to 1.27)	1.06	(0.88 to 1.28)
IMD quintile (Ref=1)										
2	0.82	(0.65 to 1.03)	0.87	(0.70 to 1.09)	0.81	(0.65 to 1.02)	0.87	(0.67 to 1.12)	0.83	(0.65 to 1.05)
3	0.89	(0.70 to 1.12)	0.87	(0.67 to 1.12)	0.88	(0.70 to 1.12)	0.87	(0.66 to 1.13)	0.83	(0.64 to 1.09)
4	0.96	(0.75 to 1.24)	0.90	(0.68 to 1.17)	0.97	(0.75 to 1.24)	1.01	(0.77 to 1.33)	0.97	(0.73 to 1.30)
5	0.95	(0.68 to 1.31)	0.80	(0.58 to 1.11)	0.93	(0.67 to 1.29)	0.90	(0.62 to 1.30)	0.85	(0.56 to 1.28)
Living in care home (Yes vs No)	0.53	(0.28 to 0.98)	0.54	(0.29 to 0.98)	0.52	(0.28 to 0.98)	0.54	(0.28 to 1.05)	0.64	(0.34 to 1.20)
Type of cancer (Ref=Bowel)										
Lung	1.60	(1.16 to 2.20)	1.51	(1.10 to 2.09)	1.61	(1.17 to 2.21)	1.68	(1.17 to 2.41)	1.63	(1.16 to 2.28)
Prostate	1.57	(1.07 to 2.30)	1.81	(1.22 to 2.67)	1.57	(1.07 to 2.30)	1.55	(1.01 to 2.38)	1.64	(1.08 to 2.51)
Breast	1.24	(0.82 to 1.87)	1.33	(0.86 to 2.03)	1.24	(0.82 to 1.87)	1.27	(0.77 to 2.08)	1.15	(0.71 to 1.84)
Pancreas	1.39	(0.93 to 2.08)	1.33	(0.87 to 2.05)	1.42	(0.95 to 2.13)	1.51	(0.96 to 2.37)	1.47	(0.95 to 2.28)
Haematological	1.23	(0.73 to 2.07)	1.82	(1.11 to 2.98)	1.23	(0.73 to 2.08)	1.19	(0.65 to 2.16)	1.13	(0.62 to 2.05)
Other	1.15	(0.83 to 1.58)	1.22	(0.87 to 1.69)	1.15	(0.84 to 1.58)	1.23	(0.86 to 1.78)	1.19	(0.84 to 1.69)
Number of QoF comorbidities (Ref=0)										
1	0.88	(0.69 to 1.11)	0.97	(0.75 to 1.25)	0.87	(0.68 to 1.10)	0.87	(0.67 to 1.13)	0.88	(0.66 to 1.17)
2	0.99	(0.76 to 1.30)	1.08	(0.81 to 1.44)	0.98	(0.75 to 1.29)	0.96	(0.71 to 1.29)	0.89	(0.65 to 1.20)
3	1.23	(0.94 to 1.62)	1.38	(1.04 to 1.83)	1.24	(0.94 to 1.63)	1.18	(0.87 to 1.60)	1.14	(0.84 to 1.55)
>=4	0.98	(0.74 to 1.30)	1.17	(0.88 to 1.57)	0.97	(0.74 to 1.28)	0.91	(0.66 to 1.25)	0.91	(0.66 to 1.26)
Dementia (Yes vs No)	0.78	(0.54 to 1.14)	0.77	(0.53 to 1.13)	0.79	(0.54 to 1.14)	0.80	(0.51 to 1.27)	0.76	(0.49 to 1.16)
Contacts with the primary care practice (Ref= 0 to 3)										
4 to 10	1.18	(0.98 to 1.41)	1.16	(0.97 to 1.39)			1.15	(0.94 to 1.39)	1.20	(0.98 to 1.47)
>=11	1.63	(1.33 to 1.99)	1.52	(1.23 to 1.88)			1.64	(1.32 to 2.04)	1.77	(1.41 to 2.22)
Number of contacts with primary care practice (continuous)					1.02	(1.01 to 1.03)				
Number of days in hospital in the last 90 days	1.04	(1.03 to 1.04)			1.04	(1.03 to 1.04)	1.04	(1.03 to 1.04)	1.04	(1.03 to 1.04)
Ethnicity (Ref= white)										
Black									1.11	(0.82 to 1.50)
Asian									1.52	(1.21 to 1.90)
Mixed									1.19	(0.93 to 1.51)
Other									1.08	(0.73 to 1.60)

Table S2. Sensitivity analysis for one or more hospital admissions in the last 30 days

	Model 1		Model 2		Model 3		Model 4		Model 5	
	Original Model		Model 1 without days in hospital		Model 1 including contacts with primary care practice as con-tinuous		Model 1 for people with a record of cancer diagnosis and identification of pallia-tive care needs in the last 12 months of life		Model 1 with ethnicity	
	n=3441		n=3441		n=3441		n=2679		n-=2815	
	RR	95% CI	RR	95% CI	RR	95% CI	RR	95% CI	RR	95% CI
Age	1.00	(0.99 to 0.99)	0.99	(0.99 to 1.00)	1.00	(0.99 to 1.00)	1.00	(0.99 to 1.00)	1.00	(0.99 to 1.00)
Gender (Male vs female)	1.11	(1.02 to 1.20)	1.11	(1.02 to 1.21)	1.11	(1.03 to 1.21)	1.10	(1.00 to 1.20)	1.08	(0.99 to 1.19)
IMD quintile (Ref=1)										
2	1.01	(0.91 to 1.12)	1.03	(0.92 to 1.14)	1.01	(0.91 to 1.12)	1.03	(0.92 to 1.16)	1.05	(0.93 to 1.18)
3	1.03	(0.92 to 1.15)	1.02	(0.91 to 1.15)	1.02	(0.92 to 1.14)	1.06	(0.93 to 1.19)	1.05	(0.92 to 1.17)
4	0.96	(0.85 to 1.09)	0.94	(0.82 to 1.07)	0.96	(0.85 to 1.08)	0.99	(0.86 to 1.13)	0.95	(0.82 to 1.09)
5	1.06	(0.90 to 1.25)	1.02	(0.86 to 1.20)	1.06	(0.90 to 1.26)	1.08	(0.91 to 1.28)	1.06	(0.86 to 1.29)
Living in care home (Yes vs no)	0.54	(0.41 to 0.72)	0.54	(0.40 to 0.71)	0.54	(0.41 to 0.73)	0.58	(0.43 to 0.79)	0.58	(0.44 to 0.80)
Type of cancer (Ref=Bowel)										
Lung	1.09	(0.96 to 2.23)	1.06	(0.93 to 1.21)	1.08	(0.95 to 1.23)	1.06	(0.91 to 1.23)	1.06	(0.92 to 1.22)
Prostate	0.98	(0.84 to 2.15)	1.04	(0.89 to 1.22)	0.98	(0.83 to 1.14)	0.94	(0.78 to 1.14)	1.05	(0.87 to 1.24)
Breast	1.19	(1.02 to 1.39)	1.21	(1.03 to 1.43)	1.18	(1.01 to 1.38)	1.22	(1.02 to 1.47)	1.17	(0.96 to 1.40)
Pancreas	1.02	(0.87 to 2.20)	1.01	(0.85 to 1.20)	1.03	(0.87 to 1.21)	1.05	(0.88 to 1.25)	1.07	(0.90 to 1.29)
Haematological	0.91	(0.72 to 2.15)	1.04	(0.83 to 1.31)	0.91	(0.71 to 1.15)	0.85	(0.65 to 1.11)	0.94	(0.73 to 1.21)
Other	0.96	(0.86 to 1.08)	0.98	(0.87 to 1.11)	0.96	(0.85 to 1.08)	0.94	(0.82 to 1.07)	1.00	(0.87 to 1.14)
Number of QoF comorbidities (Ref=0)										
1	0.92	(0.83 to 1.02)	0.96	(0.86 to 1.07)	0.92	(0.83 to 1.02)	0.93	(0.83 to 1.05)	0.93	(0.81 to 1.05)
2	1.06	(0.95 to 1.18)	1.10	(0.98 to 1.23)	1.06	(0.95 to 1.18)	1.12	(1.00 to 1.26)	1.02	(0.89 to 1.16)
3	0.99	(0.87 to 1.12)	1.03	(0.91 to 1.18)	0.98	(0.87 to 1.11)	0.98	(0.85 to 1.13)	0.99	(0.84 to 1.14)
>=4	1.11	(0.97 to 1.27)	1.19	(1.04 to 1.36)	1.10	(0.96 to 1.26)	1.11	(0.94 to 1.30)	1.07	(0.92 to 1.24)
Dementia (Yes vs No)	0.94	(0.82 to 1.07)	0.91	(0.80 to 1.05)	0.93	(0.82 to 1.07)	0.91	(0.78 to 1.06)	0.95	(0.81 to 1.09)
COPD (Yes vs No)	1.07	(0.97 to 1.19)	1.05	(0.95 to 1.17)	1.07	(0.97 to 1.18)	1.08	(0.96 to 1.20)	1.11	(0.98 to 1.25)
Contacts with community nurses (Ref= 0 to 3)										
4 to 12	1.06	(0.98 to 1.15)	1.05	(0.97 to 1.14)			1.07	(0.98 to 1.18)	1.03	(0.94 to 1.14)
>=13	0.88	(0.90 to 0.98)	0.85	(0.76 to 0.95)			0.88	(0.78 to 0.98)	0.84	(0.76 to 0.92)
Contacts with community palliative care teams (Ref= 0 to 3)										
4 to 8	0.95	(0.82 to 1.08)	0.91	(0.80 to 1.04)			0.94	(0.81 to 1.09)	0.90	(0.77 to 1.06)
>=9	0.85	(0.69 to 1.04)	0.79	(0.63 to 0.99)			0.92	(0.74 to 1.14)	0.89	(0.70 to 1.12)
Days in hospital in the last 90 days	1.02	(1.01 to 1.02)			1.02	(1.01 to 1.02)	1.01	(1.01 to 1.02)	1.02	(1.01 to 1.02)
Contacts with community nurses (Continuous)					1.00	(0.99 to 1.00)				
Contacts with community palliative care teams (Continuous)					0.99	(0.97 to 1.00)				
Ethnicity (Ref= white)										
Black									1.12	(0.97 to 1.31)
Asian									1.17	(1.06 to 1.30)
Mixed									1.14	(1.03 to 1.28)
Other									0.91	(0.76 to 1.09)

Table S3. Sensitivity analysis for one or more ED visits in the last 2 weeks of life

	Model 1		Model 2		Model 3		Model 5		Model 5	
	Original Model		Model 1 without days in hospital		Model 1 including contacts with PC practice as continu-ous		Model 1 for people with a record of cancer diagnosis and identification of pallia-tive care needs in the last 12 months of life		Model 1 with ethnicity	
	n=3441		n=3441		n=3441		n=2679		n-=2815	
	RR	95% CI	RR	95% CI	RR	95% CI	RR	95% CI	RR	95% CI
Age	0.99	(0.99 to 0.99)	0.99	(0.99 to 1.00)	0.99	(0.99 to 1.00)	0.99	(0.99 to 1.00)	0.99	(0.99 to 1.00)
Gender (Male vs female)	1.10	(0.98 to 1.23)	1.10	(0.98 to 1.23)	1.10	(0.98 to 1.23)	1.02	(0.89 to 1.17)	1.09	(0.96 to 1.24)
IMD quintile (Ref=1)										
2	1.01	(0.89 to 1.15)	1.01	(0.89 to 1.15)	1.00	(0.88 to 1.15)	1.03	(0.88 to 1.20)	1.03	(0.88 to 1.21)
3	0.93	(0.80 to 1.15)	0.93	(0.79 to 1.09)	0.93	(0.79 to 1.08)	0.91	(0.75 to 1.10)	0.94	(0.79 to 1.13)
4	1.00	(0.84 to 1.18)	0.99	(0.83 to 1.17)	1.00	(0.84 to 1.18)	0.99	(0.81 to 1.22)	0.97	(0.79 to 1.18)
5	0.99	(0.79 to 1.22)	0.97	(0.78 to 1.21)	0.98	(0.78 to 1.19)	1.03	(0.81 to 1.30)	0.93	(0.70 to 1.23)
Living in care home (Yes vs no)	0.70	(0.49 to 0.99)	0.69	(0.49 to 0.98)	0.70	(0.49 to 1.19)	0.71	(0.49 to 1.02)	0.76	(0.51 to 1.13)
Type of cancer (Ref=Bowel)										
Lung	1.01	(0.85 to 1.19)	1.00	(0.84 to 1.19)	1.01	(0.85 to 1.20)	1.03	(0.85 to 1.26)	0.99	(0.82 to 1.21)
Prostate	0.89	(0.72 to 1.10)	0.90	(0.73 to 1.11)	0.89	(0.72 to 1.09)	0.92	(0.71 to 1.19)	0.91	(0.71 to 1.16)
Breast	1.16	(0.94 to 1.42)	1.17	(0.95 to 1.43)	1.16	(0.94 to 1.42)	1.19	(0.92 to 1.52)	1.14	(0.90 to 1.44)
Pancreas	0.82	(0.63 to 1.06)	0.82	(0.63 to 1.06)	0.82	(0.63 to 1.06)	0.80	(0.60 to 1.07)	0.83	(0.63 to 1.11)
Haematological	0.93	(0.68 to 1.26)	0.97	(0.71 to 1.31)	0.94	(0.69 to 1.27)	0.84	(0.57 to 1.24)	0.90	(0.63 to 1.27)
Other	0.82	(0.70 to 0.96)	0.83	(0.71 to 0.96)	0.83	(0.71 to 0.96)	0.84	(0.69 to 1.01)	0.84	(0.70 to 1.00)
Number of QoF comorbidities (Ref=0)										
1	0.88	(0.75 to 1.04)	0.89	(0.75 to 1.05)	0.88	(0.74 to 1.03)	0.94	(0.78 to 1.13)	0.88	(0.73 to 1.06)
2	1.05	(0.89 to 1.25)	1.06	(0.89 to 1.26)	1.05	(0.89 to 1.25)	1.08	(0.89 to 1.31)	0.95	(0.78 to 1.16)
3	1.01	(0.84 to 1.21)	1.02	(0.85 to 1.23)	1.01	(0.84 to 1.22)	1.03	(0.83 to 1.28)	0.97	(0.79 to 1.20)
>=4	1.09	(0.89 to 1.32)	1.10	(0.90 to 1.34)	1.09	(0.89 to 1.32)	1.11	(0.88 to 1.39)	1.04	(0.83 to 1.30)
Chronic Heart Disease (yes vs no)	1.14	(0.99 to 1.31)	1.14	(1.00 to 1.31)	1.13	(0.99 to 1.30)	1.13	(0.97 to 1.31)	1.16	(1.00 to 1.34)
Contacts with the primary care practice (Ref= 0 to 3)										
4 to 10	1.10	(0.98 to 1.22)	1.10	(0.98 to 1.22)			1.13	(1.00 to 1.27)	1.17	(1.04 to 1.31)
>=11	1.27	(1.10 to 1.47)	1.27	(1.10 to 1.47)			1.35	(1.15 to 1.57)	1.35	(1.14 to 1.60)
Contacts with community nurses (Ref= 0 to 3)										
4 to 12	0.96	(0.85 to 1.08)	0.96	(0.85 to 1.08)			0.92	(0.80 to 1.06)	0.99	(0.87 to 1.14)
>=13	0.79	(0.68 to 0.92)	0.78	(0.67 to 0.91)			0.80	(0.68 to 0.93)	0.74	(0.62 to 0.87)
Contacts with community palliative care teams (Ref= 0 to 3)										
4 to 8	1.01	(0.85 to 1.21)	1.00	(0.84 to 1.20)			1.01	(0.81 to 1.26)	0.91	(0.72 to 1.15)
>=9	0.78	(0.56 to 1.08)	0.76	(0.55 to 1.06)			0.90	(0.66 to 1.24)	0.75	(0.49 to 1.12)
Days in hospital in the last 90 days	1.00	(1.00 to 1.01)			1.00	(1.00 to 1.00)	1.00	(1.00 to 1.01)	1.00	(1.00 to 1.01)
Contacts with primary care practice (continuous)					1.01	(1.01 to 1.01)				
Contacts with community nurses (Continuous)					0.99	(0.99 to 0.99)				
Contacts with community palliative care teams (Continuous)					0.99	(0.97 to 1.01)				
Ethnicity (Ref= white)										
Black									1.28	(1.05 to 1.55)

Asian					1.29 (1.11 to 1.50)
Mixed					0.99 (0.84 to 1.17)
Other					1.00 (0.76 to 1.29)

For peer review only

STROBE Statement—Checklist of items that should be included in reports of ***cohort studies***

	Item No	Recommendation	Page No
**Title and abstract**	1	(*a*) Indicate the study's design with a commonly used term in the title or the abstract	1
		(*b*) Provide in the abstract an informative and balanced summary of what was done and what was found	2
**Introduction**			
Background/rationale	2	Explain the scientific background and rationale for the investigation being reported	3
Objectives	3	State specific objectives, including any prespecified hypotheses	3
**Methods**			
Study design	4	Present key elements of study design early in the paper	4-5
Setting	5	Describe the setting, locations, and relevant dates, including periods of recruitment, exposure, follow-up, and data collection	4-5
Participants	6	(*a*) Give the eligibility criteria, and the sources and methods of selection of participants. Describe methods of follow-up	4-5
		(*b*) For matched studies, give matching criteria and number of exposed and unexposed	n/a
Variables	7	Clearly define all outcomes, exposures, predictors, potential confounders, and effect modifiers. Give diagnostic criteria, if applicable	5-6
Data sources/ measurement	8*	For each variable of interest, give sources of data and details of methods of assessment (measurement). Describe comparability of assessment methods if there is more than one group	5-6 and supplementary material
Bias	9	Describe any efforts to address potential sources of bias	4-6
Study size	10	Explain how the study size was arrived at	6 and supplementary material
Quantitative variables	11	Explain how quantitative variables were handled in the analyses. If applicable, describe which groupings were chosen and why	4-6
Statistical methods	12	(*a*) Describe all statistical methods, including those used to control for confounding	5-6
		(*b*) Describe any methods used to examine subgroups and interactions	5-6
		(*c*) Explain how missing data were addressed	5-6
		(*d*) If applicable, explain how loss to follow-up was addressed	n/a
		(*e*) Describe any sensitivity analyses	6
**Results**			

For peer review only

Participants	13*	(a) Report numbers of individuals at each stage of study—eg numbers potentially eligible, examined for eligibility, confirmed eligible, included in the study, completing follow-up, and analysed	6 and supplementary material
		(b) Give reasons for non-participation at each stage	6 and supplementary material
		(c) Consider use of a flow diagram	supplementary material
Descriptive data	14*	(a) Give characteristics of study participants (eg demographic, clinical, social) and information on exposures and potential confounders	6 and table 1
		(b) Indicate number of participants with missing data for each variable of interest	6 and table 1
		(c) Summarise follow-up time (eg, average and total amount)	4-6
Outcome data	15*	Report numbers of outcome events or summary measures over time	6 and table 1
Main results	16	(*a*) Give unadjusted estimates and, if applicable, confounder-adjusted estimates and their precision (eg, 95% confidence interval). Make clear which confounders were adjusted for and why they were included	8-9, table 2 and 3
		(*b*) Report category boundaries when continuous variables were categorized	8 and table 2 and 3
		(*c*) If relevant, consider translating estimates of relative risk into absolute risk for a meaningful time period	table 2 and 3
Other analyses	17	Report other analyses done—eg analyses of subgroups and interactions, and sensitivity analyses	10 and supplementary material
**Discussion**			
Key results	18	Summarise key results with reference to study objectives	12
Limitations	19	Discuss limitations of the study, taking into account sources of potential bias or imprecision. Discuss both direction and magnitude of any potential bias	13-14
Interpretation	20	Give a cautious overall interpretation of results considering objectives, limitations, multiplicity of analyses, results from similar studies, and other relevant evidence	12-13
Generalisability	21	Discuss the generalisability (external validity) of the study results	13
**Other information**			
Funding	22		18

*Give information separately for exposed and unexposed groups.

Note: An Explanation and Elaboration article discusses each checklist item and gives methodological background and published examples of transparent reporting. The STROBE checklist is best used in conjunction with this article (freely available on the Web sites of PLoS Medicine at http://www.plosmedicine.org/, Annals of Internal Medicine at http://www.annals.org/, and Epidemiology at http://www.epidem.com/). Information on the STROBE Initiative is available at http://www.strobe-statement.org.

For peer review only - http://bmjopen.bmj.com/site/about/guidelines.xhtml

BMJ Open

The association of primary and community care services with emergency visits and hospital admissions at the end of life in people with cancer: a retrospective cohort study.

Journal:	*BMJ Open*
Manuscript ID	bmjopen-2021-054281.R1
Article Type:	Original research
Date Submitted by the Author:	18-Dec-2021
Complete List of Authors:	Leniz, Javiera; King's College London, Cicely Saunders Institute of Palliative Care, Policy & Rehabilitation<br>Henson, Lesley; King's College London, Palliative Care, Policy & Rehabilitation<br>Potter, Jean; Hillingdon Hospitals NHS Foundation Trust, Department of Palliative Care<br>Gao, Wei; King's College London, Palliative Care, Policy & Rehabilitation<br>Newsom-Davis, Tom; Chelsea and Westminster Hospital NHS Foundation Trust, Oncology<br>Ul-Haq, Zia; Imperial College Health Partners, Discover-Now<br>Lucas, Amanda; Imperial College Health Partners, Discover-Now<br>Higginson, Irene; King's College London, Palliative Care and Policy<br>Sleeman, Katherine; King's College London, Dept of Palliative Care, Policy & Rehabilitation
**Primary Subject Heading**:	Palliative care
Secondary Subject Heading:	General practice / Family practice, Health services research, Palliative care, Public health
Keywords:	PRIMARY CARE, PALLIATIVE CARE, Quality in health care < HEALTH SERVICES ADMINISTRATION & MANAGEMENT, Epidemiology < ONCOLOGY

SCHOLARONE™
Manuscripts

I, the Submitting Author has the right to grant and does grant on behalf of all authors of the Work (as defined in the below author licence), an exclusive licence and/or a non-exclusive licence for contributions from authors who are: i) UK Crown employees; ii) where BMJ has agreed a CC-BY licence shall apply, and/or iii) in accordance with the terms applicable for US Federal Government officers or employees acting as part of their official duties; on a worldwide, perpetual, irrevocable, royalty-free basis to BMJ Publishing Group Ltd ("BMJ") its licensees and where the relevant Journal is co-owned by BMJ to the co-owners of the Journal, to publish the Work in this journal and any other BMJ products and to exploit all rights, as set out in our licence.

The Submitting Author accepts and understands that any supply made under these terms is made by BMJ to the Submitting Author unless you are acting as an employee on behalf of your employer or a postgraduate student of an affiliated institution which is paying any applicable article publishing charge ("APC") for Open Access articles. Where the Submitting Author wishes to make the Work available on an Open Access basis (and intends to pay the relevant APC), the terms of reuse of such Open Access shall be governed by a Creative Commons licence – details of these licences and which Creative Commons licence will apply to this Work are set out in our licence referred to above.

Other than as permitted in any relevant BMJ Author's Self Archiving Policies, I confirm this Work has not been accepted for publication elsewhere, is not being considered for publication elsewhere and does not duplicate material already published. I confirm all authors consent to publication of this Work and authorise the granting of this licence.

For peer review only

Title

The association of primary and community care services with emergency visits and hospital admissions at the end of life in people with cancer: a retrospective cohort study

Authors

1. **Javiera Leniz***
   King's College London, Cicely Saunders Institute of Palliative Care, Policy and Rehabilitation. Bessemer Road, London SE5 0PJ, United Kingdom. javiera.martelli@kcl.ac.uk
2. Lesley A Henson
   King's College London, Cicely Saunders Institute of Palliative Care, Policy and Rehabilitation.
3. Jean Potter
   Department of Palliative Care, The Furze, The Hillingdon Hospital NHS Foundation Trust
4. Wei Gao
   King's College London, Cicely Saunders Institute of Palliative Care, Policy and Rehabilitation.
5. Thomas Newsom-Davis
   Chelsea & Westminster Hospital NHS Foundation Trust
6. Zia Ul-Haq
   Discover-Now, Imperial College Health Partners
7. Amanda Lucas
   Discover-Now, Imperial College Health Partners
8. Irene J Higginson
   King's College London, Cicely Saunders Institute of Palliative Care, Policy and Rehabilitation.
9. Katherine E Sleeman
   King's College London, Cicely Saunders Institute of Palliative Care, Policy and Rehabilitation.

***Corresponding author**

Word count: 3,421

For peer review only

Abstract

Objective: to examine the association between primary and community care use and measures of acute hospital use in people with cancer at the end of life.

Design: retrospective cohort study.

Setting: we used Discover, a linked administrative and clinical dataset from general practices, community and hospital records in North West London (UK).

Participants: people registered in general practices, with a diagnosis of cancer who died between 2016-2019.

Primary and secondary outcome measures: >=3 hospital admissions during the last 90 days, >=1 admissions in the last 30 days and >=1ED visit in the last 2 weeks of life.

Results: of 3581 people, 490 (13.7%) had >=3 admissions in last 90 days, 1640 (45.8%) had >=1 admission in the last 30 days, 1042 (28.6%) had >=1 ED visits in the last 2 weeks; 1069 (29.9%) had more than one of these indicators. Contacts with community nurses in the last three months (>=13 vs <4) was associated with fewer admissions in the last 30 days (RR 0.88, 95% CI 0.79-0.97) and ED visits in the last 2 weeks of life (RR 0.79, 95% CI 0.68-0.92). Contacts with GPs in the last three months (>=11 vs <4) was associated with higher risk of >=3 admissions in the last 90 days (RR 1.63, 95% CI 1.33-1.99) and ED visits in the last 2 weeks of life (RR 1.27, 95% CI 1.10-1.47).

Conclusions: expanding community nursing could reduce acute hospital use at the end of life and improve quality of care.

Keywords

Primary health care, end-of-life care, inappropriate acute care, hospital admissions, community care, cancer.

Strengths and limitations of this study

- Population-based cohort study using a large and comprehensive dataset that holds information on healthcare services use from eight different boroughs in London and over 2 million people.
- Our study examined data from local authorities and general practice records, which provides a unique opportunity to describe community and primary care service utilization.
- People in the cohort might have not died from cancer but from other conditions, as information on cause of death was not available.
- The overall use of the primary care practice and palliative care community teams are likely to be underestimated in this study due to the methods used to estimate contacts.

For peer review only

- Information on the quality of care or the appropriateness of hospital admissions and ED visits was not available and are likely to be confounders.

Background

While mortality rates for most types of cancer have decreased, globally deaths from cancer increased by 25.4% between 2007 and 2017 due to population ageing and growth.[1] A similar pattern is observed in the United Kingdom, and more than 95,000 deaths due to cancer are projected for 2035, 24.5% more than in 2014.[2] It is therefore critical to understand how to provide high-quality end-of-life care for people with cancer.

Excessive use of hospital care in the last months of life has been proposed as an indicator of the quality of end-of-life cancer care.[3] This is because emergency hospital care is associated with reduced quality of life and care satisfaction in cancer patients and their families,[4-6] without contributing to an improvement in survival.[7, 8] Despite these negative outcomes, hospital admissions and emergency department (ED) visits at the end of life have increased over time in cancer patients nearing the end of their life.[9]

Sociodemographic and illness-related factors have been found to be associated with higher hospital admissions and ED attendances in the last months of life, for example being male, black, having lung cancer and low socioeconomic status.[10-12] On the other hand, access to palliative care services have been associated with lower acute end-of-life care, suggesting these services could help prevent unnecessary admissions to hospital.[10, 13] However, little is known about how contacts with general practitioners in primary care practices and other community services such as community nurses, community palliative care or rehabilitation teams influence acute care use near the end of life among people with cancer. The aim of this study was to describe the association between primary and community care services use with three measures of acute hospital use for people with cancer at the end of life.

Methods

Design and Data sources

This is a retrospective cohort study using the Discover dataset, one of Europe's largest linked longitudinal de-identified datasets that includes 95% of all patients registered with a general practitioner in North West London.[14] The Discover dataset is a platform that enables researcher access to pseudonymised patient-level data drawn from the Whole Systems Integrated Care (WSIC) local data warehouse for research purposes. Discover dataset is maintained and interrogated on a secure server and extracts of

For peer review only - http://bmjopen.bmj.com/site/about/guidelines.xhtml

data are then aggregated in compliance with the Information Governance suppression rule where numbers below 5 are annotated as <5. In this process, the de-identified data is rendered anonymised by stripping out any information that would allow re-identification of an individual's identity. Discover dataset is accessible via Discover-NOW Health Data Research Hub for Real World Evidence through their data scientist specialists and IG committee-approved analysts, hosted by Imperial College Health Partners.

In June 2019, the database held records for a total of 2.37 million patients spread across eight Clinical Commissioning Groups (CCGs). The estimated total population for the eight boroughs contributing data to Discover was 2.1M in mid-2019. Differences in the population estimated and the number of patients in the dataset could be explained by people being enrolled in a GP practice contributing to the dataset but whose usual place of residence is in another area. Of 370 health and social care provider organisations from the National Health Service (NHS) in North West London, 359 (97%) have a data sharing agreement and submit their records to the dataset. Organisations feeding records to the dataset include primary care practices, mental health, community trusts and hospital care attended by North West London patients, and exclude private and third sector providers, such as hospices services.[14] We chose the Discover dataset as it is a comprehensive population-based dataset and provides access to community care records in addition to primary and hospital care records, which are not generally available for other primary care datasets in the UK. The age and gender distribution and prevalence of long-term conditions of the Discover population are similar to the rest of London and the UK.[14]

Population

Adults (aged 18 or over) included in the Discover dataset with at least one record of a cancer diagnosis recorded at any point from 1st January 2015 onwards in primary care practice or hospital in-patient records using Read Codes and International Statistical Classification of Diseases and Related Health Problems (ICD) 10 codes respectively (Codes available in Supplemental Box S1). We included in the cohort people who died between 2016 and 2019 based on the date of death recorded in primary care or hospital records. As we did not have information on the cause of death or cancer severity, we restricted our sample to people who had been identified as having palliative care needs in primary care records at any time based on the Quality of Outcomes Framework (QoF) Read Codes for the Palliative Care register,[15] to include people whose death could be considered expected rather than sudden (Codes available in Supplemental Box S1).

For peer review only

Outcomes

We evaluated three measures of acute hospital use towards the end of life. We chose these three outcome measures as their prevalence at a population-level can be considered an indicator of end of life care quality according to Henson et al systematic review,[3] their focus on acute care use at the end of life, and the feasibility to be measured in the data. The three measures were:

1. Three or more emergency hospital admissions in the last 90 days of life.[16]

2. One or more emergency hospital admissions in the last 30 days of life. [3]

3. One or more emergency department (ED) visits in the last two weeks of life.[3]

Explanatory variables

-Primary care practice contacts: we identified contacts with the primary care practice in the last 90 days of life using a similar approach reported by Kontopantelis et al (2015).[17] We considered only direct consultations such as telephone, face-to-face or home visits, and excluded administrative consultations or non-attended appointments. It was not possible to identify whether the contact in the practice was with a doctor or another healthcare professional. Only one consultation in the same day was used to reduce the likelihood of including duplicate records, as it was not possible to determine whether records from the same day correspond to more than one contact or not. This approach has been widely used in research using primary care records in the UK.[17, 18] (Supplemental Box S2)

-Contacts with other community services: we identified contacts with community nurses, community palliative care teams and rehabilitation teams in the last 90 days of life based on the date of the contact and the description of the service. Contacts with rehabilitation teams included physiotherapy, speech and language and occupational therapy services. We removed non-attendant contacts and duplicates based on the date. We identified individuals who were defined by Discover primary care dataset as living in a care home based on the latest patient record (Supplemental Box S3).

Co-variables

-Sociodemographic: Age at death, gender, ethnicity and Index of Multiple Deprivation (IMD) were extracted from Discover dataset records for each individual. The 2015 IMD was derived at Lower Super Output Areas (LSOAs) from the patients' last address registered in the system and reported according to The English Indices of Deprivation 2015 guidance.[19]

-Illness-related: the number of comorbidities was calculated using the count of 15 QoF chronic diseases (excluding cancer) identified from Read codes in the primary care practice records.[15] The type of cancer

For peer review only

was identified from the primary care practice and hospital in-patient records using Read Codes and ICD-10 codes respectively (Supplemental Box S1). Only 6% of the cohort had more than one cancer recorded and were included in the 'Other' category.

- Number of days in hospital: we calculated the number of days patients spent in hospital in the last 90 days of life using in-patient hospital codes for spells' start dates and discharge dates.

Analysis

Data were described using count and percentage for categorical variables and mean and standard deviation for continuous variables. A Pearson' s chi2 test for the trend for categorical variables and t-test and Wilcoxon rank-sum test for age and days in hospital respectively was used to evaluate the association between each variable and the outcomes.

We used generalized estimating equations (GEE) to estimate the unadjusted and multivariate association between sociodemographic, illness-related factors and contacts with primary and community care services in the last 90 days of life and each of the three indicators separately. We used Poisson family with log link function, exchangeable correlation structure and robust error variance with data clustered in primary care practices where patients were registered. For the multivariate model, we adjusted by age, gender, IMD quintile, care home residence, type of cancer and number of QoF comorbidities. We selected specific comorbidities, and primary and community care services use according to significance in unadjusted analysis ($p<=0.05$). We excluded ethnicity from the final model to avoid biasing the sample as the variable has a large proportion of missing data. To facilitate interpretation, we categorized primary and community care service contacts based on clinical judgement. Categories were approximately one or fewer contacts per month, more than one contact per month but less than one contact per week, and more than one contact per week, depending on the distribution. Because number of contacts with palliative care and rehabilitation teams were small, we adapted these categories. We included the number of days each person spent in hospital in the last 90 days of life as a continuous variable in the models to account for the fact that if someone is in hospital, they cannot receive care in the community.

We performed four sensitivity analysis: (1) to explore the influence of days in hospital by removing the variable from the model, (2) to understand the impact of categorization of primary and community care services in the model, we used the same model but with the corresponding primary and community care service use variables as continuous, (3) to explore the impact of restricting the sample to people with a record of cancer diagnosis and identification of palliative care needs in the last 12 months of life (instead of at any time), (4) to understand the influence of ethnicity in the model.

For peer review only

Patient and public involvement

The protocol was presented and discussed with patients and public representatives at the beginning of the study. A member of the public with experience caring for a relative who died with cancer joined the Project Advisory Group of the project, reviewing a lay version of the protocol and participated in the interpretation of results.

Results

Characteristics of the cohort

We identified 4933 people with a diagnosis of cancer and who died between 2016 and 2019. 3848 (78.0%) of them had a palliative care QoF record in primary care records. After removing 267 people with invalid dates of death and hospital admissions, 3581 people were included in the analysis (Supplemental Figure S1). The mean age was 76.6 (SD 13.3), 55.4% were male and 21.3% had four or more comorbidities. The most frequent cancer diagnosis was lung cancer (21.5%) followed by bowel (11.6%) and prostate cancer (8.6%) (Table 1).

Of the 3581 people in the sample, 490 (13.7%) had three or more emergency admissions in last 90 days, 1640 (45.8%) had one or more emergency admissions in the last 30 days, and 1042 (28.6%) had one or more ED visits in the last two weeks of life (Table 1). There was overlap between the three indicators with 1069 (29.9%) of the sample having more than one of the indicators and 269 (7.5%) of the cohort having all three. Older age, white ethnicity and living in a care home were associated with lower chances of all three outcomes (Table 1).

For peer review only

Table 1. Sample characteristics by outcome measure.

		All sample		3 or more EHA last 3 months					1 or more EHA last month					1 or more ED visit in the last 2 weeks of life				
				No		Yes			No		Yes			No		Yes		
		n=3,581		n=3091		n=490			n=1941		n=1640			n=2539		n=1042		
		n	%	n	%	n	%	p-value	n	%	n	%	p-value	n	%	n	%	p-value
Age	(Mean, sd)	76.61	13.32	77.32	13.06	72.18	14.08	<0.001	77.63	12.92	75.41	13.69	<0.001	77.21	13.31	75.16	13.26	<0.001
Gender	Female	1,596	44.6	1394	45.1	202	41.2	0.109	905	46.6	691	42.1	0.007	1155	45.5	441	42.3	0.083
	Male	1,985	55.4	1697	54.9	288	58.8		1036	53.4	949	57.9		1384	54.5	601	57.7	
Ethnicity	White	1,511	42.2	1330	43.0	181	36.9	0.001	866	44.6	645	39.3	0.006	1114	43.9	397	38.1	<0.001
	Black	232	6.5	195	6.3	37	7.6		115	5.9	117	7.1		147	5.8	85	8.2	
	Asian	490	13.7	396	12.8	94	19.2		240	12.4	250	15.2		313	12.3	177	17.0	
	Mixed	523	14.6	447	14.5	76	15.5		270	13.9	253	15.4		380	15.0	143	13.7	
	Other	177	4.9	149	4.8	28	5.7		104	5.4	73	4.5		127	5.0	50	4.8	
	Missing	648	18.1	574	18.6	74	15.1		346	17.8	302	18.4		458	18.0	190	18.2	
IMD quintile	1 (Most deprived)	604	16.9	499	16.1	105	21.4	0.047	319	16.4	285	17.4	0.613	414	16.3	190	18.2	0.536
	2	1,135	31.7	983	31.8	152	31.0		612	31.5	523	31.9		804	31.7	331	31.8	
	3	894	25.0	776	25.1	118	24.1		474	24.4	420	25.6		642	25.3	252	24.2	
	4	540	15.1	467	15.1	73	14.9		310	16.0	230	14.0		383	15.1	157	15.1	
	5 (Least deprived)	299	8.3	268	8.7	31	6.3		167	8.6	132	8.0		222	8.7	77	7.4	
	Missing	109	3.0	98	3.2	11	2.2		59	3.0	50	3.0		74	2.9	35	3.4	
Lived in care home	No	3,389	94.6	2910	94.1	479	97.8	0.001	1797	92.6	1592	97.1	<0.001	2387	94.0	1002	96.2	0.010
	Yes	192	5.4	181	5.9	11	2.2		144	7.4	48	2.9		152	6.0	40	3.8	
Number QoF comorbidities	0	579	16.2	489	15.8	90	18.4	0.184	304	15.7	275	16.8	0.041	398	15.7	181	17.4	0.036
	1	856	23.9	750	24.3	106	21.6		495	25.5	361	22.0		641	25.2	215	20.6	
	2	756	21.1	657	21.3	99	20.2		401	20.7	355	21.6		534	21.0	222	21.3	
	3	628	17.5	529	17.1	99	20.2		353	18.2	275	16.8		445	17.5	183	17.6	
	>=4	762	21.3	666	21.5	96	19.6		388	20.0	374	22.8		521	20.5	241	23.1	
QoF Comorbidities	COPD (yes)	535	14.9	456	14.8	79	16.1	0.429	266	13.7	269	16.4	0.024	371	14.6	164	15.7	0.390
	Depression (yes)	625	17.5	539	17.4	86	17.6	0.951	330	17.0	295	18.0	0.439	446	17.6	179	17.2	0.781
	Diabetes (yes)	996	27.8	855	27.7	141	28.8	0.609	522	26.9	474	28.9	0.181	682	26.9	314	30.1	0.047
	Hypertension (yes)	2022	56.5	1753	56.7	269	54.9	0.452	1101	56.7	921	56.2	0.734	1441	56.8	581	55.8	0.585
	Dementia (yes)	328	9.2	300	9.7	28	5.7	0.004	202	10.4	126	7.7	0.005	246	9.7	82	7.9	0.086
	CHD (yes)	689	19.2	596	19.3	93	19.0	0.875	354	18.2	335	20.4	0.098	463	18.2	226	21.7	0.017
Type of cancer	Bowel	416	11.6	374	12.1	42	8.6	0.075	234	12.1	182	11.1	0.215	288	11.3	128	12.3	0.032
	Lung	769	21.5	646	20.9	123	25.1		392	20.2	377	23.0		520	20.5	249	23.9	
	Prostate	309	8.6	260	8.4	49	10.0		169	8.7	140	8.5		223	8.8	86	8.3	
	Breast	237	6.6	205	6.6	32	6.5		117	6.0	120	7.3		155	6.1	82	7.9	
	Pancreas	194	5.4	167	5.4	27	5.5		103	5.3	91	5.5		142	5.6	52	5.0	
	Haematological	137	3.8	114	3.7	23	4.7		75	3.9	62	3.8		95	3.7	42	4.0	
	Other	1519	42.4	1325	42.9	194	39.6		851	43.8	668	40.7		1116	44.0	403	38.7	

QoF: Quality of Outcomes Framework, IMD: Index of Multiple Deprivation, EHA: hospital admissions; ED: Emergency department visits; CHD: Chronic heart disease.

Primary care and other community services

On average, people in the cohort had 2.3 (SD 3.3) telephone and 2.4 (SD 3.3) face-to-face consultations with the primary care practice, 8.6 (SD 13.7) contacts with community nurses, 1.2 (SD 3.0) contacts with community palliative care teams and 0.3 (SD 1.2) contacts with rehabilitation services in the last 90 days of life.

People with three or more hospital admissions in the last 90 days and ED visits in the last two weeks of life also had more contacts in the primary care practice. Conversely, people with hospital admissions in the last 30 days and ED visits in the last two weeks of life had fewer contacts with community nurses and palliative care teams (Table 2).

For peer review only

Table 2. Health care services utilization in the last three months of life by outcome measure.

	3 or more EHA last 3 months					1 or more EHA last month					1 or more ED visits in the last 2 weeks				
	No		Yes			No		Yes			No		Yes		
	No.	%	No.	%	p-value(1)	No.	%	No.	%	p-value(1)	No.	%	No.	%	p-value(1)
**Contacts with GP practice**															
0 to 3	1812	58.6	243	49.6		1144	58.9	911	55.5		1499	59.0	556	53.4	
4 to 10	873	28.2	149	30.4		539	27.8	483	29.5		705	27.8	317	30.4	
>=11	406	13.1	98	20.0	<0.001	258	13.3	246	15.0	0.105	355	14.0	169	16.2	0.014
**Contacts with community nurses**															
0 to 3	1629	52.7	244	49.8		995	51.3	878	53.5		1300	51.2	573	55.0	
4 to 12	712	23.0	123	25.1		420	21.6	415	25.3		582	22.9	253	24.3	
>=13	725	23.5	117	23.9	0.475	504	26.0	338	20.6	<0.001	632	24.9	210	20.2	0.008
**Contacts with community palliative care teams**															
0 to 3	2,652	85.8	432	88.2		1644	84.7	1440	87.8		2170	85.5	914	87.7	
4 to 8	313	10.1	40	8.2		206	10.6	147	9.0		254	10.0	99	9.5	
>=9	126	4.1	18	3.7	0.350	91	4.7	53	3.2	0.017	115	4.5	29	2.8	0.044
**Contacts with rehabilitation teams**															
0	2830	91.6	450	91.8		1760	90.7	1520	92.7		2321	91.4	959	92.0	
1 to 3	186	6.0	29	5.9		127	6.5	88	5.4		153	6.0	62	6.0	
>=4	75	2.4	11	2.2	0.966	54	2.8	32	2.0	0.082	65	2.6	21	2.0	0.622
**Days in hospital**					p-value(2)					p-value(2)					p-value(2)
Mean (SD)	11.85	(14.15)	24.98	(13.32)		10.21	(14.9)	17.71	(13.48)		13.1	(15.21)	14.98	(13.46)	
Median (IQR)	7	(0.0-19.0)	23	(15.0-32.0)	<0.001	2	(0.0-16.0)	15	(8.0-25.0)	<0.001	8	(0.0-20.0)	11	(5.0-21.0)	<0.001

EHA: hospital admissions; ED: Emergency department visits, GP: General practitioner. (1) Chi-2 for trend p-value, (2) Wilcoxon rank-sum test p-value.

Multivariate analysis

In the multivariate analysis, people with three or more hospital admissions in the last 90 days were more likely to be younger, have lung or prostate cancer, have more than 11 contacts with the primary care practice in the last 90 days and were less likely to live in a care home. People with one or more admissions in the last 30 days, were more likely to be younger, male, have breast cancer, they were less likely to live in a care home, and less likely to have more than 13 contacts with community nurses in the last 90 days. Having one or more ED visits in the last two weeks of life was associated with being younger, having fewer contacts with community nurses, more contacts with the primary care practice and lower chances of living in a care home (Table 3).

The sensitivity analyses for the outcomes three or more hospital admissions in the last 90 days and ED visits demonstrated similar results (Supplemental tables S1 and S3). In the sensitivity analyses for one or more admissions in the last 30 days, the number of contacts with community nurses analysed as a continuous variable was no longer associated with the outcome measure, suggesting a threshold response (Supplemental table S2).

For peer review only - http://bmjopen.bmj.com/site/about/guidelines.xhtml

Table 3. Association between sociodemographic, illness-related and service-related factors with three outcome measures for acute end-of-life care in the last three months of life.

	3 or more EHA last 3 months n=3,472				1 or more EHA last month n=3,441				1 or more ED last 2 weeks n=3,441			
	Univariate		Multivariate		Univariate		Multivariate		Univariate		Multivariate	
	RR	95% CI	RR	95% CI	RR	95% CI	RR	95% CI	RR	95% CI	RR	95% CI
Age	0.98	(0.97 to 0.99)	0.98	(0.97 to 0.99)	0.99	(0.99 to 0.10)	0.99	(0.99 to 1.00)	0.99	(0.99 to 1.00)	0.99	(0.99 to 1.00)
Gender (Ref Female)												
Male	1.15	(0.97 to 1.36)	1.10	(0.92 to 1.31)	1.10	(1.02 to 1.20)	1.11	(1.02 to 1.20)	1.09	(0.98 to 1.21)	1.10	(0.98 to 1.23)
IMD quintile (Ref 1, most deprived)												
2	0.79	(0.63 to 0.99)	0.82	(0.65 to 1.03)	1.00	(0.90 to 1.12)	1.01	(0.91 to 1.12)	0.97	(0.84 to 1.11)	1.01	(0.89 to 1.15)
3	0.80	(0.62 to 1.02)	0.88	(0.70 to 1.12)	1.01	(0.90 to 1.13)	1.03	(0.92 to 1.15)	0.90	(0.77 to 1.06)	0.93	(0.80 to 1.15)
4	0.82	(0.62 to 1.08)	0.96	(0.75 to 1.24)	0.92	(0.81 to 1.05)	0.96	(0.85 to 1.09)	0.95	(0.80 to 1.13)	1.00	(0.84 to 1.18)
5	0.68	(0.49 to 0.93)	0.95	(0.68 to 1.31)	0.97	(0.81 to 1.16)	1.06	(0.90 to 1.25)	0.88	(0.71 to 1.10)	0.98	(0.79 to 1.22)
Lived in care home (Ref no)	0.43	(0.24 to 0.78)	0.53	(0.28 to 0.98)	0.54	(0.41 to 0.72)	0.54	(0.41 to 0.72)	0.71	(0.51 to 0.99)	0.70	(0.49 to 0.99)
Type of cancer (Ref Bowel)												
Lung	1.53	(1.13 to 2.06)	1.60	(1.16 to 2.20)	1.11	(0.98 to 1.25)	1.08	(0.96 to 2.23)	1.04	(0.88 to 1.22)	1.01	(0.85 to 1.19)
Prostate	1.57	(1.09 to 2.28)	1.57	(1.07 to 2.30)	1.03	(0.89 to 1.21)	0.98	(0.84 to 2.15)	0.91	(0.74 to 1.11)	0.89	(0.72 to 1.10)
Breast	1.32	(0.88 to 1.97)	1.24	(0.82 to 1.87)	1.15	(0.98 to 1.34)	1.19	(1.02 to 1.39)	1.11	(0.93 to 1.34)	1.16	(0.94 to 1.42)
Pancreas	1.34	(0.88 to 2.04)	1.23	(0.93 to 2.08)	1.05	(0.89 to 1.25)	1.02	(0.87 to 2.20)	0.85	(0.67 to 1.09)	0.82	(0.63 to 1.06)
Haematological	1.63	(1.01 to 2.61)	1.23	(0.73 to 2.07)	1.03	(0.83 to 1.29)	0.91	(0.72 to 2.15)	0.98	(0.74 to 1.31)	0.93	(0.68 to 1.26)
Other	1.24	(0.91 to 1.69)	1.15	(0.83 to 1.58)	1.00	(0.88 to 1.12)	0.96	(0.86 to 1.08)	0.85	(0.73 to 0.99)	0.82	(0.70 to 0.96)
Number QoF comorbidities (Ref 0)												
1	0.81	(0.64 to 1.03)	0.88	(0.69 to 1.11)	0.90	(0.81 to 1.00)	0.92	(0.83 to 1.02)	0.82	(0.70 to 0.96)	0.88	(0.75 to 1.04)
2	0.83	(0.65 to 1.07)	0.99	(0.76 to 1.30)	0.99	(0.89 to 1.10)	1.06	(0.95 to 1.18)	0.95	(0.81 to 1.11)	1.05	(0.89 to 1.25)
3	1.01	(0.79 to 1.29)	1.23	(0.94 to 1.62)	0.92	(0.82 to 1.04)	0.99	(0.87 to 1.12)	0.93	(0.79 to 1.10)	1.01	(0.84 to 1.21)
>=4	0.80	(0.62 to 1.04)	0.98	(0.74 to 1.30)	1.04	(0.92 to 1.17)	1.11	(0.97 to 1.27)	1.01	(0.86 to 1.18)	1.09	(0.89 to 1.32)
QoF Comorbidities (Ref no)												
COPD	1.09	(0.88 to 1.34)	-	-	1.11	(1.01 to 1.23)	1.07	(0.97 to 1.19)	1.05	(0.91 to 1.21)	-	-
Depression	1.01	(0.84 to 1.22)	-	-	1.04	(0.94 to 1.14)	-	-	0.98	(0.86 to 1.11)	-	-
Diabetes	1.02	(0.86 to 1.22)	-	-	1.04	(0.97 to 1.12)	-	-	1.09	(0.97 to 1.22)	-	-
Hypertension	0.92	(0.79 to 1.07)	-	-	0.98	(0.92 to 1.05)	-	-	0.96	(0.87 to 1.07)	-	-
Dementia	0.62	(0.44 to 0.88)	0.78	(0.54 to 1.14)	0.84	(0.73 to 0.97)	0.94	(0.82 to 1.07)	0.86	(0.70 to 1.06)	-	-
CHD	0.99	(0.82 to 1.20)	-	-	1.08	(0.99 to 1.18)	-	-	1.16	(1.03 to 1.30)	1.14	(0.99 to 1.31)
Contacts with GP practice (Ref 0-3)												
4 to 10	1.21	(1.01 to 1.46)	1.18	(0.98 to 1.41)	1.05	(0.97 to 1.13)	-	-	1.12	(1.00 to 1.24)	1.10	(0.98 to 1.22)
>=11	1.59	(1.29 to 1.95)	1.63	(1.33 to 1.99)	1.08	(0.98 to 1.19)	-	-	1.19	(1.03 to 1.38)	1.27	(1.10 to 1.47)
Contacts with community nurses (Ref 0-3)												
4 to 12	1.08	(0.90 to 1.29)	-	-	1.04	(0.96 to 1.13)	1.06	(0.98 to 1.15)	0.97	(0.86 to 1.10)	0.96	(0.85 to 1.08)
>=13	1.01	(0.83 to 1.24)	-	-	0.84	(0.76 to 0.93)	0.88	(0.90 to 0.98)	0.80	(0.69 to 0.93)	0.79	(0.68 to 0.92)

For peer review only - http://bmjopen.bmj.com/site/about/guidelines.xhtml

Contacts with community palliative care teams (Ref 0-3)																
4 to 8	0.88	(0.68 to 1.15)	-	-	0.91	(0.80 to 1.04)	0.95	(0.82 to 1.08)	0.97	(0.82 to 1.14)	1.01	(0.85 to 1.21)				
>=9	0.93	(0.62 to 1.37)	-	-	0.78	(0.63 to 0.96)	0.85	(0.69 to 1.04)	0.70	(0.50 to 0.97)	0.78	(0.56 to 1.08)				
Contacts with rehabilitation teams (Ref 0)																
1to3	1.01	(0.73 to 1.38)	-	-	0.89	(0.76 to 1.05)	-	-	1.01	(0.82 to 1.25)	-	-				
>=4	0.93	(0.55 to 1.58)	-	-	0.80	(0.61 to 1.04)	-	-	0.81	(0.57 to 1.15)	-	-				
Days in hospital	0.94	(0.94 to 0.95)	1.04	(1.03 to 1.04)	1.02	(1.01 to 1.02)	1.02	(1.01 to 1.02)	1.00	(0.99 to 1.00)	1.00	(1.00 to 1.01)				

QoF: Quality of Outcomes Framework, IMD: Index of Multiple Deprivation, EHA: hospital admissions; ED: Emergency department visits; CHD: Chronic heart disease, COPD: Chronic Obstructive Pulmonary Disease; RR: risk ratio. – variable not included in the model.

For peer review only

Discussion

The three outcome measures for end-of-life cancer care evaluated (three or more admissions to hospital in the last 90 days, one or more hospital admissions in the last 30 days and one or more ED visits in the last two weeks of life) were frequent (13.7%, 45.8% and 28.6% respectively). We found that contacts with community nurses were associated with fewer ED visits in the last two weeks of life, and contacts with the primary care practice were associated with higher risk of multiple admissions to hospital in the last 90 days and ED visits in the last two weeks of life.

We found contacts with community nurses were associated with a lower risk of hospital admissions and ED visits at the end of life. These findings are consistent with previous studies where more community nursing hours per week were associated with lower odds of hospital admissions and ED visits at the end of life among patients with cancer in Canada.[20, 21] Community nurses have an important role providing physical care, managing symptoms and medications, educating and giving information to patients and families, and coordinating care,[22-25] and therefore they could play an important role in avoiding unnecessary hospital admissions at the end of life in people with cancer.

People with cancer that were living in care homes before death had a lower risk of hospital admissions and ED visits at the end of life in this cohort. The number of people living in care homes in our sample was small, and we did not have information on the level of health care needs of this group of people. Nevertheless, these findings are consistent with other international studies showing that people living in long-term facilities are less likely to have transitions to hospital regardless of the cause of death.[26, 27] Long-term facilities are one of the very few care settings in the community providing continuous care including out-of-hours.[28, 29] More research is needed to understand the mechanisms that explain this association, as well as to explore differences in health care provision and health care needs between people living in care homes and in the community.

In contrast with previous studies,[20, 30] we found that contacts with the primary care practice were associated with higher risk of multiple admissions to hospital in the last 90 days and ED visits in the last two weeks of life. This is likely to be explained by complexity of health care needs: more severe and complex patients are likely to have a higher use of healthcare services.[31] A rise in the number of contacts with the practice could be an opportunity to identify patients who are deteriorating or whose health care needs are increasing. High healthcare use can also be an indicator of unmet needs, ineffective and uncoordinated care and lead to poor patient satisfaction.[32-34] Having many different healthcare professionals could cause confusion among patients and their caregivers and lead to more consultations. It is possible that more

For peer review only - http://bmjopen.bmj.com/site/about/guidelines.xhtml

contacts with the primary care practice in this sample reflects poor coordination or a lack of continuity of care, leading to more admissions to hospital.

Implications for research and/or practice

Primary care physicians play a key role in providing care for people approaching the end of life. Their involvement is valued by patients and families,[35] and has shown to improve end-of-life care outcomes.[36, 37] However, several barriers to palliative care in general practice have been identified, such as the increasing workload and time, lack of funding, poor communication with specialists and lack of experience and training.[38, 39] More research is needed to explore effective models of end-of-life care in primary care and palliative care integration in order to address the increasing demand for care and complexity of health care needs that patients experience when approaching the end of life.

The three measures used in this study have been proposed as quality indicators for cancer end-of-life care.[3, 40] Measuring the quality of care provided by health care services is key to monitor and promote the delivery of high-quality cancer care. We found an overlap between these indicators, with 29.9% patients having more than one and different predictors associated with each of them, which suggests a balanced combination of quality indicators might be needed to measure the quality of care provided by health care services. Although the measures chosen are recognised quality indicators and important when evaluating quality, they only represent one component of quality at a population level and should be considered alongside other measures of quality such as patient experience and patient reported outcome measures (PROMs).

Strengths and limitations

The Discover dataset holds comprehensive information on healthcare services use from eight different boroughs in London and over 2 million people, including information on primary, community and hospital care. However, our cohort is limited to a London population, which could limit the generalizability of the results.

A limitation of this study is the lack of information on cause of death, time for diagnosis and stage of cancer. Some of the people included might have not died from cancer but from other conditions, and this could vary between different cancer groups. We tried to address this limitation by restricting the sample to people who had been identified as having palliative care needs. However, that approach could have biased the sample toward people with higher or more complex health care needs. We derived the date of death from primary care and hospital records, therefore some level of inaccuracy might be expected.[41] Primary care practice contacts were derived from Read codes and therefore it is possible the number of consultations with the practice was underestimated.[17, 18] We excluded administrative contacts and same

For peer review only - http://bmjopen.bmj.com/site/about/guidelines.xhtml

day records with the primary care practice, as it has been done in other studies, for technical reasons. This approach might underestimate the overall contribution of primary care practices in this study.

We did not have information on the quality of care, continuity, coordination of care or the appropriateness of hospital admissions. Likewise, it was not possible to determine the length of stay or how close to death a person was admitted to the care home facility. These factors could also have an impact on the outcomes of this study. It is likely that some palliative care services were not fully identified, as community palliative care is often provided by the third sector in England, and therefore not consistently included in administrative records.

Conclusions

In this population-based cohort study of people with cancer, multiple emergency admissions to hospital in the last 90 days, admissions in the last 30 days and ED visits in the last two weeks of life were frequent. Contacts with community nurses were associated with fewer hospital admissions in the last 30 days of life and fewer ED visits in the last two weeks of life. More research is needed to explore effective models of end-of-life care in primary care and palliative care integration to address the complexity of the patient population with cancer cared for in primary and community care.

For peer review only

Additional information

Funding statement

J.L is funded by a Royal Marsden Partners Pan London Research Fellowship Award and the Programa Formacion de Capital Humano Avanzado, Doctorado Becas Chile, 2018 (folio 72190265). KES is funded by a National Institute of Health Research (NIHR) Clinician Scientist Fellowship (CS-2015-15-005) and is the Laing Galazka Chair in Palliative Care at King's College London, funded by an endowment from Cicely Saunders International and the Kirby Laing Foundation. IJH is an NIHR Senior Investigator Emeritus. IJH is supported by the NIHR Applied Research Collaboration South London (NIHR ARC South London) at King's College Hospital NHS Foundation Trust. IJH leads the Palliative and End of Life Care theme of the NIHR ARC South London, and co-leads the national theme in this. The views expressed are those of the authors and not necessarily those of the NHS, the NIHR, the Department of Health and Social Care or the funding charities.

Ethical approval

The source database is approved for secondary analysis by the West Midland-Solihull Research Ethic Committee (reference 18/WM/0323). All data was anonymised and therefore, no patient consent was required.

Competing interests

The Authors declare that there is no conflict of interest.

Authors' contribution

JL and KES had the idea for the study. JL designed the study with input from KES, ZU, AL and IJH. Data analysis was carried out by JL with input from KES, GW, LH, ZU, TN, JP and IJH. All authors helped interpret the data. JL wrote the first draft of the paper. All authors contributed to subsequent drafts and approved the final paper.

Data sharing statement

The data that support the findings of this study are available from the Discover dataset but restrictions apply to the availability of these data, which were used under license for the current study, and so are not publicly available.

For peer review only

References

1. Global, regional, and national age-sex-specific mortality for 282 causes of death in 195 countries and territories, 1980-2017: a systematic analysis for the Global Burden of Disease Study 2017. *Lancet* 2018; 392: 1736-1788. 2018/11/30. DOI: 10.1016/s0140-6736(18)32203-7.
2. Smittenaar CR, Petersen KA, Stewart K, et al. Cancer incidence and mortality projections in the UK until 2035. *Br J Cancer* 2016; 115: 1147-1155. 2016/10/26. DOI: 10.1038/bjc.2016.304.
3. Henson LA, Edmonds P, Johnston A, et al. Population-Based Quality Indicators for End-of-Life Cancer Care: A Systematic Review. *JAMA Oncol* 2019 2019/10/28. DOI: 10.1001/jamaoncol.2019.3388.
4. Ersek M, Miller SC, Wagner TH, et al. Association between aggressive care and bereaved families' evaluation of end-of-life care for veterans with non-small cell lung cancer who died in Veterans Affairs facilities. *Cancer* 2017; 123: 3186-3194. 2017/04/19. DOI: 10.1002/cncr.30700.
5. Wright AA, Keating NL, Ayanian JZ, et al. Family Perspectives on Aggressive Cancer Care Near the End of Life. *Jama* 2016; 315: 284-292. 2016/01/20. DOI: 10.1001/jama.2015.18604.
6. Wright AA, Zhang B, Keating NL, et al. Associations between palliative chemotherapy and adult cancer patients' end of life care and place of death: prospective cohort study. *Bmj* 2014; 348: g1219. 2014/03/07. DOI: 10.1136/bmj.g1219.
7. Golan R, Bernstein AN, Gu X, et al. Increased resource use in men with metastatic prostate cancer does not result in improved survival or quality of care at the end of life. *Cancer* 2018; 124: 2212-2219. 2018/03/27. DOI: 10.1002/cncr.31297.
8. Temel JS, Greer JA, Muzikansky A, et al. Early palliative care for patients with metastatic non-small-cell lung cancer. *N Engl J Med* 2010; 363: 733-742. 2010/09/08. DOI: 10.1056/NEJMoa1000678.
9. Earle CC, Landrum MB, Souza JM, et al. Aggressiveness of cancer care near the end of life: is it a quality-of-care issue? *Journal of clinical oncology : official journal of the American Society of Clinical Oncology* 2008; 26: 3860-3866. DOI: 10.1200/JCO.2007.15.8253.
10. Henson LA, Gao W, Higginson IJ, et al. Emergency department attendance by patients with cancer in their last month of life: a systematic review and meta-analysis. *J Clin Oncol* 2015; 33: 370-376. 2014/12/24. DOI: 10.1200/jco.2014.57.3568.
11. Koroukian SM, Schiltz NK, Warner DF, et al. Social determinants, multimorbidity, and patterns of end-of-life care in older adults dying from cancer. *J Geriatr Oncol* 2017; 8: 117-124. 2016/12/29. DOI: 10.1016/j.jgo.2016.10.001.
12. Mercadante S, Masedu F, Valenti M, et al. The characteristics of advanced cancer patients followed at home, but admitted to the hospital for the last days of life. *Intern Emerg Med* 2016; 11: 713-718. 2016/02/20. DOI: 10.1007/s11739-016-1402-1.
13. Abedini NC, Hechtman RK, Singh AD, et al. Interventions to reduce aggressive care at end of life among patients with cancer: a systematic review. *Lancet Oncol* 2019; 20: e627-e636. 2019/11/02. DOI: 10.1016/s1470-2045(19)30496-6.
14. Bottle A, Cohen C, Lucas A, et al. How an electronic health record became a real-world research resource: comparison between London's Whole Systems Integrated Care database and the Clinical Practice Research Datalink. *BMC Medical Informatics and Decision Making* 2020; 20: 71. DOI: 10.1186/s12911-020-1082-7.
15. NHS Digital. Business Rules for Quality and Outcomes Framework (QOF) 2017/2018, https://digital.nhs.uk/data-and-information/data-collections-and-data-sets/data-collections/quality-and-outcomes-framework-qof (2017).
16. NHS England. CCG improvement and assessment framework 2017/18, https://www.england.nhs.uk/wp-content/uploads/2017/11/ccg-improvement-and-assessment-framework-2017-18.pdf (2017, accessed September 2020).
17. Kontopantelis E, Olier I, Planner C, et al. Primary care consultation rates among people with and without severe mental illness: a UK cohort study using the Clinical Practice Research Datalink. *BMJ Open* 2015; 5: e008650. 2015/12/18. DOI: 10.1136/bmjopen-2015-008650.

For peer review only - http://bmjopen.bmj.com/site/about/guidelines.xhtml

18. Browne J, Edwards DA, Rhodes KM, et al. Association of comorbidity and health service usage among patients with dementia in the UK: a population-based study. *BMJ Open* 2017; 7: e012546. 2017/03/11. DOI: 10.1136/bmjopen-2016-012546.
19. Government DfCaL. The English Index of Multiple Deprivation (IMD) 2015 - Guidance. Ministry of Housing, Communities & Local Government., https://assets.publishing.service.gov.uk/government/uploads/system/uploads/attachment_data/file/464430/English_Index_of_Multiple_Deprivation_2015_-_Guidance.pdf (2015).
20. Almaawiy U, Pond GR, Sussman J, et al. Are family physician visits and continuity of care associated with acute care use at end-of-life? A population-based cohort study of homecare cancer patients. *Palliat Med* 2014; 28: 176-183. 2013/06/20. DOI: 10.1177/0269216313493125.
21. Seow H, Barbera L, Howell D, et al. Using more end-of-life homecare services is associated with using fewer acute care services: a population-based cohort study. *Med Care* 2010; 48: 118-124. 2010/01/09. DOI: 10.1097/MLR.0b013e3181c162ef.
22. Boot M. Exploring the district nurse role in facilitating individualised advance care planning. *Br J Community Nurs* 2016; 21: 144-147. 2016/03/05. DOI: 10.12968/bjcn.2016.21.3.144.
23. Latter S, Hopkinson JB, Lowson E, et al. Supporting carers to manage pain medication in cancer patients at the end of life: A feasibility trial. *Palliat Med* 2018; 32: 246-256. 2017/07/06. DOI: 10.1177/0269216317715197.
24. Offen J. The role of UK district nurses in providing care for adult patients with a terminal diagnosis: a meta-ethnography. *Int J Palliat Nurs* 2015; 21: 134-141. 2015/03/31. DOI: 10.12968/ijpn.2015.21.3.134.
25. Walshe C and Luker KA. District nurses' role in palliative care provision: a realist review. *Int J Nurs Stud* 2010; 47: 1167-1183. 2010/05/25. DOI: 10.1016/j.ijnurstu.2010.04.006.
26. Pivodic L, Pardon K, Miccinesi G, et al. Hospitalisations at the end of life in four European countries: a population-based study via epidemiological surveillance networks. *J Epidemiol Community Health* 2016; 70: 430-436. 2015/11/21. DOI: 10.1136/jech-2015-206073.
27. Van den Block L, Pivodic L, Pardon K, et al. Transitions between health care settings in the final three months of life in four EU countries. *Eur J Public Health* 2015; 25: 569-575. 2015/04/02. DOI: 10.1093/eurpub/ckv039.
28. Hirakawa Y, Kuzuya M and Uemura K. Opinion survey of nursing or caring staff at long-term care facilities about end-of-life care provision and staff education. *Arch Gerontol Geriatr* 2009; 49: 43-48. 2008/06/10. DOI: 10.1016/j.archger.2008.04.010.
29. Zimmerman S, Sloane PD, Hanson L, et al. Staff perceptions of end-of-life care in long-term care. *J Am Med Dir Assoc* 2003; 4: 23-26. 2003/06/17. DOI: 10.1097/01.Jam.0000046935.64053.54.
30. Kronman AC, Ash AS, Freund KM, et al. Can primary care visits reduce hospital utilization among Medicare beneficiaries at the end of life? *J Gen Intern Med* 2008; 23: 1330-1335. 2008/05/29. DOI: 10.1007/s11606-008-0638-5.
31. Bone AE, Evans CJ, Henson LA, et al. Patterns of emergency department attendance among older people in the last three months of life and factors associated with frequent attendance: a mortality follow-back survey. *Age Ageing* 2019; 48: 680-687. 2019/05/28. DOI: 10.1093/ageing/afz043.
32. Blumenthal D and Abrams MK. Tailoring Complex Care Management for High-Need, High-Cost Patients. *Jama* 2016; 316: 1657-1658. 2016/10/27. DOI: 10.1001/jama.2016.12388.
33. Salzberg CA, Hayes SL, McCarthy D, et al. Health System Performance for the High-Need Patient: A Look at Access to Care and Patient Care Experiences. *Issue Brief (Commonw Fund)* 2016; 27: 1-12. 2016/08/31.
34. Wennberg JE, Bronner K, Skinner JS, et al. Inpatient care intensity and patients' ratings of their hospital experiences. *Health Aff (Millwood)* 2009; 28: 103-112. 2009/01/07. DOI: 10.1377/hlthaff.28.1.103.
35. Green E, Knight S, Gott M, et al. Patients' and carers' perspectives of palliative care in general practice: A systematic review with narrative synthesis. *Palliat Med* 2018; 32: 838-850. 2018/01/19. DOI: 10.1177/0269216317748862.
36. Kim SL and Tarn DM. Effect of Primary Care Involvement on End-of-Life Care Outcomes: A Systematic Review. *J Am Geriatr Soc* 2016; 64: 1968-1974. 2016/10/21. DOI: 10.1111/jgs.14315.

For peer review only - http://bmjopen.bmj.com/site/about/guidelines.xhtml

37. Mitchell G, Aubin M, Senior H, et al. General practice nurses and physicians and end of life: a systematic review of models of care. *BMJ Support Palliat Care* 2020 2020/07/29. DOI: 10.1136/bmjspcare-2019-002114.
38. Carmont SA, Mitchell G, Senior H, et al. Systematic review of the effectiveness, barriers and facilitators to general practitioner engagement with specialist secondary services in integrated palliative care. *BMJ Support Palliat Care* 2018; 8: 385-399. 2017/02/16. DOI: 10.1136/bmjspcare-2016-001125.
39. Mitchell S, Loew J, Millington-Sanders C, et al. Providing end-of-life care in general practice: findings of a national GP questionnaire survey. *Br J Gen Pract* 2016; 66: e647-653. 2016/07/07. DOI: 10.3399/bjgp16X686113.
40. De Schreye R, Houttekier D, Deliens L, et al. Developing indicators of appropriate and inappropriate end-of-life care in people with Alzheimer's disease, cancer or chronic obstructive pulmonary disease for population-level administrative databases: A RAND/UCLA appropriateness study. *Palliat Med* 2017; 31: 932-945. 2017/04/22. DOI: 10.1177/0269216317705099.
41. Gallagher AM, Dedman D, Padmanabhan S, et al. The accuracy of date of death recording in the Clinical Practice Research Datalink GOLD database in England compared with the Office for National Statistics death registrations. *Pharmacoepidemiology and Drug Safety* 2019; 28: 563-569. DOI: 10.1002/pds.4747.

For peer review only

For peer review only - http://bmjopen.bmj.com/site/about/guidelines.xhtml

Supplementary material

Figure S1. Flowchart of the data management

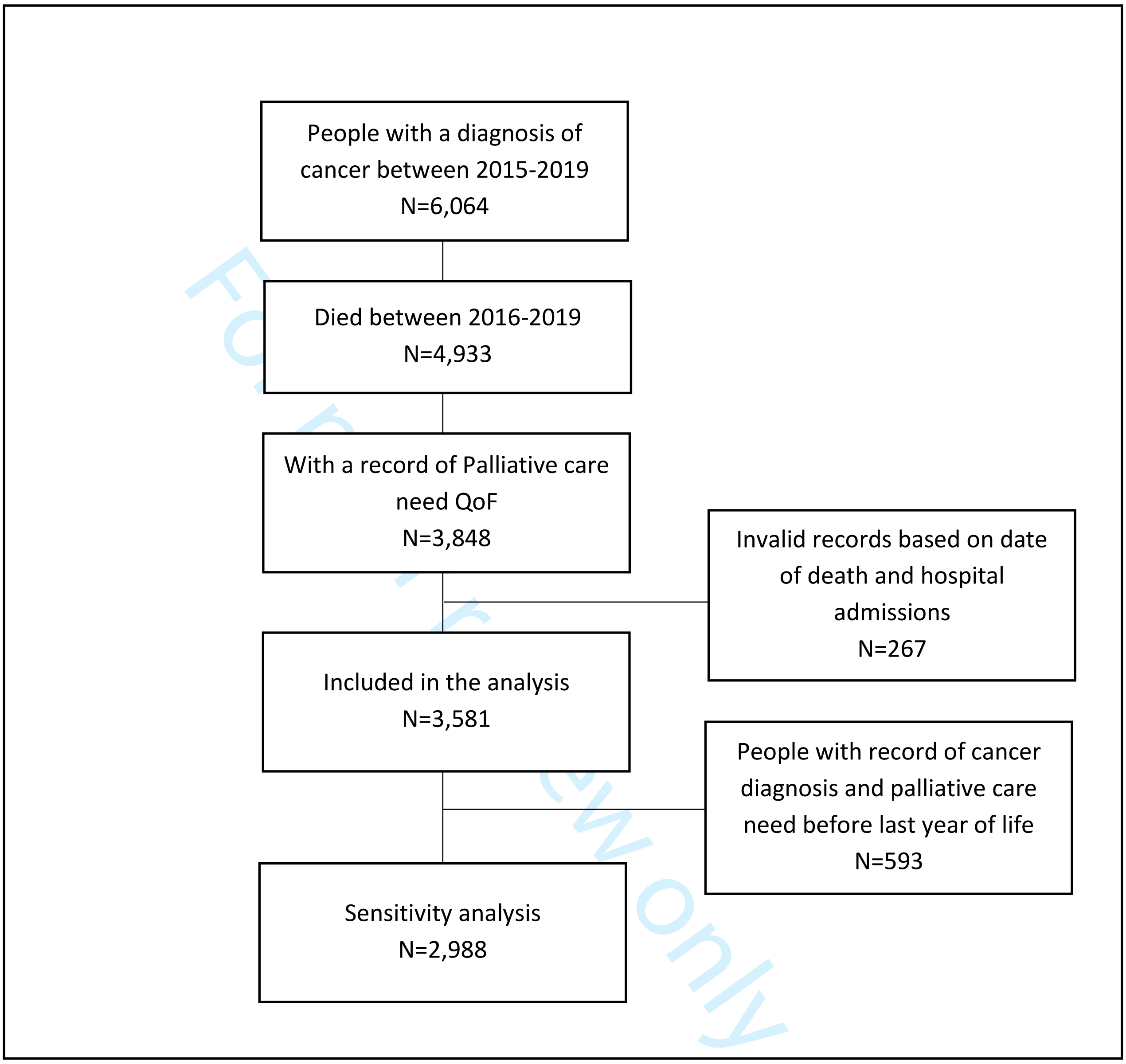

For peer review only - http://bmjopen.bmj.com/site/about/guidelines.xhtml

Box S1. List of Read codes and ICD-10 codes used to identify the cohort and variables.

	Read Codes v2	ICD10 codes
Diagnosis of cancer	B0%, B1%, B2%, B3%, B4%, B5%, B6%, Byu%, K1323, K01w1, 68W24, C184., NOT(B677.)	C00 to C97
Type of cancer		
Lung cancer	B22%	C34.0 to C34.9
Bowel cancer	B12% to B14%	C17.0 to C21.8
Prostate cancer	B45%	C61
Breast cancer	B34% to B35%	C50.0 to C50.9
Pancreas cancer	B17%	C25.0 to C25.9
Haematological cancer	B61% to B62% and B64% to B69%	C81.0 to C86.6 and C91.0 to C95.9
Date of death	22J.., 9491., 9495., 94G.., 8HG.., 9493., 94E.., 946.., 94Z.., ZV680, 94..., 949A., 949.., 9431., 9442., 9451., 9452., 9453., 946.., 9492., 9494., 9496., 9497., 9498., 9499., 949B., 949C., 949D., 949E., 949F., 949G., 949H., 949J., 949Z., 94D.., 94G..	
Palliative care QoF	1Z01. , 2JE.. , 2Jf.. , 38VY. , 38Vb. , 38Vd. , 38Ve. , 38Vf. , 38Vg. , 38Vh. , 38Vi. , 8BA2. , 8BAP. , 8BAS. , 8BAT. , 8BAe. , 8BJ1. , 8CM1.% (NOT 8CM15) , 8CM4. , 8CME. , 8CMj. , 8CMk. , 8H6A. , 8H7L. , 8H7g. , 8HH7. , 8IEE. , 9EB5. , 9Ng7. , ZV57C , 8CMQ. , 9NgD. , 9G8.. , 9c0P. , 9c0N. , 8CMW3 , 9K9.. , 9367. , 9c0L0 , 9c0M. , 9NNd. , 8CMb. , 8B2a. , 9NNf0 , 38QH. , 38QK. , 8CMg. , 2Jg.., 9NNq. , 9NNr. , 9NNs.	
Quality of Outcomes Framework (QoF) Rules		
Asthma	H33%, H3120, H3B..,173A., NOT (H333., 21262, 212G.)	
Atrial Fibrillation	G573% NOT (212R.)	
Hypertension	G2..%, G20%, Gyu2., Gyu20, G24..-G2z.., NOT (G24z1, G2400, G2410, G27..)	
Diabetes	C10.., C109J, C109K, C10C., C10D., PKyP., C10Q., C10E%, C10F%, C10H%, C10M%, C10N%, C10P% NOT(C10F8)	
Congestive heart disease	G3...-G309., G30B.-G330z, G33z.-G3401, G342.-G35X., G38..-G3z.., Gyu3%, NOT(Gyu31, G310.)	
Chronic Kidney disease	1Z12., 1Z13. , 1Z14. , 1Z15. , 1Z16. , 1Z1B.-1Z1L. , K053. , K054. , K055. , 1Z1T. , 1Z1V. , 1Z1W. , 1Z1X. , 1Z1Y. , 1Z1Z. , 1Z1a. , 1Z1b. , 1Z1c. , 1Z1d. , 1Z1e. , 1Z1f., 1Z10. , 1Z11. , 1Z17.-1Z1A., K051., K052., 1Z1M., 1Z1Q., 1Z1N., 1Z1P., 1Z1R., 1Z1S. NOT(2126E)	
COPD	H5832, H4640, H4641, Hyu30, Hyu31, H3..%, H31%, H32%, H36..-H3z.., NOT(H3101, H31y0, H3122, H3y0., H3y1.	
Depression	E0013, E0021, E118., E11y2, E11z2, E130, E135., E2003, E291., E2B.., E2B1., Eu204, Eu251, Eu341, Eu412, E112%, E113%, Eu32%, Eu33%, NOT(Eu32a, Eu32B, Eu329, 212S.)	
Epilepsy	F1321, SC200, F25%, NOT(F2501, F2504, F2511, F2516, F25y4, F25G., F25H., 21260, 212J, F256%, F258.-F25A.)	
Heart Failure	G58%, G1yz1, 662f.-662i.	
Peripheral Arterial Disease	G73.., Gyu74, G734., G73y., G73z%, NOT(G73z1)	

For peer review only

Rheumatoid Arthritis	N041., N047., N04X., N04y0, N04y2, Nyu11, Nyu12, Nyu1G, Nyu10, G5yA., G5y8., N040%, N042%, NOT(N0420)	
Stroke	G65..-G654., G656.-G65zz, G63y0.-G63y1,Gyu62-Gyu66, ZV12D, Fyu55, G6760, G6W.., G6X.., Gyu6F, Gyu6G, G61%, G64%, G66%, NOT(G617., G669.)	
Mental Health	E1124, E1134, E11z., E11z0, E11zz, E2122, Eu323, Eu328, Eu333, Eu32A, Eu329, E114.-E117z, E10%, E110%, E111%, E11y%, E12%, E13%, Eu2%, Eu30%, Eu31%, NOT(E11y2, E135.)	
Dementia	F110 to F112, E02y1, E041., Eu041, F116.,F118., F21y2, A410., Eu107, F11x7, Eu02%, E00%, Eu01%, E012%, Eu00%, A411%	

For peer review only

Box S2. List of codes used to derive primary care contacts

	Read code v2
Primary care practice face to face contacts	9N1C., 9N1w., 9NF7., 9NF8., 9k27., 9N1G., 9NFB., 9NFW., 9N1t., 9N1x., 9NF5., 9NF4., 9NF6., 982B., 982C., 9N11., 9N12., 9N1c., 9N1y0, 9N1z., 9c0H., 9N01., 9N0G., 9N7B., 9NV.., 9NY.., 9NY0.,
Primary care practice telephone contacts	9N31., 9b0m., 9b0n., 9b0o, 9N310, 9N310, 9N311, 9N3A, 8CAN., 8CAR0
Primary care practice failed contact	9N4.., 9N41%, 9Ni..

Box S3. List of codes used to derive contacts with other community care professionals

	Service description	Service reporting line
Community nurses contacts	District Nurse<br>District Nurse, Adult, Face to face<br>District Nurse, Adult, Non face to face<br>District Nurses<br>District Nursing Services: Adult<br>Cardiac Nursing / Liaison: Adult<br>Nurse<br>Nursing Services for Children<br>Other Specialist Nursing<br>Other Specialist Nursing, Adult, Face to face<br>Specialist Nursing - Asthma and Respiratory Nursing/Liaison<br>Specialist Nursing - Cardiac Nursing / Liaison<br>Specialist Nursing - Continence Services<br>Specialist Nursing - Diabetic Nursing / Liaison<br>Specialist Nursing - Parkinson''s and Alzheimers Nursing/Liaison<br>Specialist Nursing - Tissue Viability Nursing/Liaison<br>Specialist Nursing - Tuberculosis Specialist Nursing<br>Specialist Nursing, Active Case Management (Community Matrons)<br>Specialist Nursing, Asthma and Respiratory Nursing/Liaison, Adult, Face to face<br>Specialist Nursing, Asthma and Respiratory Nursing/Liaison, Adult, Non face to face<br>Specialist Nursing, Cardiac Nursing/Liaison, Adult, Face to face<br>Specialist Nursing, Cardiac Nursing/Liaison, Adult, Non face to face<br>Specialist Nursing, Continence Services<br>“Specialist Nursing, Continence Services, Adult, Face to face<br>Specialist Nursing, Continence Services, Adult, Non face to face<br>Specialist Nursing, Diabetic Nursing/Liaison<br>Specialist Nursing, Diabetic Nursing/Liaison, Adult, Face to face<br>Specialist Nursing, Diabetic Nursing/Liaison, Adult, Non face to face<br>Specialist Nursing, Parkinson''s and Alzheimers Nursing/Liaison<br>Specialist Nursing, Stoma Care Services, Adult, Face to face<br>Specialist Nursing, Tissue Viability Nursing/Liaison<br>Specialist Nursing, Tissue Viability Nursing/Liaison, Adult, Face to face<br>"Specialist Nursing, Tissue Viability Nursing/Liaison, Adult, Non face to face<br>Tissue Viability Nursing / Liaison: Adult<br>Tuberculosis Special Nursing: Adult	District Nursing<br>District Nursing (H&F, K&C, W)<br>District Nursing (H&F, K&C, W)<br>District Nursing – AWC<br>24 Hour Nursing<br>24 Hour Nursing (Phlebotomy)<br>Adult Nursing<br>CSCNS - Community Nursing<br>Children''s Community Nursing<br>Community Matron<br>Community Matron (H&F, K&C, W)<br>Community Matrons<br>Community Matrons (H&F K&C W)<br>Community Nursing<br>Heart Failure Nursing<br>Heart Nurses (K&C)<br>Night Nursing<br>Night Nursing (K&C)<br>TB Nursing<br>Tissue Viability Nursing<br>Twilight/Night Nursing Service

For peer review only

Palliative care community team contacts	Palliative / Respite Care: Adult<br>Specialist Nursing - Palliative / Respite Care<br>Specialist Nursing, Palliative/Respite Care, Adult, Face to face<br>Specialist Nursing, Palliative/Respite Care, Adult, Non face to face	Palliative Care<br>Palliative Care Service<br>Palliative Medicine (Consultant)<br>Pembridge Bereavement Counselling<br>Pembridge Community<br>Pembridge Day Care
Rehabilitation teams contacts	Community Rehabilitation Teams<br>Other Therapist, Adult, One to One<br>Physiotherapist<br>Physiotherapist, Adult, One to One<br>Physiotherapy<br>Physiotherapy Services: Adult<br>Rehabilitation for Other Disorders<br>Rehabilitation for Other Musculoskeletal Disorders<br>Rehabilitation for Other Neurological Disorders<br>Rehabilitation for Respiratory Disorders<br>SLT - Adult<br>Speech and Language Therapist, Adult, One to One<br>Speech and Language Therapy<br>Occupational Therapist, Adult, One to One<br>Occupational Therapy	Bedded Rehab – Therapists<br>Bedded Rehab - Therapists (H&F, K&C, W)<br>Brent Rehabilitation Service<br>Cardiac Rehabilitation<br>Claypond RehabTherapy<br>Community IFC MSK Physiotherapy Service<br>Community MSK Physiotherapy Service<br>Community Neuro Rehab<br>Community Neuro-Rehabilitation (H&F, K&C, W)<br>Community Recovery Service - Neuro Rehab<br>Community Rehab ICE<br>Community Rehabilitation<br>Community Rehabilitation (H&F, K&C, W)<br>EDTC - Community Physio<br>EHT Therapies<br>Ealing Hospital Therapies<br>Integrated Rehab<br>MSK Physiotherapy<br>Musculoskeletal Service<br>Musculoskeletal Service (W)<br>Physio (MSK)<br>Physiotherapy<br>Pulmonary Rehab<br>Short Term Rehabilitation<br>Therapies MS Physio<br>Adult SLT<br>SLT (Adults)<br>Occupational Therapy

For peer review only

Table S1. Sensitivity analysis for three or more hospital admissions in the last 90 days

	Model 1		Model 2		Model 3		Model 4		Model 5	
	Original Model		Model 1 without days in hospital		Model 1 including contacts with PC practice as continu-ous		Model 1 only for people with a record of cancer diagnosis and identifica-tion of palliative care needs in the last 12 months of life		Model 1 with ethnicity	
	n=3472		n=3472		n=3472		n=2703		n-=2841	
	RR	95% CI	RR	95% CI	RR	95% CI	RR	95% CI	RR	95% CI
Age	0.98	(0.97 to 0.99)	0.98	(0.97 to 0.98)	0.98	(0.97 to 0.98)	0.98	(0.97 to 0.98)	0.98	(0.97 to 0.99)
Gender (Male vs female)	1.10	(0.92 to 1.31)	1.11	(0.92 to 1.34)	1.10	(0.92 to 1.02)	1.05	(0.87 to 1.27)	1.06	(0.88 to 1.28)
IMD quintile (Ref=1)										
2	0.82	(0.65 to 1.03)	0.87	(0.70 to 1.09)	0.81	(0.65 to 1.02)	0.87	(0.67 to 1.12)	0.83	(0.65 to 1.05)
3	0.89	(0.70 to 1.12)	0.87	(0.67 to 1.12)	0.88	(0.70 to 1.12)	0.87	(0.66 to 1.13)	0.83	(0.64 to 1.09)
4	0.96	(0.75 to 1.24)	0.90	(0.68 to 1.17)	0.97	(0.75 to 1.24)	1.01	(0.77 to 1.33)	0.97	(0.73 to 1.30)
5	0.95	(0.68 to 1.31)	0.80	(0.58 to 1.11)	0.93	(0.67 to 1.29)	0.90	(0.62 to 1.30)	0.85	(0.56 to 1.28)
Living in care home (Yes vs No)	0.53	(0.28 to 0.98)	0.54	(0.29 to 0.98)	0.52	(0.28 to 0.98)	0.54	(0.28 to 1.05)	0.64	(0.34 to 1.20)
Type of cancer (Ref=Bowel)										
Lung	1.60	(1.16 to 2.20)	1.51	(1.10 to 2.09)	1.61	(1.17 to 2.21)	1.68	(1.17 to 2.41)	1.63	(1.16 to 2.28)
Prostate	1.57	(1.07 to 2.30)	1.81	(1.22 to 2.67)	1.57	(1.07 to 2.30)	1.55	(1.01 to 2.38)	1.64	(1.08 to 2.51)
Breast	1.24	(0.82 to 1.87)	1.33	(0.86 to 2.03)	1.24	(0.82 to 1.87)	1.27	(0.77 to 2.08)	1.15	(0.71 to 1.84)
Pancreas	1.39	(0.93 to 2.08)	1.33	(0.87 to 2.05)	1.42	(0.95 to 2.13)	1.51	(0.96 to 2.37)	1.47	(0.95 to 2.28)
Haematological	1.23	(0.73 to 2.07)	1.82	(1.11 to 2.98)	1.23	(0.73 to 2.08)	1.19	(0.65 to 2.16)	1.13	(0.62 to 2.05)
Other	1.15	(0.83 to 1.58)	1.22	(0.87 to 1.69)	1.15	(0.84 to 1.58)	1.23	(0.86 to 1.78)	1.19	(0.84 to 1.69)
Number of QoF comorbidities (Ref=0)										
1	0.88	(0.69 to 1.11)	0.97	(0.75 to 1.25)	0.87	(0.68 to 1.10)	0.87	(0.67 to 1.13)	0.88	(0.66 to 1.17)
2	0.99	(0.76 to 1.30)	1.08	(0.81 to 1.44)	0.98	(0.75 to 1.29)	0.96	(0.71 to 1.29)	0.89	(0.65 to 1.20)
3	1.23	(0.94 to 1.62)	1.38	(1.04 to 1.83)	1.24	(0.94 to 1.63)	1.18	(0.87 to 1.60)	1.14	(0.84 to 1.55)
>=4	0.98	(0.74 to 1.30)	1.17	(0.88 to 1.57)	0.97	(0.74 to 1.28)	0.91	(0.66 to 1.25)	0.91	(0.66 to 1.26)
Dementia (Yes vs No)	0.78	(0.54 to 1.14)	0.77	(0.53 to 1.13)	0.79	(0.54 to 1.14)	0.80	(0.51 to 1.27)	0.76	(0.49 to 1.16)
Contacts with the primary care practice (Ref= 0 to 3)										
4 to 10	1.18	(0.98 to 1.41)	1.16	(0.97 to 1.39)			1.15	(0.94 to 1.39)	1.20	(0.98 to 1.47)
>=11	1.63	(1.33 to 1.99)	1.52	(1.23 to 1.88)			1.64	(1.32 to 2.04)	1.77	(1.41 to 2.22)
Number of contacts with primary care practice (continuous)					1.02	(1.01 to 1.03)				
Number of days in hospital in the last 90 days	1.04	(1.03 to 1.04)			1.04	(1.03 to 1.04)	1.04	(1.03 to 1.04)	1.04	(1.03 to 1.04)
Ethnicity (Ref= white)										
Black									1.11	(0.82 to 1.50)
Asian									1.52	(1.21 to 1.90)
Mixed									1.19	(0.93 to 1.51)
Other									1.08	(0.73 to 1.60)

Table S2. Sensitivity analysis for one or more hospital admissions in the last 30 days

	Model 1		Model 2		Model 3		Model 4		Model 5	
	Original Model n=3441		Model 1 without days in hospital n=3441		Model 1 including contacts with primary care practice as continuous n=3441		Model 1 for people with a record of cancer diagnosis and identification of palliative care needs in the last 12 months of life n=2679		Model 1 with ethnicity n-=2815	
	RR	95% CI	RR	95% CI	RR	95% CI	RR	95% CI	RR	95% CI
Age	1.00	(0.99 to 0.99)	0.99	(0.99 to 1.00)	1.00	(0.99 to 1.00)	1.00	(0.99 to 1.00)	1.00	(0.99 to 1.00)
Gender (Male vs female)	1.11	(1.02 to 1.20)	1.11	(1.02 to 1.21)	1.11	(1.03 to 1.21)	1.10	(1.00 to 1.20)	1.08	(0.99 to 1.19)
IMD quintile (Ref=1)										
2	1.01	(0.91 to 1.12)	1.03	(0.92 to 1.14)	1.01	(0.91 to 1.12)	1.03	(0.92 to 1.16)	1.05	(0.93 to 1.18)
3	1.03	(0.92 to 1.15)	1.02	(0.91 to 1.15)	1.02	(0.92 to 1.14)	1.06	(0.93 to 1.19)	1.05	(0.92 to 1.17)
4	0.96	(0.85 to 1.09)	0.94	(0.82 to 1.07)	0.96	(0.85 to 1.08)	0.99	(0.86 to 1.13)	0.95	(0.82 to 1.09)
5	1.06	(0.90 to 1.25)	1.02	(0.86 to 1.20)	1.06	(0.90 to 1.26)	1.08	(0.91 to 1.28)	1.06	(0.86 to 1.29)
Living in care home (Yes vs no)	0.54	(0.41 to 0.72)	0.54	(0.40 to 0.71)	0.54	(0.41 to 0.73)	0.58	(0.43 to 0.79)	0.58	(0.44 to 0.80)
Type of cancer (Ref=Bowel)										
Lung	1.09	(0.96 to 2.23)	1.06	(0.93 to 1.21)	1.08	(0.95 to 1.23)	1.06	(0.91 to 1.23)	1.06	(0.92 to 1.22)
Prostate	0.98	(0.84 to 2.15)	1.04	(0.89 to 1.22)	0.98	(0.83 to 1.14)	0.94	(0.78 to 1.14)	1.05	(0.87 to 1.24)
Breast	1.19	(1.02 to 1.39)	1.21	(1.03 to 1.43)	1.18	(1.01 to 1.38)	1.22	(1.02 to 1.47)	1.17	(0.96 to 1.40)
Pancreas	1.02	(0.87 to 2.20)	1.01	(0.85 to 1.20)	1.03	(0.87 to 1.21)	1.05	(0.88 to 1.25)	1.07	(0.90 to 1.29)
Haematological	0.91	(0.72 to 2.15)	1.04	(0.83 to 1.31)	0.91	(0.71 to 1.15)	0.85	(0.65 to 1.11)	0.94	(0.73 to 1.21)
Other	0.96	(0.86 to 1.08)	0.98	(0.87 to 1.11)	0.96	(0.85 to 1.08)	0.94	(0.82 to 1.07)	1.00	(0.87 to 1.14)
Number of QoF comorbidities (Ref=0)										
1	0.92	(0.83 to 1.02)	0.96	(0.86 to 1.07)	0.92	(0.83 to 1.02)	0.93	(0.83 to 1.05)	0.93	(0.81 to 1.05)
2	1.06	(0.95 to 1.18)	1.10	(0.98 to 1.23)	1.06	(0.95 to 1.18)	1.12	(1.00 to 1.26)	1.02	(0.89 to 1.16)
3	0.99	(0.87 to 1.12)	1.03	(0.91 to 1.18)	0.98	(0.87 to 1.11)	0.98	(0.85 to 1.13)	0.99	(0.84 to 1.14)
>=4	1.11	(0.97 to 1.27)	1.19	(1.04 to 1.36)	1.10	(0.96 to 1.26)	1.11	(0.94 to 1.30)	1.07	(0.92 to 1.24)
Dementia (Yes vs No)	0.94	(0.82 to 1.07)	0.91	(0.80 to 1.05)	0.93	(0.82 to 1.07)	0.91	(0.78 to 1.06)	0.95	(0.81 to 1.09)
COPD (Yes vs No)	1.07	(0.97 to 1.19)	1.05	(0.95 to 1.17)	1.07	(0.97 to 1.18)	1.08	(0.96 to 1.20)	1.11	(0.98 to 1.25)
Contacts with community nurses (Ref= 0 to 3)										
4 to 12	1.06	(0.98 to 1.15)	1.05	(0.97 to 1.14)			1.07	(0.98 to 1.18)	1.03	(0.94 to 1.14)
>=13	0.88	(0.90 to 0.98)	0.85	(0.76 to 0.95)			0.88	(0.78 to 0.98)	0.84	(0.76 to 0.92)
Contacts with community palliative care teams (Ref= 0 to 3)										
4 to 8	0.95	(0.82 to 1.08)	0.91	(0.80 to 1.04)			0.94	(0.81 to 1.09)	0.90	(0.77 to 1.06)
>=9	0.85	(0.69 to 1.04)	0.79	(0.63 to 0.99)			0.92	(0.74 to 1.14)	0.89	(0.70 to 1.12)
Days in hospital in the last 90 days	1.02	(1.01 to 1.02)			1.02	(1.01 to 1.02)	1.01	(1.01 to 1.02)	1.02	(1.01 to 1.02)
Contacts with community nurses (Continuous)					1.00	(0.99 to 1.00)				
Contacts with community palliative care teams (Continuous)					0.99	(0.97 to 1.00)				
Ethnicity (Ref= white)										
Black									1.12	(0.97 to 1.31)
Asian									1.17	(1.06 to 1.30)
Mixed									1.14	(1.03 to 1.28)
Other									0.91	(0.76 to 1.09)

Table S3. Sensitivity analysis for one or more ED visits in the last 2 weeks of life

	Model 1		Model 2		Model 3		Model 5		Model 5	
	Original Model		Model 1 without days in hospital		Model 1 including contacts with PC practice as continu-ous		Model 1 for people with a record of cancer diagnosis and identification of pallia-tive care needs in the last 12 months of life		Model 1 with ethnicity	
	n=3441		n=3441		n=3441		n=2679		n-=2815	
	RR	95% CI	RR	95% CI	RR	95% CI	RR	95% CI	RR	95% CI
Age	0.99	(0.99 to 0.99)	0.99	(0.99 to 1.00)	0.99	(0.99 to 1.00)	0.99	(0.99 to 1.00)	0.99	(0.99 to 1.00)
Gender (Male vs female)	1.10	(0.98 to 1.23)	1.10	(0.98 to 1.23)	1.10	(0.98 to 1.23)	1.02	(0.89 to 1.17)	1.09	(0.96 to 1.24)
IMD quintile (Ref=1)										
2	1.01	(0.89 to 1.15)	1.01	(0.89 to 1.15)	1.00	(0.88 to 1.15)	1.03	(0.88 to 1.20)	1.03	(0.88 to 1.21)
3	0.93	(0.80 to 1.15)	0.93	(0.79 to 1.09)	0.93	(0.79 to 1.08)	0.91	(0.75 to 1.10)	0.94	(0.79 to 1.13)
4	1.00	(0.84 to 1.18)	0.99	(0.83 to 1.17)	1.00	(0.84 to 1.18)	0.99	(0.81 to 1.22)	0.97	(0.79 to 1.18)
5	0.99	(0.79 to 1.22)	0.97	(0.78 to 1.21)	0.98	(0.78 to 1.19)	1.03	(0.81 to 1.30)	0.93	(0.70 to 1.23)
Living in care home (Yes vs no)	0.70	(0.49 to 0.99)	0.69	(0.49 to 0.98)	0.70	(0.49 to 1.19)	0.71	(0.49 to 1.02)	0.76	(0.51 to 1.13)
Type of cancer (Ref=Bowel)										
Lung	1.01	(0.85 to 1.19)	1.00	(0.84 to 1.19)	1.01	(0.85 to 1.20)	1.03	(0.85 to 1.26)	0.99	(0.82 to 1.21)
Prostate	0.89	(0.72 to 1.10)	0.90	(0.73 to 1.11)	0.89	(0.72 to 1.09)	0.92	(0.71 to 1.19)	0.91	(0.71 to 1.16)
Breast	1.16	(0.94 to 1.42)	1.17	(0.95 to 1.43)	1.16	(0.94 to 1.42)	1.19	(0.92 to 1.52)	1.14	(0.90 to 1.44)
Pancreas	0.82	(0.63 to 1.06)	0.82	(0.63 to 1.06)	0.82	(0.63 to 1.06)	0.80	(0.60 to 1.07)	0.83	(0.63 to 1.11)
Haematological	0.93	(0.68 to 1.26)	0.97	(0.71 to 1.31)	0.94	(0.69 to 1.27)	0.84	(0.57 to 1.24)	0.90	(0.63 to 1.27)
Other	0.82	(0.70 to 0.96)	0.83	(0.71 to 0.96)	0.83	(0.71 to 0.96)	0.84	(0.69 to 1.01)	0.84	(0.70 to 1.00)
Number of QoF comorbidities (Ref=0)										
1	0.88	(0.75 to 1.04)	0.89	(0.75 to 1.05)	0.88	(0.74 to 1.03)	0.94	(0.78 to 1.13)	0.88	(0.73 to 1.06)
2	1.05	(0.89 to 1.25)	1.06	(0.89 to 1.26)	1.05	(0.89 to 1.25)	1.08	(0.89 to 1.31)	0.95	(0.78 to 1.16)
3	1.01	(0.84 to 1.21)	1.02	(0.85 to 1.23)	1.01	(0.84 to 1.22)	1.03	(0.83 to 1.28)	0.97	(0.79 to 1.20)
>=4	1.09	(0.89 to 1.32)	1.10	(0.90 to 1.34)	1.09	(0.89 to 1.32)	1.11	(0.88 to 1.39)	1.04	(0.83 to 1.30)
Chronic Heart Disease (yes vs no)	1.14	(0.99 to 1.31)	1.14	(1.00 to 1.31)	1.13	(0.99 to 1.30)	1.13	(0.97 to 1.31)	1.16	(1.00 to 1.34)
Contacts with the primary care practice (Ref= 0 to 3)										
4 to 10	1.10	(0.98 to 1.22)	1.10	(0.98 to 1.22)			1.13	(1.00 to 1.27)	1.17	(1.04 to 1.31)
>=11	1.27	(1.10 to 1.47)	1.27	(1.10 to 1.47)			1.35	(1.15 to 1.57)	1.35	(1.14 to 1.60)
Contacts with community nurses (Ref= 0 to 3)										
4 to 12	0.96	(0.85 to 1.08)	0.96	(0.85 to 1.08)			0.92	(0.80 to 1.06)	0.99	(0.87 to 1.14)
>=13	0.79	(0.68 to 0.92)	0.78	(0.67 to 0.91)			0.80	(0.68 to 0.93)	0.74	(0.62 to 0.87)
Contacts with community palliative care teams (Ref= 0 to 3)										
4 to 8	1.01	(0.85 to 1.21)	1.00	(0.84 to 1.20)			1.01	(0.81 to 1.26)	0.91	(0.72 to 1.15)
>=9	0.78	(0.56 to 1.08)	0.76	(0.55 to 1.06)			0.90	(0.66 to 1.24)	0.75	(0.49 to 1.12)
Days in hospital in the last 90 days	1.00	(1.00 to 1.01)			1.00	(1.00 to 1.00)	1.00	(1.00 to 1.01)	1.00	(1.00 to 1.01)
Contacts with primary care practice (continuous)					1.01	(1.01 to 1.01)				
Contacts with community nurses (Continuous)					0.99	(0.99 to 0.99)				
Contacts with community palliative care teams (Continuous)					0.99	(0.97 to 1.01)				
Ethnicity (Ref= white)										
Black									1.28	(1.05 to 1.55)

Asian					1.29	(1.11 to 1.50)
Mixed					0.99	(0.84 to 1.17)
Other					1.00	(0.76 to 1.29)

For peer review only

STROBE Statement—Checklist of items that should be included in reports of ***cohort studies***

	Item No	Recommendation	Page No
**Title and abstract**	1	(*a*) Indicate the study's design with a commonly used term in the title or the abstract	1
		(*b*) Provide in the abstract an informative and balanced summary of what was done and what was found	2
**Introduction**			
Background/rationale	2	Explain the scientific background and rationale for the investigation being reported	3
Objectives	3	State specific objectives, including any prespecified hypotheses	3
**Methods**			
Study design	4	Present key elements of study design early in the paper	3-4
Setting	5	Describe the setting, locations, and relevant dates, including periods of recruitment, exposure, follow-up, and data collection	3-4
Participants	6	(*a*) Give the eligibility criteria, and the sources and methods of selection of participants. Describe methods of follow-up	3-4
		(*b*) For matched studies, give matching criteria and number of exposed and unexposed	n/a
Variables	7	Clearly define all outcomes, exposures, predictors, potential confounders, and effect modifiers. Give diagnostic criteria, if applicable	5-6
Data sources/ measurement	8*	For each variable of interest, give sources of data and details of methods of assessment (measurement). Describe comparability of assessment methods if there is more than one group	5-6 and supplementary material
Bias	9	Describe any efforts to address potential sources of bias	4-6
Study size	10	Explain how the study size was arrived at	7 and supplementary material
Quantitative variables	11	Explain how quantitative variables were handled in the analyses. If applicable, describe which groupings were chosen and why	4-6
Statistical methods	12	(*a*) Describe all statistical methods, including those used to control for confounding	6
		(*b*) Describe any methods used to examine subgroups and interactions	6
		(*c*) Explain how missing data were addressed	6
		(*d*) If applicable, explain how loss to follow-up was addressed	n/a
		(*e*) Describe any sensitivity analyses	6
**Results**			

For peer review only

Participants	13*	(a) Report numbers of individuals at each stage of study—eg numbers potentially eligible, examined for eligibility, confirmed eligible, included in the study, completing follow-up, and analysed	7 and supplementary material
		(b) Give reasons for non-participation at each stage	7 and supplementary material
		(c) Consider use of a flow diagram	supplementary material
Descriptive data	14*	(a) Give characteristics of study participants (eg demographic, clinical, social) and information on exposures and potential confounders	7 and table 1
		(b) Indicate number of participants with missing data for each variable of interest	7 and table 1
		(c) Summarise follow-up time (eg, average and total amount)	4-6, 8-10
Outcome data	15*	Report numbers of outcome events or summary measures over time	7-9 and table 1
Main results	16	(*a*) Give unadjusted estimates and, if applicable, confounder-adjusted estimates and their precision (eg, 95% confidence interval). Make clear which confounders were adjusted for and why they were included	8-9, table 3
		(*b*) Report category boundaries when continuous variables were categorized	9 and table 2 and 3
		(*c*) If relevant, consider translating estimates of relative risk into absolute risk for a meaningful time period	table 2 and 3
Other analyses	17	Report other analyses done—eg analyses of subgroups and interactions, and sensitivity analyses	11 and supplementary material
**Discussion**			
Key results	18	Summarise key results with reference to study objectives	14
Limitations	19	Discuss limitations of the study, taking into account sources of potential bias or imprecision. Discuss both direction and magnitude of any potential bias	15-16
Interpretation	20	Give a cautious overall interpretation of results considering objectives, limitations, multiplicity of analyses, results from similar studies, and other relevant evidence	14-15
Generalisability	21	Discuss the generalisability (external validity) of the study results	15-16
**Other information**			
Funding	22		17

*Give information separately for exposed and unexposed groups.

Note: An Explanation and Elaboration article discusses each checklist item and gives methodological background and published examples of transparent reporting. The STROBE checklist is best used in conjunction with this article (freely available on the Web sites of PLoS Medicine at http://www.plosmedicine.org/, Annals of Internal Medicine at http://www.annals.org/, and Epidemiology at http://www.epidem.com/). Information on the STROBE Initiative is available at http://www.strobe-statement.org.

For peer review only - http://bmjopen.bmj.com/site/about/guidelines.xhtml
